# Identification of trypsin-degrading commensals in the large intestine

**DOI:** 10.1038/s41586-022-05181-3

**Published:** 2022-09-07

**Authors:** Youxian Li, Eiichiro Watanabe, Yusuke Kawashima, Damian R. Plichta, Zhujun Wang, Makoto Ujike, Qi Yan Ang, Runrun Wu, Munehiro Furuichi, Kozue Takeshita, Koji Yoshida, Keita Nishiyama, Sean M. Kearney, Wataru Suda, Masahira Hattori, Satoshi Sasajima, Takahiro Matsunaga, Xiaoxi Zhang, Kazuto Watanabe, Jun Fujishiro, Jason M. Norman, Bernat Olle, Shutoku Matsuyama, Ho Namkoong, Yoshifumi Uwamino, Makoto Ishii, Koichi Fukunaga, Naoki Hasegawa, Osamu Ohara, Ramnik J. Xavier, Koji Atarashi, Kenya Honda

**Affiliations:** 1 RIKEN Center for Integrative Medical Sciences, Yokohama, Japan; 2 Department of Microbiology and Immunology, Keio University School of Medicine, Tokyo, Japan; 3 Institute for Cancer Research, Faculty of Medicine, University of Oslo, Oslo, Norway; 4 Department of Pediatric Surgery, Faculty of Medicine, The University of Tokyo, Tokyo, Japan; 5 Department of Applied Genomics, Kazusa DNA Research Institute, Kisarazu, Japan; 6 Infectious Disease and Microbiome Program, Broad Institute of MIT and Harvard, Cambridge, MA USA; 7 Faculty of Veterinary Medicine, Nippon Veterinary and Life Science University, Tokyo, Japan; 8 Interdisciplinary Life Science—PULSe, Purdue University, West Lafayette, IN USA; 9 Cooperative Major in Advanced Health Science, Graduate School of Advanced Science and Engineering, Waseda University, Tokyo, Japan; 10 JSR-Keio University Medical and Chemical Innovation Center, Keio University School of Medicine, Tokyo, Japan; 11 Vedanta Biosciences, Cambridge, MA USA; 12 Department of Virology III, National Institute of Infectious Diseases, Tokyo, Japan; 13 Department of Infectious Diseases, Keio University School of Medicine, Tokyo, Japan; 14 Department of Laboratory Medicine, Keio University School of Medicine, Tokyo, Japan; 15 Division of Pulmonary Medicine, Department of Medicine, Keio University School of Medicine, Tokyo, Japan; 16 Department of Molecular Biology, Massachusetts General Hospital, Boston, MA USA; 17 Center for Computational and Integrative Biology, Massachusetts General Hospital, Harvard Medical School, Boston, MA USA

**Keywords:** Mucosal immunology, Microbiome

## Abstract

Increased levels of proteases, such as trypsin, in the distal intestine have been implicated in intestinal pathological conditions^1–3^. However, the players and mechanisms that underlie protease regulation in the intestinal lumen have remained unclear. Here we show that *Paraprevotella* strains isolated from the faecal microbiome of healthy human donors are potent trypsin-degrading commensals. Mechanistically, *Paraprevotella* recruit trypsin to the bacterial surface through type IX secretion system-dependent polysaccharide-anchoring proteins to promote trypsin autolysis. *Paraprevotella* colonization protects IgA from trypsin degradation and enhances the effectiveness of oral vaccines against *Citrobacter rodentium*. Moreover, *Paraprevotella* colonization inhibits lethal infection with murine hepatitis virus-2, a mouse coronavirus that is dependent on trypsin and trypsin-like proteases for entry into host cells^4,5^. Consistently, carriage of putative genes involved in trypsin degradation in the gut microbiome was associated with reduced severity of diarrhoea in patients with SARS-CoV-2 infection. Thus, trypsin-degrading commensal colonization may contribute to the maintenance of intestinal homeostasis and protection from pathogen infection.

## Abstract (web summary)

Colonization of trypsin-degrading commensal bacteria may contribute to the maintenance of intestinal homeostasis and protection against pathogen infection in humans and mice.

## Main

The gastrointestinal tract is a unique organ that is constitutively exposed to countless dietary, microbiota-derived and host-derived molecules, including digestive enzymes. Digestive enzymes have essential roles in breaking down dietary macronutrients into smaller components in the upper intestine. However, in the large intestine, they are unneeded and their dysregulated activity has been implicated in changes in microbiota composition, disruption of mucosal barrier integrity and incidence of inflammation^1–3,6,7^. To maintain homeostasis and barrier integrity, intestinal tissue implements a variety of regulatory and protective mechanisms, such as the production of mucin and enzyme-inactivating molecules^8–10^. Moreover, the gut microbiota contributes substantially to maintaining a stable environment by depleting or modifying luminal materials^11–13^. However, it remains unclear how and what microorganisms control digestive enzymes.

## Regulation of trypsin by the microbiota

**Fig. 1:**
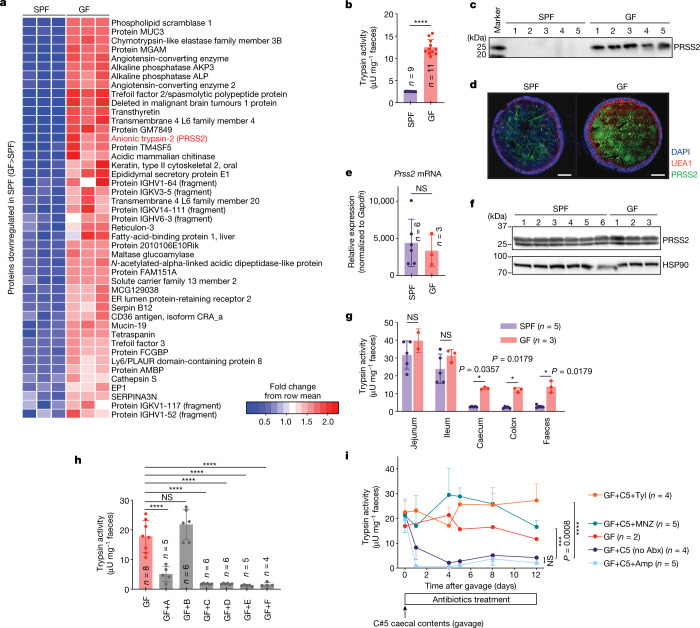
Microbiota-mediated regulation of trypsin in the large intestine. **a**, Proteins with reduced levels in the caecum of SPF mice compared with in the caecum of GF mice, as determined by proteome analysis. **b**, Faecal trypsin activity in SPF mice compared with in GF mice. **c**, Western blot analysis of trypsin (PRSS2) in the faeces of SPF and GF mice. **d**, Immunostaining of colon sections of SPF and GF mice. Blue, DAPI; green, PRSS2; red, UEA1 (mucus). **e**,**f**, *Prss2* expression levels in the pancreas of SPF or GF mice measured using quantitative PCR with reverse transcription (RT–qPCR) (**e**) and western blotting (**f**). Heat-shock protein 90 (HSP90) was the loading control. **g**, Trypsin activity of intestinal contents at the indicated locations. **h**, Faecal trypsin activity of GF mice or GF mice inoculated with faecal samples from the indicated healthy donors (A–F). **i**, Trypsin activity in faeces of GF mice after inoculation with the caecal contents of mouse C5 and concomitant treatment with antibiotics (Abx) or vehicle control. For **b**, **e** and **g**–**i**, data are mean ± s.d. Each dot represents one mouse (**b**, **e**, **g** and **h**). Statistical analysis was performed using two-sided Mann–Whitney *U*-tests with Welch’s correction (nonparametric) (**b**, **e** and **g**) and one-way analysis of variance (ANOVA) with Tukey’s test (**h** and **i**); ****P < 0.0001, ***P < 0.001, *P < 0.05; NS, not significant. For **d**, scale bar, 500 μm. For **c**, a representative image from two independent experiments with similar results is shown. For **f**, images from one experiment including all of the mice used in **e** are shown. Blot source data are provided in Supplementary Fig. 1. Source data

**Extended Data Fig. 1:**
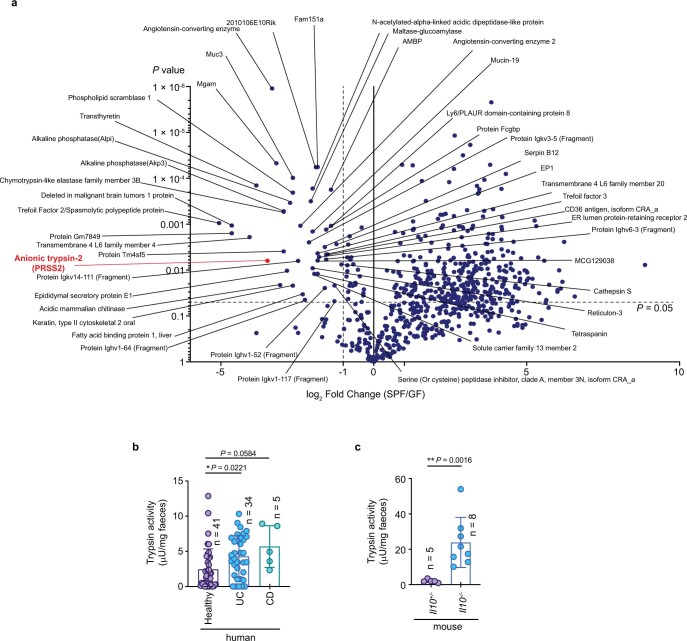
Elevated trypsin levels in germ free (GF) mice, and in humans and mice with intestinal inflammation. Distribution of all host-derived proteins detected in the proteome analysis of the caecal contents from specific-pathogen-free (SPF) or GF mice, with protein relative abundance plotted against *p* value. Anionic trypsin 2 (PRSS2) is highlighted in red. See Supplementary Table 1 for the complete list of proteins detected. **b**, Faecal trypsin activity of healthy controls, ulcerative colitis (UC) and Crohn’s disease (CD) patients. **c**, Faecal trypsin activity of *Il10*^+/−^ and *Il10*^−/−^ mice. **b**, **c**, Data shown as mean ± s.d. Each dot represents one human subject or one mouse. ** *p* < 0.01; * *p* < 0.05. One-way ANOVA with Tukey’s test (**b**) and two-sided Mann-Whitney test with Welch’s correction (nonparametric) (**c**). Source data

To examine the influence of the gut microbiota on the landscape of colonic luminal proteins, including digestive enzymes, caecal contents were collected from germ-free (GF) and specific-pathogen-free (SPF) mice and analysed using unbiased mass spectrometry (MS)-based proteomics^14^. Out of the 713 host-derived proteins detected (Supplementary Table 1), 324 were found to be higher in SPF mice compared with in GF mice, including immune-related molecules, whereas 45 molecules were more abundant in GF mice than in SPF mice (greater than twofold, *P* < 0.05) (Fig. 1a and Extended Data Fig. 1a), including the mouse anionic isoform of trypsin protease (encoded by *Prss2*). The marked difference in trypsin levels between GF and SPF mice was confirmed by a trypsin-activity assay, western blotting and immunostaining analysis (Fig. 1b–d). We examined trypsinogen production in the pancreas (Fig. 1e,f) and luminal trypsin activity at different sites of the intestine (Fig. 1g), and differential levels of trypsin between GF and SPF mice were detected only in the large intestine (Fig. 1g). These data suggest that trypsin is probably regulated by microbiota members in the large intestine.

## Trypsin-degrading commensals

**Extended Data Fig. 2:**
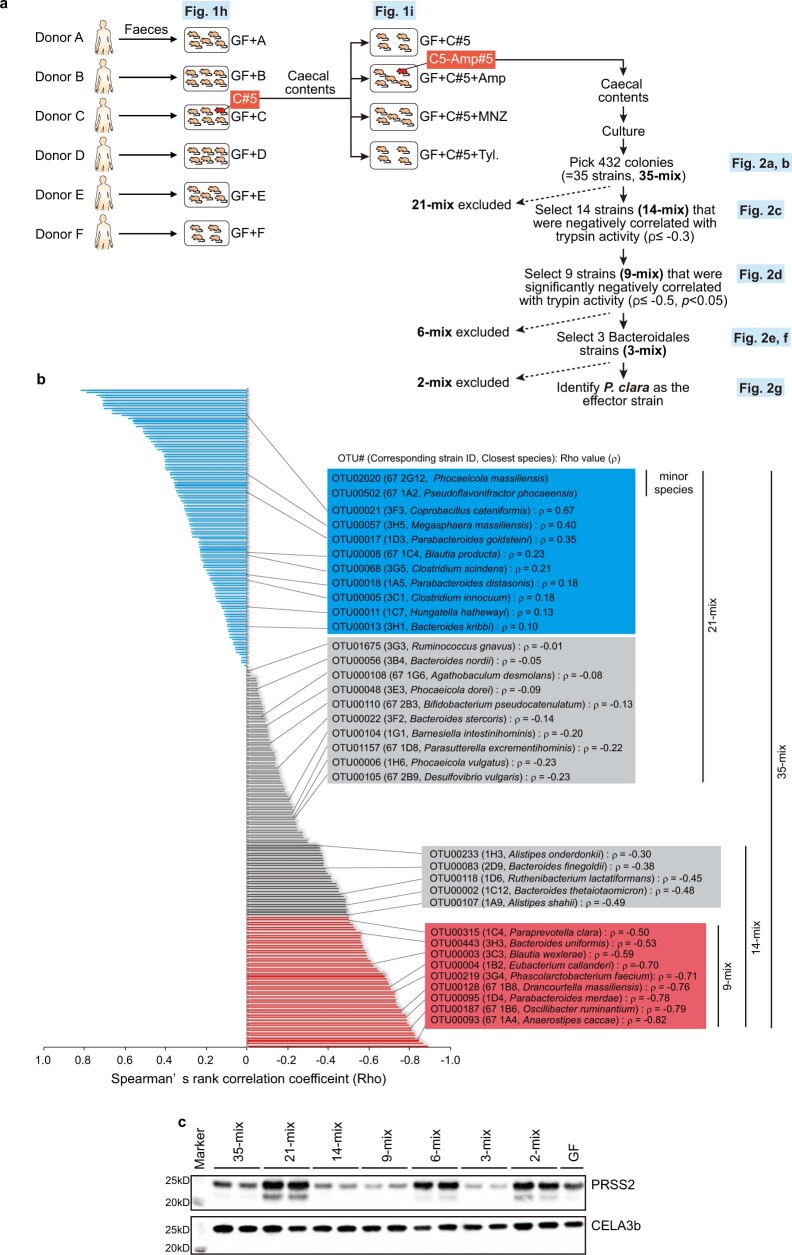
Reduced faecal trypsin levels in gnotobiotic mice colonized with bacterial mixtures containing *P. clara*. **a**, Schematic representation of the strategy for isolating trypsin-reducing bacteria from the healthy human gut microbiota. The caecal contents from a GF mouse colonized with the donor C microbiota and receiving ampicillin treatment were cultured anaerobically on various types of agar plates containing different growth media including EG, ES, M10, NBGT, VS, TS, BL, BBE, Oxoid CM0619, CM0619-supplemented SR0107, CM0619-supplemented SR0108, mGAM and Schaedler. 432 bacterial colonies were picked and sequenced. The 35 strains identified were subjected to further rounds of gnotobiotic and *in vitro* screening until identification of *P. clara* as the effector strain. **b**, Spearman’s correlation coefficient quantifying the association between relative abundance and trypsin activity for individual bacterial OTUs detected in mice in Fig. 2a. Operational taxonomic units (OTUs) significantly negatively correlated (ρ ≤ −0.5, *p* < 0.05), negatively but not significantly correlated, and positively correlated with trypsin activity are marked in red, grey and blue, respectively. **c**, Western Blot analysis of mouse trypsin (PRSS2) in the faeces from GF mice colonized with the indicated bacterial mixtures. **c**, Images from one experiment are shown. See Supplementary Figure 1 for blot source data. Source data

Healthy humans and mice tend to have low faecal trypsin levels^2,3^, whereas faecal samples from both humans with inflammatory bowel disease and *Il10*-deficient colitogenic mice had higher trypsin activities (Extended Data Fig. 1b,c), suggesting the potential importance of microbiota-mediated regulation of trypsin. The ability of the intestinal microbiota to inactivate pancreatic proteases has been suggested in earlier reports, but the effector bacteria are undefined^15–20^. We set out to isolate and identify trypsin-reducing species from the human microbiota. Faecal samples from six healthy Japanese donors (donors A–F) were transplanted into GF mice (Extended Data Fig. 2a). Faecal microbiota from donors A, C, D, E and F effectively reduced faecal trypsin activity in recipient mice (Fig. 1h). We selected a mouse (C5) from the donor C microbiota recipient group and gavaged its caecal contents into a new set of GF mice (GF+C5 mice). To narrow down the microbial community, the GF+C5 mice were divided into four groups and treated with ampicillin (Amp), metronidazole (MNZ), tylosin (Tyl) or a vehicle control (with no antibiotics) through the drinking water. Faecal trypsin activity was decreased in GF+C5 mice without antibiotic treatment and was further reduced by Amp treatment, whereas treatment with MNZ or Tyl abrogated this reduction (Fig. 1i), suggesting that C5 microbiota contained trypsin-reducing species that were enriched in the Amp-treated group and reduced in the MNZ- and Tyl-treated groups.

**Fig. 2:**
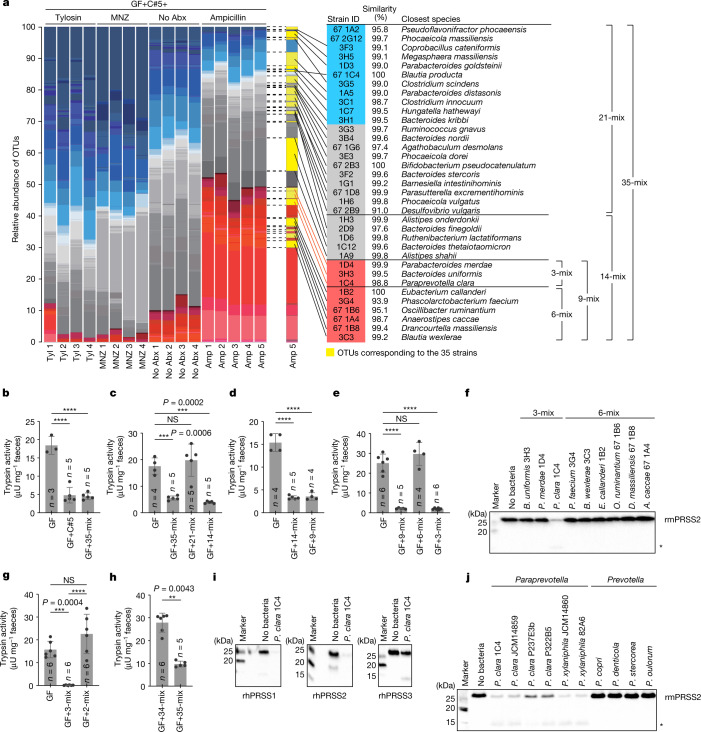
Identification of *Paraprevotella* as trypsin-degrading species. **a**, The caecal microbiota composition of individual mice determined by 16S rRNA gene sequencing. Operational taxonomic units (OTUs) significantly negatively correlated (*ρ* ≤ −0.5, *P* < 0.05), negatively but not significantly correlated, and positively correlated with trypsin activity are marked in red, grey and blue, respectively. OTUs corresponding to the 35 strains isolated from the caecal contents of mouse C5-Amp#5 are marked in yellow and their closest species and percentage similarity in the NCBI-RefSeq 16S rRNA gene database are shown. **b**–**e**,**g**,**h**, The faecal trypsin activity of mice colonized with the indicated bacterial mixtures. **f**,**j**, Recombinant mouse trypsin (rmPRSS2) was in vitro incubated with individual strains of the 9-mix (**f**) or the indicated *Paraprevotella* or *Prevotella* strains (**j**), and degradation of rmPRSS2 was analysed using western blotting. The asterisk indicates the cleaved fragment of rmPRSS2. **i**, Recombinant human trypsin isoforms PRSS1, PRSS2 and PRSS3 (rhPRSS1–3) were incubated with *P. clara* 1C4 and degradation of human trypsin was analysed using western blotting. For **b**–**e**, **g** and **h**, data are mean ± s.d. Each dot represents one mouse. Statistical analysis was performed using two-sided Mann–Whitney *U*-tests with Welch’s correction (nonparametric) (**h**) and one-way ANOVA with Tukey’s test (**b**–**e** and **g**); ****P < 0.0001, ***P < 0.001, **P < 0.01. For **f**, **i** and **j**, representative images from two independent experiments with similar results are shown. Blot source data are provided in Supplementary Fig. 1. Source data

We followed up on one of the Amp-treated mice (mouse C5-Amp#5) and cultured its caecal contents in a variety of media under anaerobic conditions (Extended Data Fig. 2a). We picked 432 distinct colonies and analysed them using 16S rRNA gene sequencing to elaborate 35 unique strains that broadly covered the bacterial species colonizing the C5-Amp#5 mouse (Fig. 2a). Introduction of a mixture of the 35 isolated bacteria (35-mix) into GF mice (GF+35-mix) reproduced the marked decrease in faecal trypsin activity (Fig. 2b). Among the 35 strains, the relative abundances of 14 strains in the faecal microbiota in mice from the aforementioned antibiotic study (Fig. 2a) were negatively associated with trypsin activity (*ρ* ≤ −0.3) (Extended Data Fig. 2b). The colonization of GF mice with these 14 strains (GF+14-mix) induced a robust reduction of faecal trypsin, whereas GF mice colonized with the other 21 strains (GF+21-mix) showed no reduction (Fig. 2c and Extended Data Fig. 2c). A further selection of 9 strains (9-mix) that were significantly associated with a reduction in trypsin activity (*ρ* ≤ −0.5, *P* < 0.05) out of the 14-mix similarly reduced trypsin activity (Fig. 2d and Extended Data Fig. 2c). We next divided the 9-mix into a 3-mix consisting of Bacteroidales species and a 6-mix consisting of non-Bacteroidales species. The 3-mix was sufficient to decrease faecal trypsin activity (Fig. 2e and Extended Data Fig. 2c). In vitro incubation of the individual strains of the 9-mix with recombinant mouse trypsin (rmPRSS2, with a C-terminal His-tag) revealed that *Paraprevotella* *clara* (strain ID: 1C4) was the only strain with the ability to reduce the amount of trypsin (Fig. 2f). Consistently, GF mice colonized with the 2-mix (excluding *P. clara* from the 3-mix) or the 34-mix (excluding *P. clara* from the 35-mix) showed defects in reducing trypsin activity (Fig. 2g,h), confirming that *P. clara* is the effector strain out of the 35-mix.

The small fragment recognized by the anti-His-tag antibody indicates trypsin degradation by *P. clara* (Fig. 2f,j). Degradation also occurred when *P. clara* was incubated with the three known isoforms of human trypsin (PRSS1 and PRSS2 and, to a lesser extent, PRSS3) (Fig. 2i). *Paraprevotella* is a recently identified genus under the family Prevotellaceae, containing only two species, *P. clara* and *Paraprevotella xylaniphila*^21^. We examined several *P. clara* and *P. xylaniphila* strains, as well as species from the phylogenetically related *Prevotella* genus, and we found that the trypsin-degrading property is conserved in all *Paraprevotella* strains but is absent in the tested *Prevotella* strains (Fig. 2j).

## Molecules involved in trypsin degradation

**Fig. 3:**
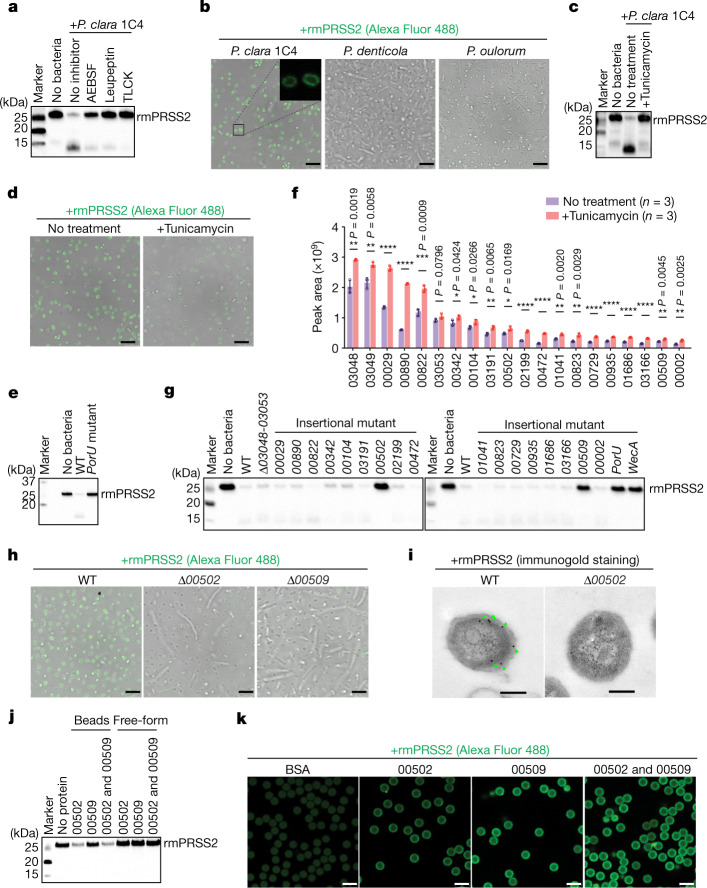
Identification of effector molecules responsible for *Paraprevotella*-mediated trypsin degradation. **a**, Recombinant mouse trypsin (rmPRSS2) pretreated with the indicated protease inhibitors was incubated with *P. clara* 1C4, and degradation of rmPRSS2 was analysed using western blotting. **b**, Alexa Fluor 488-labelled rmPRSS2 (green) was incubated with the indicated species, and association of rmPRSS2 with the bacterial surface was examined using confocal microscopy. The black square indicates the region magnified in the top right, showing *P. clara* cells. **c**,**d**, rmPRSS2 degradation (**c**) and association with the bacterial surface (**d**) after incubation with *P. clara* 1C4 pretreated with tunicamycin  or vehicle control. **e**, rmPRSS2 degradation mediated by WT or *PorU-*mutant *P. clara* JCM14859. **f**, *P. clara* proteins with elevated levels in the culture supernatants after tunicamycin treatment, as determined by proteome analysis. **g**, rmPRSS2 degradation mediated by WT or the indicated mutants of *P. clara* JCM14859. **h**, Association of rmPRSS2 with the surface of WT or the indicated deletion mutants of *P. clara* JCM14859. **i**, Transmission electron microscopy images of WT or *Δ00502* strains incubated with rmPRSS2. The green arrowheads indicate immunogold-labelled rmPRSS2. **j**, rmPRSS2 degradation after incubation with microbead-coupled or free-form recombinant 00502 and/or 00509. **k**, Association of rmPRSS2 with microbead-coupled recombinant 00502 and/or 00509 or albumin control (BSA). For **f**, data are mean ± s.d. Each dot represents one technical replicate. Statistical analysis was performed using two-sided multiple unpaired *t*-tests (not corrected for multiple comparisons); ****P < 0.0001, ***P < 0.001, **P < 0.01, *P < 0.05. Scale bars, 5 μm (**b**, **d**, **h** and **k**) and 200 nm (**i**). For **a**–**e** and **g**–**k**, representative images from two independent experiments with similar results (**a**–**e**, **g**, **h**, **j** and **k**) or images from one experiment (**i**) are shown. Blot source data are provided in Supplementary Fig. 1. Source data

**Extended Data Fig. 3:**
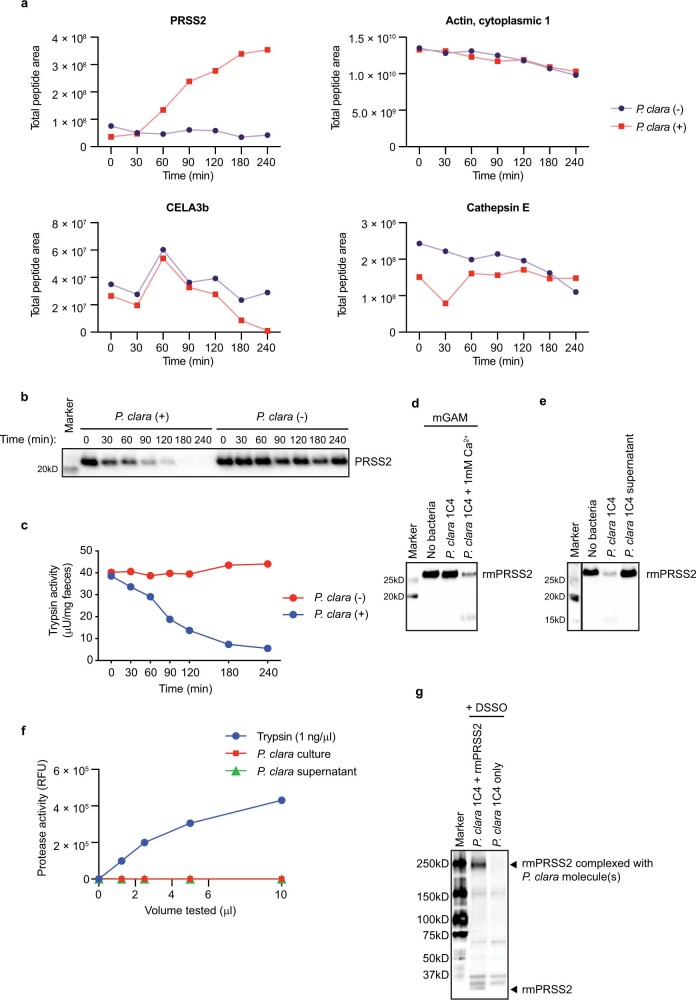
Initial mechanistic studies of *Paraprevotella*-mediated trypsin degradation. **a**, GF mouse caecal contents were incubated with *P. clara* 1C4 [*P. clara* (+)] or medium control [*P. clara* (−)]. Supernatant samples were collected at the indicated time points and subjected to peptidome analysis. Changes in levels of peptides derived from representative mouse proteins are shown. See Supplementary Table 2 for the complete list of peptides detected. **b**, **c**, GF mouse caecal contents (contain high levels of trypsin) were incubated with *P. clara* 1C4 [*P. clara* (+)] or medium control [*P. clara* (−)]. Trypsin (PRSS2) levels were analysed by Western Blot (**b**) or by trypsin activity assay (**c**) at the indicated time points. **d**, His-tagged recombinant mouse trypsin (rmPRSS2) was incubated with *P. clara* 1C4 cultured in a low-calcium medium (mGAM) or in mGAM supplemented with 1mM Ca^2+^ and degradation of rmPRSS2 was analysed by Western Blot with anti-His-tag antibody. **e**, rmPRSS2 was incubated with *P. clara* 1C4 or filtered *P. clara* 1C4 supernatant, and degradation of rmPRSS2 was analysed by Western Blot with anti-His-tag antibody. **f**, Protease activity of overnight live *P. clara* 1C4 culture or filtered *P. clara* 1C4 supernatant as determined by cleavage of FITC-labelled casein. Trypsin (1 ng μl^−1^) was used as the positive control. Protease activity was expressed as change in relative fluorescence units (RFU). **g**, *P. clara* 1C4 was incubated with rmPRSS2 and then treated with disuccinimidyl sulfoxide (DSSO) cross-linker. The cross-linked interaction complex between rmPRSS2 and *P. clara*-derived molecules was analysed by Western blot with anti-His tag antibody. *P. clara* 1C4 without incubation with rmPRSS2 (*P. clara* 1C4 only) was used as the negative control. **b**, **d**, **e**, **g**, Representative images from two (**d**, **e**) or three (**g**) independent experiments with similar results, or an image from one experiment (**b**) are shown. See Supplementary Figure 1 for blot source data. Source data

Ex vivo incubation of GF caecal contents with *P. clara* showed a gradual loss of trypsin and an increase in trypsin-derived peptides (Extended Data Fig. 3a–c). The liquid chromatography coupled with MS (LC–MS)-based peptidome analysis revealed no *P. clara* substrates other than trypsin (Extended Data Fig. 3a and Supplementary Table 2). *P. clara*-mediated trypsin degradation occurred only in the presence of divalent cations (such as Ca^2+^) (Extended Data Fig. 3d). Thus, the degradation appears to be enzyme (protease) mediated. However, *P. clara* culture supernatant did not degrade trypsin (Extended Data Fig. 3e), and no proteolytic activity was detected in live *P. clara* or in the supernatant (Extended Data Fig. 3f). Instead, pretreatment of trypsin with trypsin inhibitors (AEBSF, leupeptin and TLCK) inhibited its degradation by *P. clara* (Fig. 3a), suggesting that the degradation is mediated by trypsin-dependent autolysis. Moreover, fluorescently labelled trypsin was found to accumulate on the surface of *P. clara* (Fig. 3b). Thus, trypsin degradation probably occurs on the surface of *P. clara* through trypsin-binding surface molecules that facilitate trypsin accumulation and autolysis.

**Extended Data Fig. 4:**
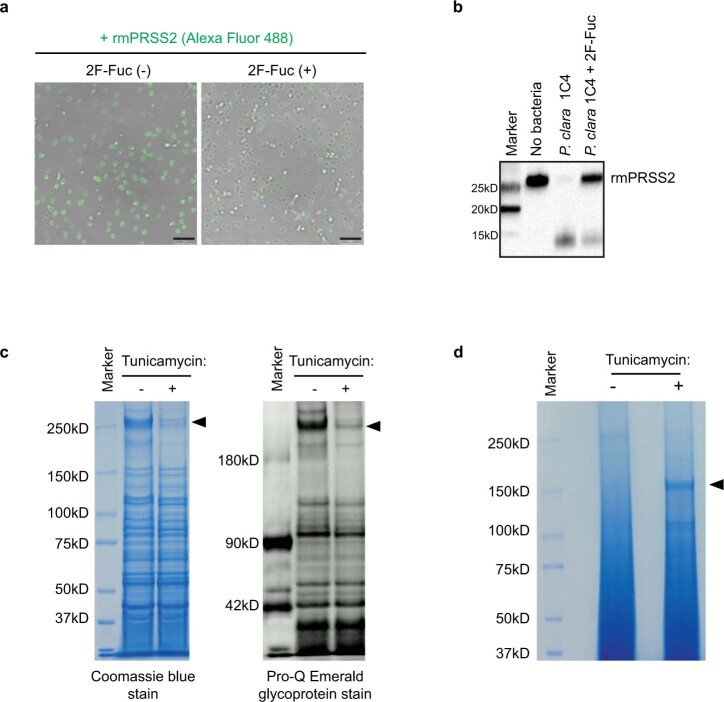
Shedding of *Paraprevotella* proteins into the supernatant following treatment with tunicamycin. **a**, **b**, *P. clara* 1C4 was pre-treated with 2F-Fuc [2F-Fuc (+)] or vehicle control [2F-Fuc (−)] followed by incubation with rmPRSS2. Association of rmPRSS2 with *P. clara* 1C4 was examined by confocal microscopy (**a**) and degradation of rmPRSS2 was analysed by Western Blot with anti-His-tag antibody (**b**). Scale bar: 5 μm (**a**). **c**, *P. clara* 1C4 was treated with tunicamycin or vehicle control, and whole cell lysates were analysed for protein (**left**) and glycan (**right**) contents with Colloidal Coomassie Blue staining and Pro-Q Emerald 300 staining, respectively. **d**, Supernatant proteins from samples in (**c**) were analysed with Colloidal Coomassie Blue staining. Arrowheads indicate the bands that were decreased (**c**) or increased (**d**) after tunicamycin treatment. **a**–**d**, Representative images from two independent experiments with similar results are shown. See Supplementary Figure 1 for gel and blot source data.

**Extended Data Fig. 5:**
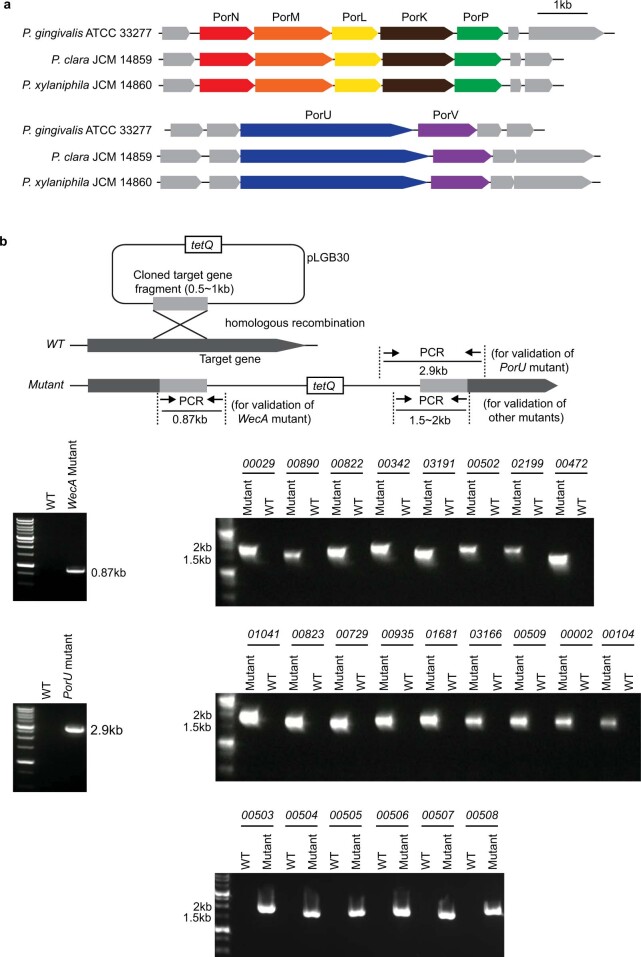
Type IX secretion system (T9SS) components in *Paraprevotella* genomes and generation of insertional mutants for *P. clara* JCM14859. **a**, Alignment of T9SS gene components in the genomes of *P. clara* JCM14859, *P. xylaniphila* JCM14860 and *P. gingivalis* ATCC33277. **b**, Schematic illustration of insertional mutagenesis by plasmid integration. PCR validation results for the indicated mutants are shown. Primers used for mutagenesis and PCR validation are listed in Supplementary Table 5. pLGB30: suicide vector used for cloning and integrating sequences into *P. clara* JCM14859. **b**, Images from one experiment are shown. See Supplementary Figure 1 for gel source data.

We used disuccinimidyl sulfoxide (DSSO), a chemical cross-linker, to examine molecules on *P. clara* that interact with His-tagged trypsin. DSSO treatment resulted in the emergence of a new band with a high molecular mass (around 250 kDa) blotted by an anti-His-tag antibody, indicative of a trypsin-containing complex (Extended Data Fig. 3g). The smeared appearance of the band suggests that trypsin interacts with molecules that are heterogenous in size. Bacteroidetes (in which *Paraprevotella* is included) are known to decorate their cell surface with complex glycans^22,23^. We therefore used inhibitors to target glycan synthesis in *P. clara*, reasoning that glycan-binding molecules are possible trypsin-binding partners. *P. clara* pretreated with tunicamycin, which inhibits synthesis of lipopolysaccharide (LPS) *O*-glycans^24^, showed defects in the recruitment and degradation of trypsin (Fig. 3c,d). Similar results were obtained when *P. clara* was treated with 2-fluro-l-fucose, which broadly inhibits the synthesis of fucose-containing glycans (Extended Data Fig. 4a,b). Treatment with tunicamycin led to a loss of glycan-containing proteins from the cell lysate (Extended Data Fig. 4c) and elevated protein shedding into the supernatant (Extended Data Fig. 4d). This was reminiscent of what was reported for LPS-deficient *Porphyromonas gingivalis* mutants, which were unable to anchor type IX secretion system (T9SS)-dependent outer membrane proteins (for example, gingipains) to LPS on the surface^25–27^. The T9SS is a bacterial machinery that transports proteins bearing a conserved C-terminal domain (CTD) across the outer membrane to the surface, removes the CTD and mediates the attachment of the exported proteins to surface polysaccharides^28^. Putative T9SS genes were identified in the genomes of *Paraprevotella* strains (Extended Data Fig. 5a). We therefore hypothesized that surface proteins secreted by the T9SS might be responsible for the recruitment and degradation of trypsin. To test this, we generated a mutant *P. clara* deficient for *PorU* (an essential component of the T9SS) by inserting a plasmid sequence into the gene locus (Extended Data Fig. 5b). Disruption of *PorU* led to a complete defect in trypsin degradation (Fig. 3e).

**Extended Data Fig. 6:**
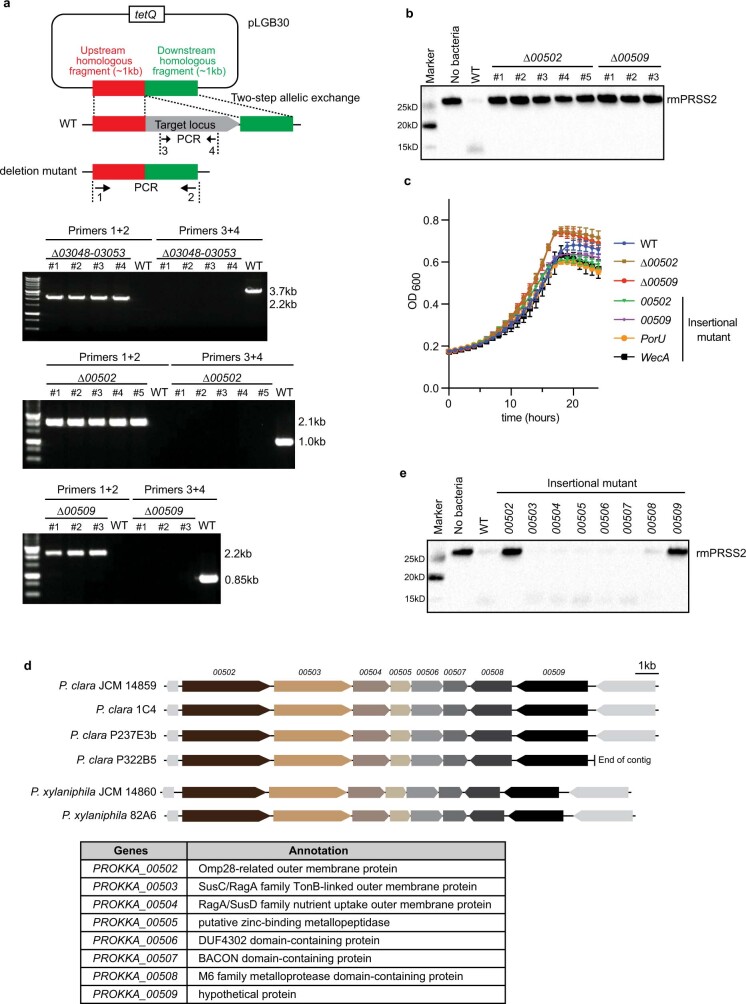
Generation of gene deletion mutants of *P. clara* JCM14859, growth of mutants deficient in trypsin degradation and analysis of genes located between *00502* and *00509*. **a**, Schematic illustration (upper panel) and PCR validation (lower panels) of *03048-03053*, *00502* and *00509* gene deletion. Primers used for mutagenesis and PCR validation are listed in Supplementary Table 5. pLGB30: suicide vector used for cloning and integrating sequences into *P. clara* JCM14859. **b**, Wild type (WT), *Δ00502* or *Δ00509 P. clara* JCM14589 strains were incubated with recombinant mouse PRSS2 (rmPRSS2) and degradation of rmPRSS2 was analysed by Western Blot with anti-His-tag antibody. **c**, *In vitro* growth rate of mutants deficient in trypsin degradation as determined by OD_600_. : **d**, Alignment of the *00502*-*00509* gene cluster in *Paraprevotella* genomes and annotation of each protein with Prokka 1.14.6. **e**, Wild type (WT) or the indicated mutant strains of *P. clara* JCM14589 were incubated with rmPRSS2, and degradation of rmPRSS2 was analysed by Western Blot. **c**, Data shown as mean ± s.d. n = 5 wells of individual bacterial cultures per group. **a**, **b**, **e**, Images from one experiment are shown. See Supplementary Figure 1 for gel and blot source data. Source data

We next conducted a proteome analysis of *P. clara* culture supernatants in the presence or absence of tunicamycin and found 20 bacterial proteins that were significantly elevated in the supernatant of tunicamycin-treated *P. clara* (Fig. 3f). Thus, we generated a series of mutant *P. clara* strains disrupting the synthesis of these tunicamycin-sensitive proteins by insertional mutagenesis (Extended Data Fig. 5b) or by deletion of a gene cluster (*Δ03048*-*03053*) (Extended Data Fig. 6a). Disruption of the gene encoding PROKKA_00502 (Omp28-related outer membrane protein) or PROKKA_00509 (hypothetical protein) resulted in the abrogation of trypsin degradation in vitro, similar to in *PorU-*deficient or *WecA-*deficient (target of tunicamycin) mutants (Fig. 3g). In addition to insertional mutants, we generated *P. clara* deletion mutants for *00502* and *00509* (*Δ00502* and *Δ00509*) (Extended Data Fig. 6a), and both strains showed severe defects in the recruitment (Fig. 3h,i) and degradation of trypsin in vitro (Extended Data Fig. 6b). Mutants defective in *PorU*, *WecA*, *00502* and *00509* displayed no growth defects (Extended Data Fig. 6c), indicating that trypsin degradation is not essential for in vitro bacterial growth. The *00502*-*00509* locus is conserved in all tested *Paraprevotella* strains (Extended Data Fig. 6d). However, the *00503*-*00508* genes separating *00502* and *00509* were not required for trypsin degradation (Extended Data Figs. 5b and 6e).

**Extended Data Fig. 7:**
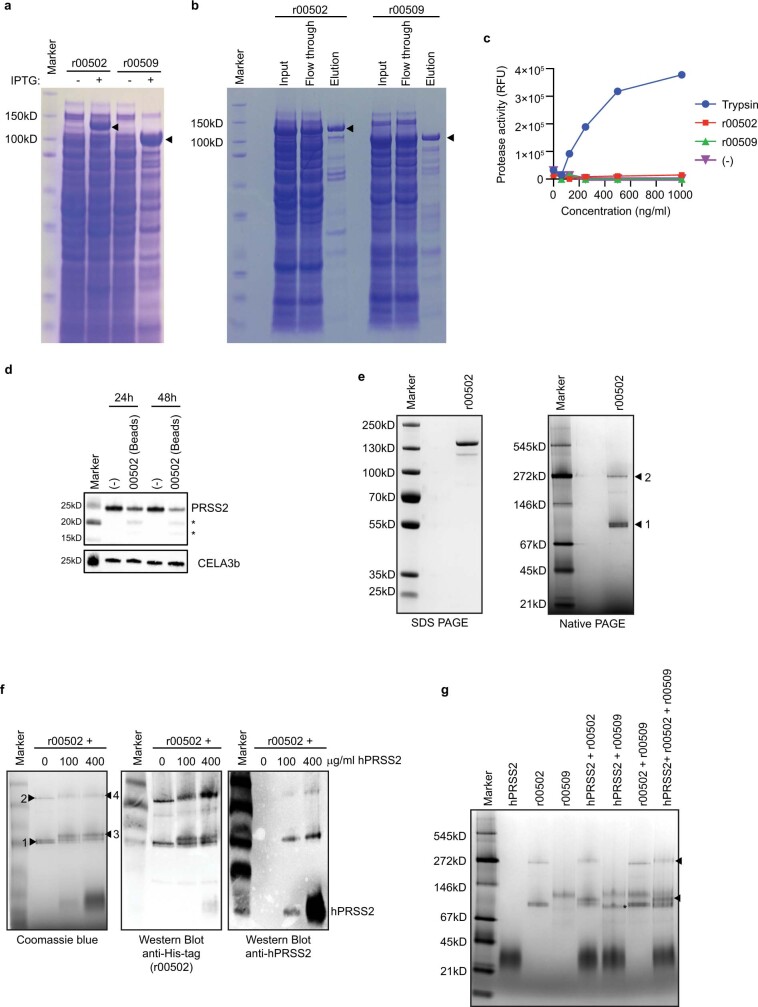
Generation of recombinant PROKKA_00502 (r00502) and PROKKA_00509 (r00509), and assessment of their trypsin-binding and -degrading properties. **a**, **b**, *E. coli* hosts carrying expression vectors for r00502 or r00509 were treated with IPTG to induce recombinant protein expression (**a**), and the expressed r00502 or r00509 were purified from cell lysates (**b)**. Protein contents of the whole cell lysates (‘Input’ and ‘Flow through’) or purified recombinants (‘Eluted’) were analysed with Coomassie Blue staining. Arrows indicate protein bands of r00502 or r00509 with the predicted molecular weights. **c**, Protease activity of r00502 or r00509 as determined by cleavage of FITC-labelled casein. Trypsin was used as the positive control. (-): no protein added. Protease activity was expressed as change in relative fluorescence units (RFU). **d**, Caecal contents from germ-free (GF) mice were incubated with medium control (-) or beads coupled with recombinant 00502 [00502 (beads)], and ex vivo degradation of trypsin was analysed by Western Blot at the indicated time points with anti-mouse PRSS2 antibody. * Cleaved fragments of PRSS2. **e**, SDS-PAGE (**left**) and Native PAGE (**right**) analysis of the purified r00502. Arrows indicate the monomer (1) and the possible oligomer form (2) of r00502 on a native PAGE gel. **f**, r00502 was incubated with recombinant human trypsin (hPRSS2, pretreated with trypsin inhibitor AEBSF) at the indicated concentrations at room temperature for 20 min, the reaction mix was analysed by a native PAGE and then subject to Coomassie Blue staining (**left**) or Western Blot analysis using antibodies against r00502 (anti-His-tag, **middle**) and hPRSS2 (**right**). Arrows indicate the bands corresponding to r00502 monomer (1), r00502 oligomer (2), r00502 monomer complexed with hPRSS2 (3) and r00502 oligomer complexed with hPRSS2 complex (4) that were excised for proteomic analysis (Supplementary Table 3). The marker used here is designed for SDS-PAGE-based chemiluminescent Western blot and does not reflect the actual molecular weight on a Native PAGE gel. It was used only for the purpose of alignment of the individual bands between the gel and the blots. **g**, Native PAGE analysis and Coomassie Blue staining of the recombinant proteins incubated alone or as mixtures at room temperature for 20 min. hPRSS2 was pre-treated with AEBSF to inhibit the trypsin activity. Arrows indicate the migration shifts of the r00502 bands when hPRSS2 was present. * degraded fragment of r00509 by hPRSS2. **a**, **b**, **d**–**g**, Representative images from two independent experiments with similar results are shown. See Supplementary Figure 1 for gel and blot source data. Source data

We next generated recombinant 00502 and 00509 proteins (Extended Data Fig. 7a,b). No protease activity was detected for the recombinant proteins (Extended Data Fig. 7c), and free-form 00502 or 00509 did not degrade trypsin (Fig. 3j). Coupling recombinant 00502 to microbeads enabled effective recruitment and in vitro degradation of recombinant trypsin (Fig. 3j,k), as well as ex vivo degradation of trypsin in GF caecal contents (Extended Data Fig. 7d). 00509-coupled beads facilitated trypsin recruitment but not degradation (Fig. 3j,k). These results suggest that 00502 functions as a core effector component for trypsin recruitment and autodegradation, whereas 00509 probably has a supporting role in facilitating trypsin recruitment.

**Extended Data Fig. 8:**
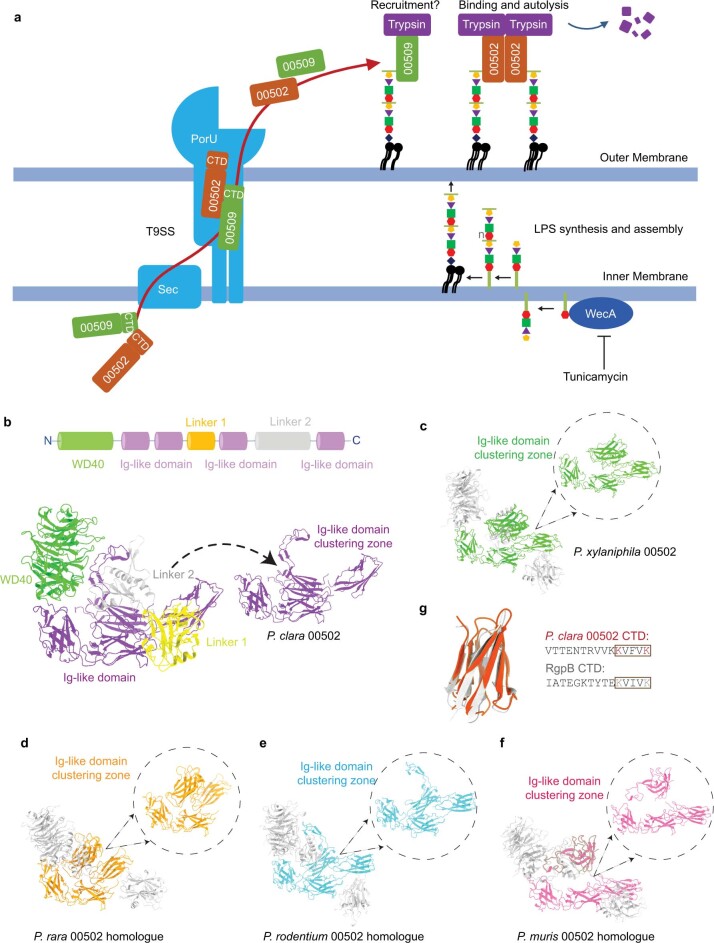
Model of *Paraprevotella*-mediated trypsin degradation and structural predictions of 00502. **a**, Model of *Paraprevotella*-mediated trypsin degradation: 00502 and 00509 proteins are transported across the outer membrane of *Paraprevotella* via the Type IX secretion system (T9SS). PorU is an essential T9SS component that cleaves the C-terminal domain (CTD) of T9SS-dependent proteins and anchors the proteins to *Paraprevotella* LPS molecules. WecA mediates the initial step of LPS O-glycan synthesis, and disruption of WecA function (e.g., with tunicamycin treatment) causes release of T9SS-dependent proteins. 00502 acts as a core effector component, facilitating trypsin association and auto-degradation possibly mediated by 00502 oligomerization, whereas 00509 may play a supporting and dispensable role in facilitating trypsin recruitment. Sec: Sec system that exports proteins across the cytoplasmic membrane. **b**, AlphaFold2-based structural prediction of *P. clara* 00502 protein with individual domains highlighted. Four out of the five Ig-like domains are shown; the last Ig-like domain that serves as the T9SS C-terminal target domain therefore does not form part of the Ig-like domain clustering zone is omitted here (shown separately in panel **g**). **c**–**f**, AlphaFold2-based structural prediction of *P. xylaniphila*, *P. rara*, *P. rodentium* and *P. muris* 00502 homologues with the conserved Ig-like domain clustering zone highlighted. **g**, Alignment of the C-terminal domain (CTD) of *P. clara* 00502 with that of *Porphyromonas gingivalis* RgpB protein. The “KXXXK” motif is a signature of T9SS C-terminal target domain-containing proteins.

Recombinant 00502 showed two distinct bands on a native PAGE gel: one corresponds to the monomer form and the other probably corresponds to oligomers (Extended Data Fig. 7e). After incubation with trypsin, both bands shifted upwards (Extended Data Fig. 7f), suggesting that trypsin forms complexes with either form of 00502. Western blot analysis (Extended Data Fig. 7f) and in-gel MS/MS analysis (Supplementary Table 3) confirmed recovery of both 00502 and trypsin from these bands. We found no bands indicative of oligomer or complex formation for 00509 on a native PAGE gel (Extended Data Fig. 7g). These data suggest that 00502 tends to oligomerize, and oligomerized 00502 possibly brings multiple trypsin molecules together to facilitate autolysis (Extended Data Fig. 8a). We predicted the structure of 00502s from *P. clara* and *P. xylaniphila* using AlphaFold2. The resulting model is composed of an N-terminal WD40 domain with five immunoglobin (Ig)-like domains (Extended Data Fig. 8b,c). These Ig-like domains are well conserved among 00502 proteins of *Paraprevotella* species and could be binding sites for trypsin. The Ig-like domain at the C terminus aligns well with CTD of the gingipain RgpB, a known T9SS target^29^ (Extended Data Fig. 8g).

## *P. clara* maintains IgA

**Fig. 4:**
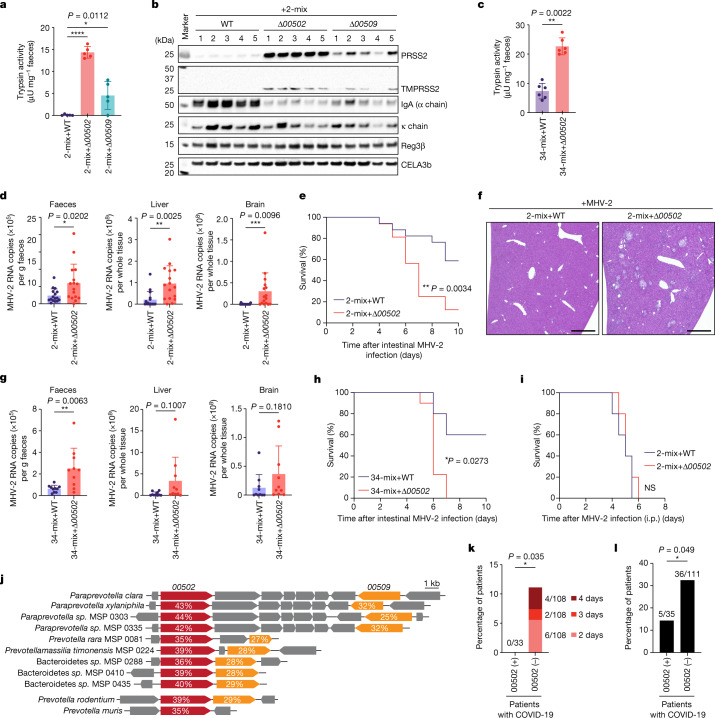
*Paraprevotella*-mediated degradation of trypsin modulates colonic homeostasis. **a**–**c**, GF mice were colonized with the indicated *P. clara* strains together with the 2-mix (**a**,**b**; *n* = 5 mice per group) or the 34-mix (**c**; *n* = 6 mice per group) for 14 days. Faecal trypsin activity (**a**,**c**) and the amount of indicated proteins (**b**; determined by western blotting) are shown. **d**–**f**, The viral RNA levels in the faeces or the indicated tissues (**d**), survival curve (**e**) and representative images of haematoxylin and eosin (H&E) staining of liver sections (**f**) of GF+2-mix+WT or GF+2-mix+*Δ00502* mice infected with MHV-2 (intragastric inoculation). Among the 32 (GF+2-mix+WT group) and 33 (GF+2-mix+*Δ00502* group) infected mice, 16 mice from each group were euthanized on day 5 for tissue viral RNA analysis (**d**) and the rest of the mice were followed for survival analysis (**e**). **g**,**h**, Viral RNA levels (**g**) and survival curve (**h**) of GF+34-mix+WT or GF+34-mix+*Δ00502* mice after intragastric inoculation with MHV-2. *n* = 15 mice per group (10 mice were euthanized on day 5 for tissue viral RNA analysis and the rest of the mice were followed for survival analysis). **i**, Survival curve of GF+2-mix+WT or GF+2-mix+*Δ00502* mice intraperitoneally injected with MHV-2. *n* = 5 mice per group. **j**, Genome neighbourhood of the homologues of the *P. clara 00502*-*00509* locus in human and mouse (*P. rodentium* and *P. muris*) gut microorganisms. The percentage amino acid identity with *P. clara* 00502 and 00509 is shown. **k**,**l**, The frequency of patients with COVID-19 experiencing more than 1 day with more than 2 diarrhoeal episodes per day (**k**) or requiring oxygen inhalation therapy (**l**), stratified by the presence (*00502* (+)) or absence (*00502* (−)) of *00502* homologue genes in the faecal metagenome. For **a**, **c**, **d** and **g**, data are mean ± s.d. Each dot represents one mouse. Statistical analysis was performed using one-way ANOVA with Tukey’s test (**a**), two-sided Mann–Whitney *U*-tests with Welch’s correction (nonparametric) (**c**, **d** and **g**), log-rank (Mantel–Cox) tests (**e**, **h** and **i**) and one-sided Fisher’s tests (**k** and **l**); ****P < 0.0001, ***P < 0.001, **P < 0.01, *P < 0.05. For **f**, scale bar, 500 μm. For **b**, images from one experiment, including all of the mice used in **a**, are shown. Blot source data are provided in Supplementary Fig. 1. Source data

**Extended Data Fig. 9:**
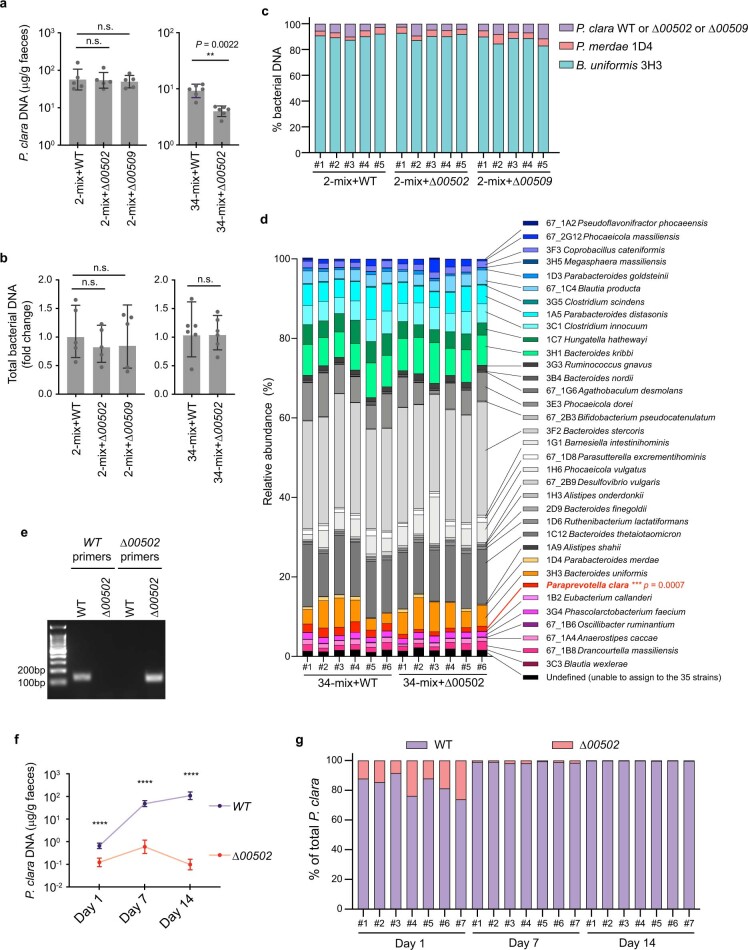
Trypsin degradation confers *P. clara* a fitness advantage under competitive conditions. **a**–**d**, Germ-free (GF) mice were colonized with wild type (WT), *Δ00502* or *Δ00509 P. clara* strains together with the 2-mix (*B. uniformis* 3H3 and *P. merdae* 1D4) (**a** & **b**, **left panels**, **c**), or colonized with WT or *Δ00502 P. clara* together with the 34-mix (**a** & **b**, **right panels**, **d**) for 14 days. *n* = 5 and 6 mice per group, respectively. Faecal *P. clara* DNA levels were determined by qPCR from a standard curve generated from serial dilutions of *P. clara* genome DNA (**a**). Fold change of total faecal bacterial DNA (relative to the average of the 2-mix+WT group and that of the 34-mix+WT group, respectively) was determined by a universal bacterial 16S rRNA gene primer pair (**b**). Faecal DNA of the 3 individual species was quantified by qPCR and their relative abundance was shown as percentage values (DNA of individual strain/total DNA of the 3 strains) (**c**). Relative abundance of the 35 individual bacterial species was analysed by 16s rRNA sequencing (**d**). **e**, Validation of the primers specifically amplifying genomic fragments from WT or *Δ00502 P. clara* strains for quantifying their abundance in (**f**, **g**). **f**, **g**, WT and *Δ00502 P. clara* strains were co-administered together with the 2-mix to GF mice. *n* = 7 mice. At the indicated days faecal DNA from each *P. clara* strain was quantified by qPCR. Both the absolute quantities (**f**) and the relative abundance (percentage of total *P. clara* DNA) (**g**) are shown. **a**, **b**, **f**, Data shown as mean ± s.d. **** *p* < 0.0001; ** *p* < 0.01; n.s., not significant. One-way ANOVA with Tukey’s test (**a** & **b, left panels**), two-sided Mann-Whitney test with Welch’s correction (nonparametric) (**a** & **b, right panels**), and two-sided multiple unpaired t tests (not corrected for multiple comparisons) (**f**). Each dot represents one mouse (**a**, **b**). **d**, Two-sided multiple unpaired t tests (corrected for multiple comparisons using the Sidak-Bonferroni method); *** adjusted *p* value < 0.001. All primers used for faecal bacterial DNA quantification are listed in Supplementary Table 5. **e**, An image from one experiment is shown. See Supplementary Figure 1 for gel source data. Source data

To confirm the contribution of 00502 and 00509 to trypsin degradation in vivo, we inoculated GF mice with the wild-type (WT), *Δ00502* or *Δ00509 P. clara* JCM14859 strain together with two trypsin non-degrading strains (2-mix; Fig. 2g) (notably, *P. clara* was unable to monocolonize mice). *P. clara* strains equally colonized the mouse intestine in combination with the 2-mix (Extended Data Fig. 9a–c). Consistent with our in vitro findings, mice colonized with *Δ00502 P. clara* retained high faecal trypsin levels, whereas mice colonized with *Δ00509 P. clara* showed a partial reduction in trypsin (Fig. 4a,b). Even in the presence of a more complex microbiota community (34-mix, see Fig. 2h), WT *P. clara* reduced faecal trypsin activity, whereas *Δ00502 P. clara* did not do so (Fig. 4c). Notably, under this relatively competitive condition, although the overall bacterial load or composition of the 34-mix strains showed little difference, WT *P. clara* colonized more abundantly than *Δ00502 P. clara* (Extended Data Fig. 9a,b,d). Moreover, *w*hen the two *P. clara* (WT and *Δ00502*) strains were co-administered to GF+2-mix mice, the WT strain colonized more effectively and eventually outcompeted the *Δ00502* strain (Extended Data Fig. 9e–g). These data suggest that 00502 has an essential role in facilitating trypsin degradation in vivo, and that the ability of trypsin degradation confers the bacterium with a colonization advantage under competitive conditions.

**Extended Data Fig. 10:**
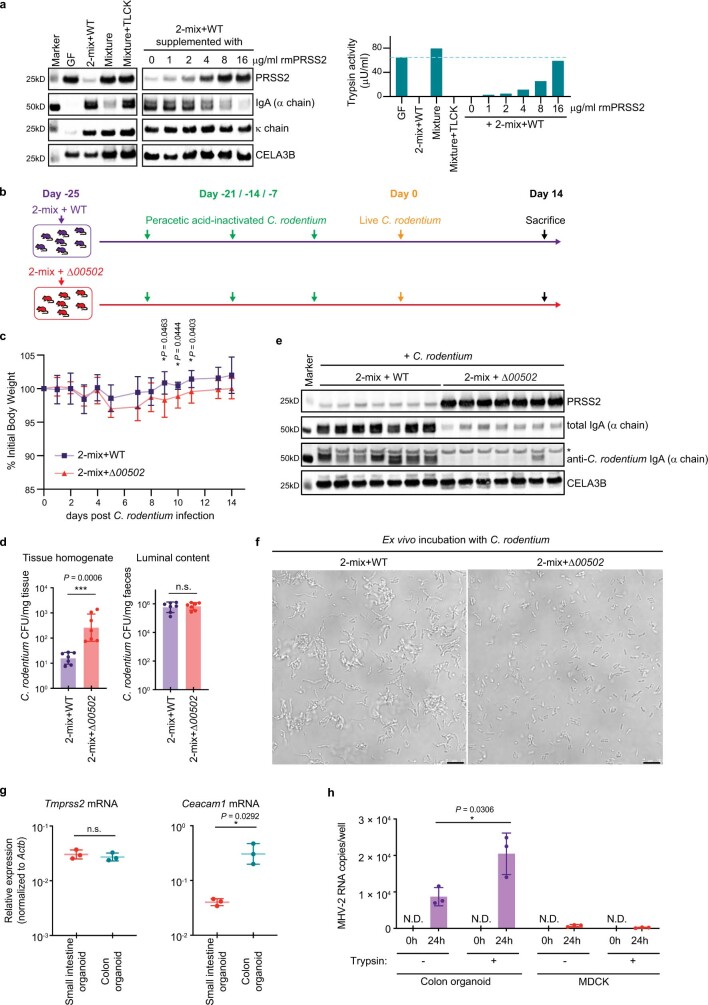
Low trypsin levels enhanced the effectiveness of oral vaccines against *Citrobacter rodentium in vivo* and reduced MHV-2 infection in mouse intestinal organoids. **a**, Ex vivo degradation of IgA heavy chain by trypsin: faeces from the 2-mix+WT *P. clara*-colonized mice (2-mix+WT) and germ-free mice (GF) were diluted and filtered, incubated alone, mixed together (Mixture), or mixed in the presence of trypsin-specific inhibitor TLCK (Mixture+TLCK). Alternatively, faeces from the 2-mix+WT *P. clara*-colonized mice (2-mix+WT) were incubated with the indicated concentrations of recombinant mouse trypsin (rmPRSS2). After incubation at 37 °C for 24 h the indicated proteins were analysed by Western Blot (**left panel**, anti-mouse PRSS2 antibody was used to detect both faecal and recombinant mouse PRSS2). **Right panel**: trypsin activity of the loaded samples (**left panel**). **b**, Schematic of the experimental setup for *C. rodentium* vaccination and infection (**c**–**f**). GF mice were inoculated with WT or *Δ00502 P. clara* JCM14859 strains (together with the 2-mix), orally vaccinated with peracetic acid-inactivated *C. rodentium* once per week for three weeks, followed by *C. rodentium* infection via oral gavage. **c**, Changes in body weight of mice following *C. rodentium* infection. **d**, Caecal patches and luminal contents were collected on day 14 post infection and analysed for *C. rodentium* CFU. **e**, Western Blot analysis for the indicated proteins in the caecal luminal contents following *C. rodentium* vaccination and infection. * non-specific band. See Materials & Methods for detection of total and *C. rodentium*-specific IgA. **f**, Agglutination effect of the filtered caecal suspension from 2-mix+WT *P. clara*- and 2-mix+*Δ00502 P. clara*-colonized mice (following *C. rodentium* vaccination and infection), as demonstrated by incubation with an *in vitro* culture of live *C. rodentium*. **g**, Relative expression of transmembrane protease, serine 2 (TMPRSS2) and CEA cell adhesion molecule 1 (CEACAM1) in the organoids derived from mouse small intestine and colon was determined by RT-qPCR using β-Actin (ACTB) as the reference gene. **h**, Colon organoids were infected with MHV-2 at MOI (multiplicity of infection) = 1 in the presence or absence of bovine trypsin for 2 h and washed with DMEM/F12 medium to remove uninfected virus. The viral RNA was quantified by RT-qPCR at 24 hrs post infection. MDCK cell line expressing the canine CEACAM1 with low homology to rodent CEACAM1 was used as the negative control. **c**, **d**, *n* = 7 mice per group. Data shown as mean ± s.d. (**c**) and geometric mean ± geometric s.d. (**d**); *** *p* < 0.001; * *p* < 0.05; n.s., not significant. Two-sided multiple unpaired t-tests (not corrected for multiple comparisons) (**c**) and two-sided Mann-Whitney test with Welch’s correction (nonparametric) (**d**). Each dot represents one mouse (**d**). **g**, **h**, *n* = 3 wells of cells per group. Each dot represents one well of cells. Data shown as mean ± s.d. * *p* < 0.05; n.s., not significant. Two-sided unpaired t test (parametric). N. D., not detected (**h**). Scale bar: 10 μm **(f**). **a, f**, Representative images from two experiments with similar results (**f**), or images from one experiment (**a**) are shown. **e**, Images from one experiment including all the mice used in panel **c** are shown. See Supplementary Figure 1 for blot source data. Source data

We next addressed the relevance of trypsin activity regulation in vivo. We examined its effects on immune molecules and found that mice colonized with WT *P. clara* had considerably higher levels of faecal IgA heavy chain (α chain) compared with mice colonized with *Δ00502* or *Δ00509 P. clara*, whereas the κ light chain and the antimicrobial peptide Reg3β showed little difference (Fig. 4b). Ex vivo incubation of faeces from GF+2-mix+WT *P. clara* mice (containing high IgA) with faeces from GF mice (containing high trypsin), or with recombinant trypsin, confirmed that the α chain is indeed trypsin sensitive (Extended Data Fig. 10a). These data suggest that *P. clara* colonization protects IgA, particularly the heavy chain, from proteolytic cleavage by trypsin in vivo.

Reasoning that *P. clara*-mediated trypsin degradation and the consequent protection of IgA might enhance the effectiveness of oral vaccines against enteropathogens, we used a vaccination model with *C. rodentium*. GF+2-mix+WT *P. clara* and GF+2-mix+*Δ00502 P. clara* mice were orally vaccinated with peracetic acid-inactivated *C. rodentium*^30^ and then infected with live *C. rodentium* (Extended Data Fig. 10b). Compared with *Δ00502 P. clara*-colonized mice, WT *P. clara*-colonized mice showed less reduction in body weight (Extended Data Fig. 10c), lower *C. rodentium* invasion into the caecal tissue (Extended Data Fig. 10d) and markedly higher levels of total IgA and *C. rodentium*-specific IgA in the caecum (Extended Data Fig. 10e). Caecal suspension from WT *P. clara*-colonized mice effectively formed agglutinations with in vitro cultured live *C. rodentium* (Extended Data Fig. 10f). These data suggest that *P. clara* colonization and IgA protection enable more effective responses to previously encountered enteropathogens.

## *P. clara* reduced MHV-2 spread

Trypsin and trypsin-like proteases, such as transmembrane protease serine 2 (TMPRSS2), are known to be involved in the proteolytic activation of the spike protein of coronaviruses^4,5,31–34^. TMPRSS2 is expressed on lung and gut epithelial cells as a transmembrane protein but can undergo autocleavage to release its protease domain^35^. Interestingly, we found that colonization with WT *P. clara* also reduced TMPRSS2 content in the faeces, suggesting that *P. clara* has a similar effect on the released active form of TMPRSS2 in vivo (Fig. 4b). To test the possibility that *P. clara* might inhibit intestinal infection of coronavirus through degradation of trypsin and TMPRSS2, we used murine hepatitis virus-2 (MHV-2), a mouse-tropic coronavirus that requires trypsin or TMPRSS2 to facilitate cleavage of S protein and fusion with cells^4,5^, like SARS-CoV and SARS-CoV-2^32–34^. To confirm that the mouse intestine is susceptible to MHV-2 infection, we generated organoids derived from the mouse intestinal epithelium. We detected expression of CEACAM1, the MHV-2 receptor^4^ and TMPRSS2 in the organoids (Extended Data Fig. 10g). Consistently, colonic organoid cells were permissive to MHV-2 infection, which was further enhanced by the presence of trypsin (Extended Data Fig. 10h). We next examined the effect of differential trypsin levels on intestinal MHV-2 infection in vivo. GF+2-mix+WT *P. clara* and GF+2-mix+*Δ00502 P. clara* mice were infected with MHV-2 through intragastric gavage. Mice colonized with WT *P. clara* showed reduced viral copy numbers in the faeces (day 1), liver and brain (days 4–5) (Fig. 4d) and a prolonged survival (Fig. 4e). MHV-2-induced necrotic liver pathology was less severe in mice colonized with WT *P. clara* (Fig. 4f). Similar observations were made in the context of a complex microbiota, that is, GF+34-mix+WT *P. clara* mice tended to be more resistant to MHV-2 infection compared with GF+34-mix+*Δ00502 P. clara* mice (Fig. 4g,h). Notably, when MHV-2 was applied through the intraperitoneal route, there was no difference in survival between WT *P. clara*-colonized and *Δ00502 P. clara*-colonized groups (Fig. 4i). Although further studies are required, these data suggest that *P. clara 00502* gene carriage and consequent protease degradation provide protective benefits to the host against MHV-2 infection through the intestinal route.

## *00502* homologues in the human microbiome

**Extended Data Fig. 11:**
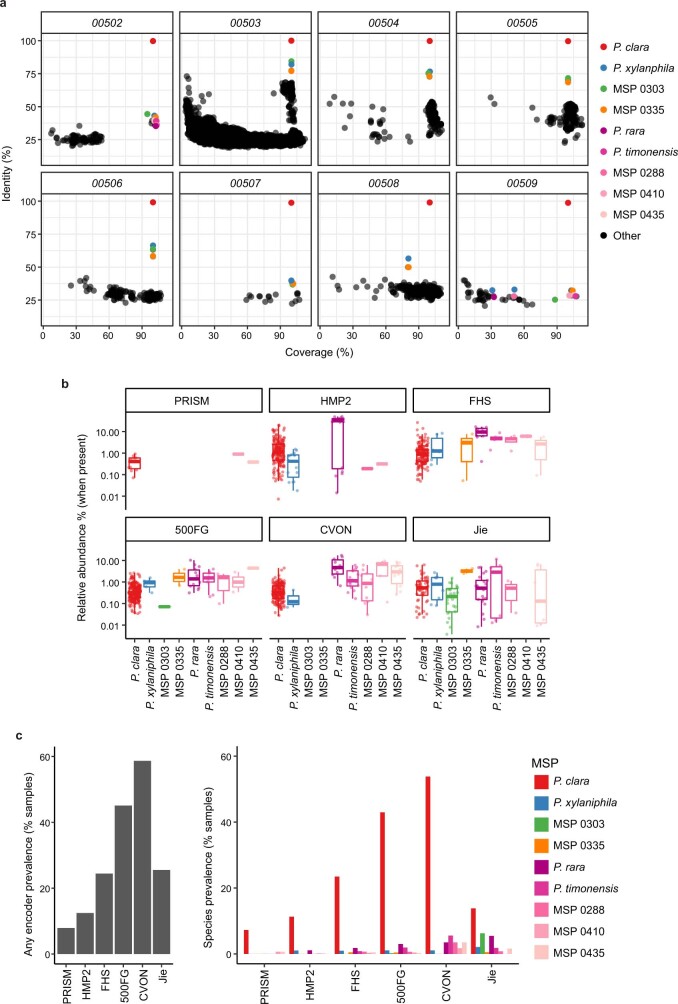
Detection of *00502*-carrying species in human and mouse microbiome. **a**, Computational mining for genes homologous to *P. clara 00502*-*00509* and encoding species. Results of homology search with USEARCH ublast (protein level) against a non-redundant gut microbiome gene catalogue with 5,929,528 genes, constrained to hits with minimum e-value of 0.1. Two metagenomic species (MSPs) annotated to *Paraprevotella* genus (MSP 0303 and MSP 0335) encoded all or almost all homologues to *P. clara* genes *00502-00509*. 5 MSPs annotated to *Bacteroidetes* (MSP 0081, MSP 0224, MSP 0288, MSP 0410 and MSP 0435) encoded homologues to genes *00502* and *00509* but lacked homologues to genes *00503*-*00508*. To arrive at these additional MSPs, we interrogated homology hits that showed levels of amino acid identity and coverage similar to that between *P. clara* and *P. xylanphila* homologues (see Methods) and were encoded by the same MSP. **b**, **c**, Relative abundance (**b**) and prevalence (**c**) of the 9 identified human *00502*-carrying species across 3372 *de novo* assembled human gut metagenomes from USA [PRISM (n = 152), HMP2 (n = 1462), FHS (n = 618)], Netherlands [500FG (n = 468), CVON (n = 288)] and China [Jie (n = 384)]. In **b**, thick horizontal lines indicate the median; box boundaries indicate interquartile range (IQR); whiskers represent values within 1.5 x IQR of the first and third quartiles. Source data

**Extended Data Fig. 12:**
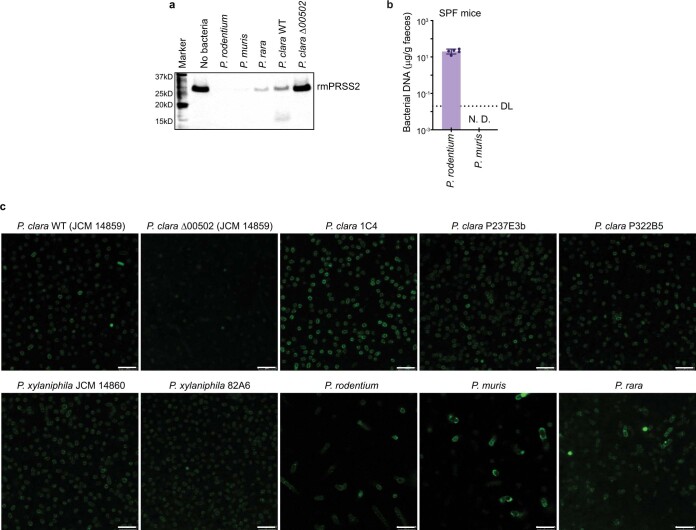
Trypsin degradation by *00502*-carrying species. **a**, Degradation of recombinant mouse trypsin (rmPRSS2) following *in vitro* incubation with the indicated bacterial strains. **b**, Quantification of faecal DNA from *Prevotella rodentium* and *Prevotella muris* in SPF mice reared at RIKEN’s facility by RT-qPCR. **c**, Alexa Fluor 488-labelled rmPRSS2 (green) was incubated with the indicated strains, and association of rmPRSS2 with the bacterial surface was examined by confocal microscopy. Scale bar: 5 μm (**c**). **b**, Data shown as geometric mean ± geometric s.d. *n* = 5 mice. DL, detection limit. N. D., not detected. **a**, **c**, A representative image from two independent experiments with similar results (**a**), or images from one experiment (**c**) are shown. See Supplementary Figure 1 for blot source data. Source data

We analysed the abundance and prevalence of *00502* and *00509* homologue genes by mining a de novo assembled human gut microbiome gene catalogue from 6 geographically diverse cohorts consisting of about 6 million non-redundant complete genes^36^. We first detected *P. clara*, *P. xylaniphila* and two additional metagenomic species (MSP0303 and MSP0335) that carry a conserved gene cluster with *00502*-*00509* homologues and potentially fall within the genus *Paraprevotella* (Fig. 4j and Extended Data Fig. 11a). We identified five additional Bacteroidetes metagenomic species (MSP0081 (*Prevotella rara*^37^), MSP0224 (*Prevotellamassilia timonensis*^38^), MSP0288, MSP0410 and MSP0435) that have *00502* and *00509* homologues only (Fig. 4j). These *00502*- and *00509*-carrying species showed, on average, a relative abundance of up to 9% (Extended Data Fig. 11b). Their prevalence varied greatly across the different cohorts, with *P. clara* being the most prevalent *00502* encoder (Extended Data Fig. 11c). We also mined a publicly available mouse metagenomic database and found *00502* homologues in the genomes of *Prevotella rodentium* and *Prevotella muris*^39^ (Fig. 4j). We obtained isolates of *P. rara*, *P. rodentium* and *P. muris*, and confirmed that all three isolates could facilitate trypsin degradation (Extended Data Fig. 12a). Thus, the presence of *00502* correlated well with the ability of a species to degrade trypsin. *P. rodentium* was detected in the faeces of the SPF mice reared in our facility (Extended Data Fig. 12b), possibly contributing to the low trypsin levels in these mice (Fig. 1g). All of the trypsin-degrading strains recruited fluorescently labelled trypsin to the surface (Extended Data Fig. 12c). The similarity of the predicted structures of all *00502* homologues suggests a common mechanism used by these species (Extended Data Fig. 8b–f).

## *00502* homologues and COVID-19 diarrhoea

Finally, we recruited 146 individuals who were diagnosed with COVID-19 and hospitalized at the Keio University hospital. Faecal samples were collected from the participants after discharge from the hospital and were processed for metagenome sequencing. We examined the association between the carriage of *00502* homologue genes in the gut microbiome and the disease severity and diarrhoea frequency (information of diarrhoea incidence along with the Bristol stool form scale (BSFS) during hospital care was available for 141 cases from medical records) (Supplementary Table 4). A total of 62 (44%) out of the 141 participants experienced diarrhoea (BSFS 5–7) during hospitalization. We found that the incidence of severe diarrhoeal episodes (more than twice per day lasting for more than 1 day) was significantly more frequent in participants who were negative for *00502* homologues (*P* = 0.035, one-sided Fisher’s test) (Fig. 4k). Moreover, the absence of *00502* homologues in the gut microbiome was significantly associated with a higher rate of oxygen inhalation (*P* = 0.049, one-sided Fisher’s test) (Fig. 4l). Although further studies are required, these results are consistent with our hypothesis that trypsin-degrading commensal colonization may provide protective benefits against SARS-CoV-2 infection.

## Discussion

Here we identified gut commensals that effectively degrade trypsin in the large intestine. Mechanistically, the degradation is mediated by the T9SS-dependent, polysaccharide-binding outer membrane proteins 00502 and 00509. We show that 00502 is absolutely essential for trypsin recruitment and autodegradation by *Paraprevotella*, and that the autodegradation is possibly facilitated by 00502 oligomerization (Extended Data Fig. 8a). Degradation of trypsin probably increases the fitness of trypsin-degrading species in a competitive environment. Moreover, trypsin affects intestinal IgA levels and responses to previously encountered enteropathogens. Carriage of the *00502* gene was associated with resistance to MHV-2 infection in mice and reduced diarrhoea severity during COVID-19 in humans, suggesting that 00502-mediated trypsin degradation potentially affects host sensitivity to intestinal viral infections. There are a number of limitations to our metagenome analysis of our COVID-19 cohort. In particular, owing to the small number of participants, the data were unadjusted for known confounders such as age, sex and comorbidities. The causal relationship between trypsin degradation and the protection against SARS-CoV2 infection needs to be further validated by larger cohorts and additional animal models. Nevertheless, our study provides valuable insights into the mechanisms and physiological implications of microbiota-mediated protease regulation. Moving forwards, we could take advantage of the unique trypsin-degrading ability of the identified bacteria and molecules to treat or prevent infectious diseases.

## Methods

### Mice

C57BL/6N mice maintained under SPF or GF conditions were purchased from Sankyo Laboratories Japan, SLC Japan, Charles River Japan or CLEA Japan. Gnotobiotic mice were maintained within the gnotobiotic facility of RIKEN IMS. SPF and GF WT male and female mice (aged 8–12 weeks) were used in this study. Sex-matched littermates were used in all of the experiments. All of the animals were maintained under a 12 h–12 h light–dark cycle and received gamma-irradiated (50 kGy) pellet food (CMF, Oriental Yeast). A temperature of 20–24 °C and a humidity of 40–60% were used for the housing conditions. All of the animal experiments were approved by the Animal Care and Use Committee of RIKEN Yokohama Institute.

### Collection of human faecal samples for trypsin-activity assays and for colonization of GF mice with human microbiota

Human faecal samples were collected at the RIKEN Institute (code H30-4, for patients with inflammatory bowel disease) and Keio University (code 20150075, for healthy donors) according to the study protocols approved by the institutional review boards. Informed consent was obtained from each participant.

### Bacterial strains

*P. clara* JCM14859, *P. xylaniphila* JCM14860, *Prevotella copri* JCM13464, *Prevotella denticola* JCM13449*, Prevotella stercorea* JCM13469 and *Prevotella oulorum* JCM14966 were acquired from the Japan Collection of Microorganisms (JCM)*. P. clara* P237E3b and P322B5 strains were derived from Vedanta Biosciences. *P. xylaniphila* 82A6 was a strain isolated at the Honda laboratory^40^. *P. rara* (DSM 105141), *P. rodentium* (DSM 105243) and *P. muris* (DSM 103722) were obtained from the DSMZ-German collection of Microorganisms and Cell Cultures. Bacterial strains are available under a contract with material transfer agreement with RIKEN.

### Proteome analysis of caecal contents

Proteins in caecal contents were extracted by pipetting and inverting in TBST with protease inhibitors. After centrifugation at 15,000*g* for 20 min at 4 °C to remove insoluble matter, the supernatant was transferred to a new tube, 25% trichloroacetic acid was added (final concentration 12.5% (v/v)) and incubated for 1 h at 4 °C. After removing the supernatant by centrifugation at 15,000*g* for 15 min at 4 °C, the precipitate was washed twice with acetone and dried with the lid open. The dried sample was redissolved in 0.5% sodium dodecanoate and 100 mM Tris-HCl, pH 8.5 using a water-bath-type sonicator (Bioruptor UCD-200, SonicBio). The redissolved sample was assayed for protein concentration using the BCA assay and the protein concentration was adjusted to 1 μg μl^−1^. Pretreatment for shotgun proteome analysis was performed as previously reported^14^.

Peptides were directly injected onto a 75 μm × 15 cm, PicoFrit emitter (New Objective) packed in house with 2.7 μm core shell C18 particles (CAPCELL CORE MP 2.7 μm, 160 Å material; Osaka Soda) and then separated with a 180 min gradient at a flow rate of 300 nl min^−1^ using the Eksigent Ekspert NanoLC 400 HPLC system (Sciex). Peptides eluting from the column were analysed using the TripleTOF 5600+ mass spectrometer (Sciex) for both shotgun-MS and sequential window acquisition of all theoretical mass spectra (SWATH)-MS analyses. For shotgun-MS-based experiments, MS1 spectra were collected in the range of 400–1,000 *m*/*z* for 250 ms. The top 25 precursor ions with charge states of 2^+^ to 5^+^ that exceeded 150 counts per s were selected for fragmentation with a rolling collision energy, and MS2 spectra were collected in the range of 100–1,500 *m*/*z* for 100 ms. Dynamic exclusion time was set to 24 s. For SWATH-MS based experiments, the mass spectrometer was operated in a consecutive data-independent acquisition mode with 12 *m*/*z* increments in precursor isolation window. Using an isolation width of 13 *m*/*z* (1 *m*/*z* for the window overlap), a set of 50 windows was constructed covering the precursor mass range of 400–1,000 *m*/*z*. SWATH MS2 spectra were in the range of 100–1,500 *m*/*z* for 60 ms per MS2 experiment. Precursor ions were fragmented for each MS2 experiment using rolling collision energy.

All shotgun-MS files were searched against the mouse UniProt reference proteome (UP000000589; reviewed, canonical) using ProteinPilot software v.4.5 with the Paragon algorithm (Sciex) for protein identification. The protein confidence threshold was a ProteinPilot unused score of 1.3 with at least one peptide with 95% confidence. The global false-discovery rate for both peptides and proteins was lower than 1% in this study. The identified proteins were quantified from SWATH-MS data using PeakView v.2.2 (Sciex).

### Proteome analysis of *P. clara* culture supernatant

Trichloroacetic acid (25%; final concentration 12.5% (v/v)) was added to the *P. clara* culture supernatant and incubated for 1 h at 4 °C. After removing the supernatant by centrifugation at 15,000*g* for 15 min at 4 °C, the precipitate was washed twice with acetone and dried with the lid open. The dried sample was redissolved in 0.5% sodium dodecanoate and 100 mM Tris-HCl, pH 8.5 by using a water-bath-type sonicator (Bioruptor UCD-200). The redissolved sample was assayed for protein concentration using the BCA assay, and the protein concentration was adjusted to 1 μg μl^−1^. The pretreatment for shotgun proteome analysis was performed as previously reported^14^. Peptides were directly injected onto a 75 μm × 20 cm PicoFrit emitter packed in house with 2.7 μm core shell C18 particles at 50 °C and then separated with an 80 min gradient at a flow rate of 100 nl min^−1^ using the UltiMate 3000 RSLCnano LC system (Thermo Fisher Scientific). Peptides eluting from the column were analysed using the Q Exactive HF-X (Thermo Fisher Scientific) system for overlapping window DIA-MS^14,41^. MS1 spectra were collected in the range of 495–785 *m*/*z* at 30,000 resolution to set an automatic gain control (AGC) target of 3 × 10^6^ and a maximum injection time of 55. MS2 spectra were collected in the range of more than 200 *m*/*z* at 30,000 resolution to set an AGC target of 3 × 10^6^, maximum injection time of ‘auto’ and stepped normalized collision energy of 22, 26 and 30 %. An isolation width for MS2 was set to 4 *m*/*z* and overlapping window patterns in 500–780 *m*/*z* were used window placements optimized by Skyline^42^.

MS files were searched against a *P. clara* spectral library using Scaffold DIA (Proteome Software). The spectral library was generated from *P. clara* protein sequence databases by Prosit^43,44^. The *P. clara* protein sequence database was independently created by metagenomic analysis. The Scaffold DIA search parameters were as follows: experimental data search enzyme, trypsin; maximum missed cleavage sites, 1; precursor mass tolerance, 8 ppm; fragment mass tolerance, 8 ppm; static modification, cysteine carbamidomethylation. The protein identification threshold was set with both peptide and protein false-discovery rates of less than 1%. Peptide quantification was calculated using the EncyclopeDIA algorithm^45^ in Scaffold DIA. For each peptide, the four highest-quality fragment ions were selected for quantification. Protein quantification was estimated from the summed peptide quantification.

### Peptidome analysis

To the caecal contents, acetonitrile containing 0.1% TFA was added and dried in a centrifugal evaporator. Acetone was added to the dried sample and lipid-soluble small molecules were extracted with a water-bath-type sonicator, followed by centrifugation at 15,000*g* for 15 min at 4 °C. After the supernatant was removed, 70% acetonitrile-12 mM HCl^46^ was added to the precipitate and the peptide was redissolved by a water-bath-type sonicator, followed by centrifugation at 15,000*g* for 15 min at 4 °C. The supernatant was transferred to a new tube and dried in a centrifugal evaporator. The dried sample was redissolved in 100 mM Tris-HCl and protease inhibitors, and treated with 10 mM dithiothreitol at 50 °C for 30 min. Subsequently, the sample was alkylated with 30 mM iodoacetamide in the dark at room temperature for 30 min and acidified with 0.5% trifluoroacetic acid (final concentration). The acidified sample was desalted by Monospin C18 (GL Sciences).

Peptides were directly injected onto a 75 μm × 25 cm PicoFrit emitter (New Objective) packed in-house with C18 core-shell particles (CAPCELL CORE MP 2.7 μm, 160 Å material; Osaka Soda) at 50 °C and then separated with a 90 min gradient at a flow rate of 100 nl min^−1^ using an UltiMate 3000 RSLCnano LC system (Thermo Fisher Scientific). Peptides eluting from the column were analysed using the Q Exactive HF-X (Thermo Fisher Scientific) for DDA-MS. MS1 spectra were collected in the range of 380 to 1,500 *m*/*z* with 120,000 resolution to hit an AGC target of 3 × 10^6^. The 30 most intense ions with charge states of 2^+^ to 8^+^ that exceeded 4.4 × 10^3^ were fragmented in data-dependent mode by collision-induced dissociation with stepped normalized collision energy of 21%, 25% and 29%, and tandem mass spectra were acquired on the Orbitrap mass analyser with a mass resolution of 30,000 at 200 *m*/*z* to set an AGC target of 2 × 10^5^.

MS files were searched against the mouse UniProt reference proteome (UP000000589; reviewed, canonical) by PEAKS Studio. The search parameters were as follows: precursor mass tolerance, 8 ppm; fragment ion mass tolerance, 0.01 Da; enzyme, no enzyme; fixed modifications, carbamidomethylation; variable modifications, oxidation (M). The peptide identification was filtered to a peptide false-discovery rate of less than 1%.

### In-gel digestion and LC–MS/MS analysis

The protein bands were excised, and in-gel digestion was performed as previously described^47^. The digested peptides were directly injected onto a 75 μm × 12 cm PicoFrit emitter (New Objective) at 40 °C and then separated with a 30 min gradient at a flow rate of 200 nl min^−1^ using the UltiMate 3000 RSLCnano LC system (Thermo Fisher Scientific). Peptides eluted from the column were analysed on the Q Exactive HF-X (Thermo Fisher Scientific) system for DDA-MS. MS1 spectra were collected in the range of 380 to 1,240 *m*/*z* with 120,000 resolution to hit an AGC target of 3 × 10^6^. The 20 most intense ions with charge states 2^+^ to 5^+^ were data-dependently dissociated by collision-induced dissociation with step-normalized collision energies of 22%, 26% and 30%, and tandem mass spectra were acquired on the Orbitrap mass analyser with 30,000 resolution to set an AGC target of 1 × 10^5^.

MS files were searched against the *P. clara* protein sequence database with human PRSS2 sequence (UniProt: P07478) using PEAKS Studio. The search parameters were as follows: precursor mass tolerance, 8 ppm; fragment ion mass tolerance, 0.01 Da; enzyme, trypsin; variable modifications, oxidation (M). Peptide and protein identifications were filtered so that both peptide and protein false discovery rates were less than 1%.

### Western blot analysis

Mouse caecal and faecal samples were suspended and diluted 50-fold in PBS supplemented with a protease inhibitor cocktail (Roche cOmplete, Mini, EDTA-free). Resuspended samples were centrifuged at 4 °C, 15,000*g* for 10 min, and the supernatant was collected for western blotting. Mouse pancreatic tissues were snap-frozen in liquid nitrogen and the proteins were extracted using TRIzol Reagent (Thermo Fisher Scientific), and the final protein concentration was adjusted to 4 μg μl^−1^. For SDS–PAGE and blotting, the Novex NuPAGE SDS–PAGE Gel system (Thermo Fisher Scientific) and iBlot 2 Dry Blotting System (Thermo Fisher Scientific) were used according to the manufacturer’s instructions. In some earlier experiments, SDS–PAGE and PVDF membrane (0.2 μm Transfer Membranes Immobilon-P^SQ^, Merck Millipore) transfer were performed according to the manufacturer’s (XV Pantera System (DRC)) instructions. iBind Western Systems (Thermo Fisher Scientific) were used for staining throughout the study. The antibodies used in this study are as follows: rabbit anti-mouse PRSS2 (Cosmo Bio, CPA, Japan, custom-made), rabbit anti-mouse HSP90 antibody (4877, C45G5, Cell Signaling Technology), rabbit anti-human PRSS2 (LS-B15726, LSBio), rabbit anti-human PRSS1 (LS-331381, LSBio), rabbit anti-mouse TMPRSS2 (LS-C373022, LSBio, raised against a sequence at the protease domain), rabbit anti-6-His (A190-214A, Bethyl laboratories, to probe His-tagged recombinant mouse PRSS2 (rmPRSS2) and human PRSS3 (rhPRSS3)), goat anti-mouse IgA alpha-chain (HRP) (ab97235, Abcam), rat anti-mouse kappa-chain (HRP) (ab99632, Abcam), rabbit anti-mouse CELA3b (OACD03205, Avivasysbio), anti-rabbit IgG (HRP-linked antibody) (7074, Cell Signaling Technology), rabbit anti-mouse Reg3β (51153-R005, Sino Biological). Rabbit anti-6-His antibodies (A190-214A, Bethyl laboratories) were used to probe rmPRSS2 throughout the study except for the experiment in Fig. 3j, for which rabbit anti-mouse PRSS2 (Cosmo Bio, CPA, custom-made) was used to differentiate rmPRSS2 from recombinant 00502 and 00509 (also His-tagged). For staining, a 1:400 dilution was used for all the primary antibodies and secondary antibodies. Chemi-Lumi One (nacalai tesque) was used for the chemiluminescence assays and the Molecular imager ChemiDoc XRS+ (BIO-RAD) or iBright FL1500 system was used for imaging. Full scans of all of the blots are provided in Supplementary Fig. 1.

### RT–qPCR

RNA from mouse pancreas, small intestine and colon organoids was extracted by TRIzol Reagent (Thermo Fisher Scientific). Extracted RNA was converted to cDNA using the ReverTra Ace qPCR RT Master Mix with gDNA Remover (TOYOBO). RT–qPCR analysis was conducted with the Thunderbird SYBR qPCR Mix (Toyobo) and Lightcycler480 v.1.5.1 (Roche) software and analysed using the ΔΔ*C*_t_ method or using a standard curve generated from serial dilutions of pooled cDNA (for *Tmprss2*, *Ceacam1* and *Actb*). *Gapdh* and *Actb* were used as the endogenous control. Primer sequences were as follows: *Gapdh* forward primer, 5′-GTCGTGGAGTCTACTGGTGTCTTC-3′; *Gapdh* reverse primer, 5′-GTCATATTTCTCGTGGTTCACACC-3′; *Prss2* forward primer, 5′-TGTGACCCTCAATGCCAGAG-3′; *Prss2* reverse primer, 5′-AGCACTGGGGCATCAACAC-3′; *Tmprss2* forward primer, 5′-AACGCAAGCCTCAACATCTG-3′; *Tmprss2* reverse primer, 5′-AACCTCCAAAGCAAGACAGC-3′; *Ceacam1* forward primer, 5′-GCCTGGCTTAGCAGTAGTGT-3′; *Ceacam1* reverse primer, 5′-CCAGGAGGCTAAAAGTGAGG-3′; *Actb* forward primer, 5′-TTGCTGACAGGATGCAGAAG-3′; *Actb* reverse primer, 5′-ATCCACATCTGCTGGAAGGTG-3′.

### Immunofluorescence

Mouse colon tissues (containing faecal pellet) were sampled and fixed with Cornoy solution (60% methanol, 30% chloroform, 10% glacial acetic acid) at 4 °C overnight. A tissue processor (Leica Microsystems) was used for paraffin embedding. Paraffin blocks were processed into thin sections (5.0 μm) using a microtome, followed by paraffin removal and immunostaining. The antibodies and stains used for immunofluorescence were as follows: rabbit anti-PRSS2 (LSBio, LS-C296077, 1:100), Alexa 488-labelled goat anti-rabbit IgG (Thermo Fisher Scientific, A11008, 1:1,000), 4′-6-diamidino-2-phenylindole (DAPI, DOJINDO), rhodamine-labelled UEA1 (Ulex Europaeus Agglutinin 1, Vector Laboratories). The Leica AF600 and confocal Leica TCS SP5 systems were used for immunofluorescence imaging.

### Trypsin-activity assay of mouse and human faecal samples

Mouse intestinal luminal contents or faecal samples were diluted 500-fold (w/v) in 0.9% NaCl solution. Human faecal samples were diluted 200-fold (w/v) in 0.9% NaCl solution. The diluted solutions were vortexed with a mini-shaker for 20 min at 2,000 rpm, homogenized by pipetting and centrifuged at 4 °C and 10,000*g* for 15 min. The supernatant was collected for trypsin-activity assay using the Trypsin Activity Assay Kit (Colorimetric) (ab102531) according to the manufacturer’s protocol. Absorbance at 405 nm was measured using the PerkinElmer 2030 Multilabel Reader in kinetic mode.

### Colonization of GF mice with human microbiota

Human faecal samples (preserved in 20% (v/v) glycerol) were transferred to an anaerobic chamber, thawed and sieved through 100 μm meshes, transferred into a GF isolator and introduced into GF mice by oral gavage (200 μl per mouse). For antibiotics treatment, 0.5 g l^−1^ ampicillin (nacalai tesque), 0.5 g l^−1^ metronidazole (nacalai tesque) and 1.0 g l^−1^ tylosin (Sigma-Aldrich) solutions were made using autoclaved tap water. Mice receiving oral gavage of the caecal contents from the donor-C-microbiota-colonized mouse were fed with antibiotic solutions for 12 days. Antibiotic solutions were replaced once per week.

### Isolation and identification of colonized species from mouse caecal contents

Mouse caecal contents were mixed with glycerol-containing (20%) PBS in an anaerobic chamber and stocked at −80 °C. An aliquot was diluted with TS broth (BD) in an anaerobic chamber and plated onto different agar plates: EG, ES, M10, NBGT, VS, TS (BD), BL (Eiken Chemical), BBE (Kyokuto Seiyaku), Oxoid CM0619 (Thermo Fisher Scientific), CM0619-supplemented SR0107 (Thermo Fisher Scientific), CM0619-supplemented SR0108 (Thermo Fisher Scientific), mGAM (NISSUI-Pharm) and Schaedler (BD). After incubation for 2 days, colonies with different appearances were transferred to new EG plates. Colonies were then incubated in EGEF liquid medium overnight, mixed with glycerol (final concentration 20% (v/v)) and stocked at −80 °C.

The formula of EG (Eggerth Gagnon) agar plates is as follows: protease peptone no. 3 (10.0 g), yeast extract (5.0 g), Na_2_HPO_4_ (4.0 g), glucose (1.5 g), soluble starch (0.5 g), l-cysteine HCl (0.5 g), l-cystine (0.2 g), Tween-80 (0.5 g), agar (4.8 g), horse meat extract (500 ml), water up to 1,000 ml + defibrinated horse blood (50 ml). EGEF medium was the same, except with no agar and defibrinated horse blood (50 ml) was replaced with Fildes solution (40 ml).

The bacterial DNA genome was extracted from the isolated strains using the same protocol as DNA isolation from faecal samples (below). 16S rRNA was amplified by PCR using the KOD plus Neo (TOYOBO) kit according to the manufacturer’s protocol. Sanger sequencing was performed by Eurofins. Sequences were blasted against NCBI database. Primers for Sanger sequencing were as follows: F27 primer, 5′-AGRGTTTGATYMTGGCTCAG-3′; R1492 primer, 5′-TACGGYTACCTTGTTACGACTT-3′.

### 16S rRNA sequencing

Frozen mouse faecal samples were thawed and 100 µl of the suspensions was mixed with 900 µl TE10 (10 mM Tris-HCl, 10 mM EDTA) buffer containing RNase A (final concentration 100 µg ml^−1^, Invitrogen) and lysozyme (final concentration 3.0 mg ml^−1^, Sigma-Aldrich). The suspension was incubated for 1 h at 37 °C with gentle mixing. Purified achromopeptidase (Wako) was added to a final concentration of 2,000 U ml^−1^ and the sample was further incubated for 30 min at 37 °C. Sodium dodecyl sulfate (final concentration 1%) and proteinase K (final concentration 1 mg ml^−1^, Nacalai) were then added to the suspension and the mixture was incubated for 1 h at 55 °C. High-molecular-mass DNA was extracted by phenol:chloroform:isoamyl alcohol (25:24:1), precipitated by isopropanol, washed with 70% ethanol and resuspended in 100 µl of TE. PCR was performed using Ex Taq (Takara) and the 27Fmod primer (5′-AATGATACGGCGACCACCGAGATCTACACXXXXXXXXACACTCTTTCCCTACACGACGCTCTTCCGATCTAGRGTTTGATYMTGGCTCAG-3′) and the 338R primer (5′-CAAGCAGAAGACGGCATACGAGATXXXXXXXXGTGACTGGAGTTCAGACGTGTGCTCTTCCGATCTTGCTGCCTCCCGTAGGAGT-3′) to the V1–V2 region of the 16S rRNA gene (where XXXXXXXX represents the Miseq (Illumina) Index sequence). The PCR product was purified with Agencourt AMPure XP (Beckman Coulter) according to the manufacturer’s protocol. The 16S rRNA library was created using the Kapa library quantification Kit (Kapa Biosystems) according to the manufacturer’s protocol. 16S rRNA sequencing was conducted using the standard protocol of MiSeq Reagent kit v.3. The obtained 16S rRNA sequencing data were analysed as previously described^48^. UCLUST (https://www.drive5.com/) was used to construct OTUs. Taxonomy was assigned to each OTU by searching against the National Center for Biotechnology Information (NCBI) using the GLSEARCH program.

### Gnotobiotic studies and quantification of faecal bacterial DNA

With the exception of *Phascolarctobacterium faecium* (3G4), isolated bacterial strains were incubated in EGEF in an anaerobic chamber at 37 °C for 1–2 days. *P. faecium* was incubated on Oxoid CM0619 agar plates supplemented with 80 mM succinic sodium for 2–3 days, and colonies were collected and resuspended in EGEF. Bacterial density was adjusted on the basis of optical density at 600 nm (OD_600_) values and mixtures of the cultured strains were administered into GF mice (150 µl per mouse, approximately 1–2 × 10^8^ colony-forming units (CFU) of total bacteria) by oral gavage. For quantification of faecal DNA of *P. clara*, *P. merdae*, *B. uniformis*, *P. rodentium* and *P. muris*, mouse faecal DNA was purified and qPCR was performed to amplify a sequence specific to respective bacterial 16S rRNA gene using the Thunderbird SYBR qPCR Mix (Toyobo) on the LightCycler 480 System (Roche). For quantification of faecal DNA of the WT or *Δ00502 P. clara*, qPCR was carried out to amplify a sequence specific to the *00502* gene (for the WT) or a sequence spanning the upstream and downstream fragment of the *00502* gene (for *Δ00502*). Standard curves were generated from serial dilutions of bacterial genomic DNA purified from in vitro bacterial cultures of the respective strains. For analyses of the total faecal bacterial DNA, a universal bacterial 16S rRNA gene primer pair was used^49^. A list of all of the primers used for faecal bacterial DNA quantification is provided in Supplementary Table 5.

### Bacterial whole-genome sequencing

Genomic DNA was extracted from the isolated bacteria including the *P. clara* 1C4 strain and sheared to yield DNA fragments. Bacterial genome sequencing was performed using the whole-genome shotgun strategy supported by the PacBio Sequel and Illumina MiSeq sequencing platforms. The TruSeq DNA PCR-Free kit was used to prepare the library of the Illumina Miseq 2 × 300 bp paired-end sequencing with target length of 550 bp, and the FASTX-toolkit (http://hannonlab.cshl.edu/fastx_toolkit) was used to trim and filter all of the MiSeq reads with a >20 quality value. The SMRTbell template prep kit 2.0 was used to generate the library of the PacBio Sequel sequencing with a target length of 10–15 kb without DNA shearing. Error correction of the trimmed reads was conducted by Canu (v.1.8) with additional options (corOutCoverage = 10,000, corMinCoverage = 0, corMhapSensitivity = high) after internal control removal and adapter trimming by Sequel. De novo hybrid assembly of the filter-passed MiSeq reads and the corrected Sequel reads was performed by Unicycler (v.0.4.8), including a check of overlapping and circularization, and a circular contig was generated. The Rapid Annotations based on Subsystem Technology (RAST) server and Prokka software tool were used for gene prediction and annotation of the generated contig. The default parameters were used for all software unless specified otherwise.

### *C. rodentium* vaccination and infection

GF mice were pre-inoculated with 200 μl of 2-mix (*B. uniformis* 3H3 and *P. merdae* 1D4) + WT *or Δ00502 P. clara* and maintained for 4 days. The mice were then orally administered peracetic-acid-inactivated *C. rodentium* (10^10^ per mouse) once per week for three weeks. After three weeks of immunization, the mice were infected with an overnight culture of *C. rodentium* (150 µl per mouse) by oral gavage and euthanized on day 14 after infection. Peracetic-acid-inactivated *C. rodentium* was generated as previously described^30^. In brief, overnight cultures of *C. rodentium* were collected by centrifugation (16,000*g*, 10 min) and resuspended at a density of 10^10^ per ml in sterile PBS. Peracetic acid (240990, Sigma-Aldrich) was added to the bacterial suspension (final concentration, 0.4%) and incubated for 1 h at room temperature. After washing three times with sterile PBS, the final pellet was resuspended at a final concentration of 10^11^ particles per ml in PBS and stored at 4 °C. The vaccine was tested before use by inoculating 100 µl of the inactivated vaccine into 200 ml LB medium and incubating overnight at 37 °C to ensure complete inactivation. For the CFU assay, caecal patches or caecal luminal contents were collected and homogenized in PBS, and serially diluted homogenates were plated on LB agar plates. CFUs were counted after overnight incubation at 37 °C under aerobic conditions. For ex vivo evaluation of *C. rodentium*-specific IgA, caecal contents were diluted fivefold (w/v) in LB medium, centrifuged and the supernatant was filtered with sterile filter units with PVDF membranes (0.22 µm pore size) before being mixed with equal volumes of an in vitro overnight *C. rodentium* culture. The mixture was incubated at room temperature with gentle shaking for 1 h, and the agglutination effect was examined using a confocal microscope (Leica TCS SP8). Alternatively, after incubation, the mixture was centrifuged, washed once with PBS and the bacterial pellets were lysed with 1% SDS solution (in 50 mM Tris-HCl buffer supplemented with 5 mM EDTA). The lysates were stained with goat anti-mouse IgA alpha-chain (HRP) antibodies (ab97235) by western blotting to evaluate the amount of *C. rodentium-*binding (*C. rodentium*-specific) IgA in the caecal contents.

### MHV-2 infection in vivo

MHV-2 was propagated in DBT cells as previously reported^4^. GF C57BL/6N male mice (aged 5 weeks) were obtained from CLEA Japan or Sankyo Labo Service and housed in separate stainless-steel isolators. GF mice were orally inoculated with 200 μl of 2-mix (*B. uniformis* 3H3 and *P. merdae* 1D4) + WT *P. clara* or 2-mix+*Δ00502 P. clara*, or 34-mix+WT *P. clara* or 34-mix+*Δ00502 P. clara*. Two weeks after inoculation, the mice were infected with 4.5 × 10^6^ plaque-forming units of MHV-2 through intragastric gavage using a long (4 cm) catheter, and survival was monitored daily for 10 days. To detect and quantify MHV-2, the livers and brains were collected at day 4 or day 5 after infection and homogenized in DNA/RNA shield (Zymo Research). Viral RNA was extracted using the Quick-RNA Viral Kit (Zymo Research) according to the manufacturer’s instructions, and cDNA was synthesized using ReverTra Ace (TOYOBO) and random primers (TOYOBO). qPCR was performed to amplify a fragment in the 5′ region of viral *ORF1a* (5′-AAGAGTGATTGGCGTCCGTAC-3′ and 5′-ATGGACACGTCACTGGCAGAG-3′) using the THUNDERBIRD SYBR qPCR Mix (TOYOBO) on a LightCycler 480 System (Roche). The quantity of MHV-2 was calculated on the basis of a standard curve generated using a plasmid with a predetermined copy number inserted with the cDNA of a 5′ region (175 bp) of viral *ORF1a*. For histological examination, the livers were collected at day 5 after infection and fixed with 4% paraformaldehyde overnight at 4 °C. H&E staining was performed at the Pathology Analysis Center, Central Institute for Experimental Animals (CIEA). In brief, fixed tissue was embedded in paraffin, serially sectioned at a thickness of 5 μm and stained with H&E. The images were captured with the BX-X800 microscope (Keyence).

### Organoid culture and MHV-2 infection

Mouse small intestine and colon organoids were established as previously described^50,51^. In brief, intestine tissues were opened longitudinally, washed with ice-cold PBS, cut into small pieces and subsequently treated with 5 mM EDTA on a rocking shaker for 30 min at 4 °C. After the supernatant was carefully removed, the remaining tissue was washed with PBS by pipetting up and down, followed by passed through 70 μm cell strainers, and centrifuged at 300*g* for 3 min. Isolated crypts were embedded in Matrigel (Corning) and cultured with organoid growth medium, as follows: Advanced DMEM/F-12 (Gibco) supplemented with 10 mM HEPES, 2 mM GlutaMAX, 100 U ml^−1^ penicillin, 100 μg ml^−1^ streptomycin, 20% Afamin/Wnt3a CM (MBL), 50 ng ml^−1^ mouse recombinant EGF (Gibco), 100 ng ml^−1^ mouse recombinant noggin (Peprotech), 1 μg ml^−1^ human recombinant R-spondin 1 (R&D Systems), 500 nM A 83-01 (Tocris), 1× N2 supplement (Gibco), 1× B-27 supplement (Gibco) and 1 mM *N*-acetyl-l-cysteine (Sigma-Aldrich). The organoids were passaged mechanically every 4–5 days.

Before MHV-2 infection, organoids and MDCK cells (ATCC, CCL-34, mycoplasma-free) were dissociated into single cells using TrypLE express. A total of 2 × 10^5^ cells was infected at a multiplicity of infection of 1 for 2 h at 37 °C under 5% CO_2_ in the presence or absence of 1 μg ml^−1^ bovine trypsin that was treated with l-1-tosylamido-2-phenylethyl chloromethyl ketone to inhibit contaminating chymotrypsin activity without affecting trypsin activity (Thermo Fisher Scientific). After infection, cells were washed twice with DMEM/F-12, embedded in Matrigel in a 48-well tissue culture plate and cultured in organoid growth medium at 37 °C with 5% CO_2_. Each well contained 2 × 10^4^ cells. At 24 h after plating, the samples were collected and suspended in DNA/RNA shield. The viral RNA copy number was determined as described above.

### In vitro degradation of trypsin

Overnight bacterial cultures were incubated with recombinant mouse trypsin (final concentration 1 µg ml^−1^) for 1 h or human trypsin (final concentration 20 µg ml^−1^) for 4 h. The recombinant trypsin isoforms used in this study were as follows: mouse recombinant PRSS2 (50383-M08H, Sino Biological), human recombinant PRSS1 (LS-G135640), human recombinant PRSS2 (LS-G20167) and human recombinant PRSS3 (His-tag) (NBP2-52220). In some experiments, recombinant mouse PRSS2 was first treated with one of the following trypsin inhibitors for 30 min before incubation with *P. clara*: AEBSF (Sigma-Aldrich; final concentration, 2 mM), Leupeptin (Sigma-Aldrich; final concentration, 100 µM) and TLCK (Abcam; final concentration, 100 µM). In some of the experiments *P. clara* was grown overnight in the presence of tunicamycin (Sigma-Aldrich; final concentration, 10 µg ml^−1^), 2-fluro-l-fucose (Cayman Chemical; final concentration, 250 µM) or DMSO control before incubation with recombinant mouse PRSS2. For the experiments assessing the effect of Ca^2+^, *P. clara* was grown in a low-Ca^2+^ mGAM medium with or without supplementation with 1 mM Ca^2+^ before incubation with mouse recombinant PRSS2. For experiments using *P. clara* supernatant, the *P. clara* overnight culture was filtered with a sterile filter unit with a PVDF membrane (0.22 µm pore size).

### Confocal microscopy

Recombinant mouse PRSS2 was labelled with Alexa Fluor 488 using Alexa Fluor 488 Antibody Labeling Kit (A20181, Thermo Fisher Scientific) and pretreated with AEBSF inhibitor (150 µg ml^−1^ rmPRSS2 with 20 mM AEBSF). Alexa Fluor 488-labelled mouse PRSS2 was incubated with overnight bacterial cultures at a final concentration of 5 µg ml^−1^ for 20 min in an anaerobic chamber. The mixture was centrifuged, washed with PBS once and resuspended in PBS. Leica TCS SP8 confocal microscopy was used for confocal imaging.

### DSSO cross-linking

DSSO (A33545) was purchased from Thermo Fisher Scientific. *P. clara* 1C4 was incubated with AEBSF-pretreated recombinant mouse recombinant PRSS2 (50383-M08H, Sino Biological) for 20 min, washed once with PBS and resuspended in 10 mM DSSO. The reaction was incubated at room temperature for 10 min and quenched by adding concentrated Tris-HCl buffer (final concentration, 20 mM). After washing with PBS, the pellet was lysed with 1% SDS solution (in 50 mM Tris-HCl buffer supplemented with 5 mM EDTA). *P. clara* 1C4 without incubation with PRSS2 was processed in the same manner to serve as the negative control. Lysates were stained with rabbit anti-6-His antibodies (A190-214A, Bethyl laboratories) and anti-rabbit IgG (HRP-linked antibody) (7074, Cell Signaling Technology) and analysed by western blot.

### Protein staining of whole-cell lysate, supernatant and glycan-containing proteins

*P. clara* 1C4 was cultured overnight in the presence of Tunicamycin (Sigma-Aldrich; final concentration, 10 µg ml^−1^), 2-fluro-l-fucose (Cayman Chemical; final concentration, 250 µM) or DMSO control. Cultured bacteria were then pelleted, washed once with PBS and lysed with 1% SDS solution (in 50 mM Tris-HCl buffer supplemented with 5 mM EDTA). SDS–PAGE was conducted using the Novex NuPAGE SDS–PAGE Gel system (Thermo Fisher Scientific). Glycan-containing proteins were stained with the Pro-Q Emerald 300 Glycoprotein Gel and Blot Stain Kit (Thermo Fisher Scientific) according to the manufacturer’s protocol. The protein contents of the whole-cell lysates were stained using the Colloidal Blue Staining kit (Thermo Fisher Scientific). Supernatant proteins were first condensed using Amicon Ultra Centrifugal Filters (10 kDa NMWL) and then stained using the Colloidal Blue Staining kit (Thermo Fisher Scientific).

### Mutant generation

The deletion mutants (*Δ03049-03053, Δ00502 and Δ00509*) of *P. clara* JCM14859 were generated as previously described^30^ with minor modifications. In brief, approximately 1 kb sequences flanking the coding region were amplified by PCR and assembled into the suicide vector pLGB30 using HiFi DNA Assembly (NEB) according to the manufacturer’s protocol. Aliquots of each reaction (1 μl) were transformed into electrocompetent *Escherichia coli* S17-1 λpir. Transformants were conjugated with *P. clara* JCM14859 as follows. The donor and recipient strains were cultured in LB and EGEF media, respectively, to an OD_600_ of 0.5 and mixed at a ratio of 1:1. The mixture was dropped onto an EGEF agar plate and incubated aerobically at 37 °C for 16 h. Transconjugants were selected on EGEF agar plates containing tetracycline (10 μg ml^−1^). Transconjugants were partially sensitive to rhamnose-induced ss-bfe1 toxin expression and, in the presence of 10 mM rhamnose, their growth was inhibited (with an overnight OD_600_ of ~0.3). Subsequently, to select for loss of the plasmid from the genome by a second crossover, transconjugants were cultured in EGEF broth supplemented with 10 mM rhamnose for at least three generations until the transconjugants were outcompeted by the revertants (overnight OD_600_ reached ~1.0). The bacterial culture was then plated, single colonies were picked and successful deletions were confirmed by PCR. For generation of insertional mutants, a similar protocol was used: approximately 0.5–1 kb homologous sequences of the coding regions were assembled into the suicide vector pLGB30 and transformed into electrocompetent *E. coli* S17-1 λpir. Transformants were conjugated with *P. clara* JCM14859 using the same protocol and transconjugants were selected on EGEF agar plates containing tetracycline (10 μg ml^−1^), confirmed by PCR and maintained in EGEF broth supplemented with tetracycline (10 μg ml^−1^). A list of all of the primers used for mutagenesis is provided in Supplementary Table 5.

### Transmission electron microscopy

WT or *Δ00502 P. clara* JCM14589 strains were incubated with mouse recombinant PRSS2 (50383-M08H, Sino Biological; final concentration, 5 µg ml^−1^) for 20 min, washed with PBS and fixed with 4% paraformaldehyde-1% glutaraldehyde solution at room temperature for 2 h. After washing with 0.05 M PBS, the pellets were dehydrated in a graded series of ethanol (50%, 70%, 80%, 90%, 95% and 100%). The dehydrated pellets were infiltrated with LRW resin (1:1 of 100% ethanol and LRW for 1 h, then 1:2 of 100% ethanol and LRW overnight, and then 100% LRW for 5 h). After infiltration, the samples were cured in gelatin capsules (53 °C for 24 h). Polymerized LRW blocks were sectioned using the Leica Ultracut UCT and 80 nm sections were obtained. For immunogold staining, sections were first blocked with 0.05 M PBS supplemented with 1% BSA, followed by staining with rabbit anti-6-His antibodies (A190-214A, Bethyl laboratories) for 60 min. After washing with 0.05 M PBS, the sections were stained with 12 nm Colloidal Gold goat anti-rabbit IgG for 60 min. After washing again with 0.05 M PBS, the sections were fixed with 1% glutaraldehyde in 0.05 M PBS, washed with H_2_O and stained with uranyl acetate for 5 min. All of the images were taken using the JEOL JEM-1400 transmission electron microscope.

### Recombinant protein expression, coupling to magnet microbeads and blue native gel electrophoresis

For generation of recombinant 00502 and 00509, the coding regions of both genes (excluding the N-terminal sequences encoding the signal peptides) were cloned into the expression vector pET-28b (+) (Novagen, 69865) to introduce a C-terminal His-tag according to the supplier’s protocol. Expression vectors were transformed into Rosetta-gami B(DE3) competent cells (Novagen, 71136). Transformants were grown to the exponential phase and protein expression was induced by supplementation with 0.4 mM IPTG (Sigma-Aldrich, I6758). After overnight culture at 25 °C, cells were lysed with the B-PER Bacterial Protein Extraction Reagent (Thermo Fisher Scientific, 78243), and recombinant 00502 and 00509 were purified with the Pierce Ni-NTA Magnetic Agarose Beads (Thermo Fisher Scientific, 78605) and Pierce Polyacrylamide Spin Desalting Columns (Thermo Fisher Scientific, 89849). Purified recombinant 00502 and 00509 or bovine serum albumin (Thermo Fisher Scientific, 23209) were coupled to the micromagnetic beads (Dynabeads) with the Dynabeads Antibody Coupling kit (Thermo Fisher Scientific, 14311D) according to the manufacturer’s protocol, with 15 μg protein input per mg of beads. For downstream analyses, 1 mg protein-coupled Dynabeads was resuspended in 200 μl EGEF medium and mixed with recombinant mouse PRSS2 (final concentration 3 µg ml^−1^), AEBSF-pretreated Alexa Fluor 488-labelled recombinant mouse PRSS2 (final concentration 5 µg ml^−1^) or 50 μl GF caecal contents (50-fold dilution in PBS). For blue native gel electrophoresis, recombinant 00502 and 00509 were purified with anion-exchange and nickel-affinity chromatography from r00502- or r00509-expressing Rosetta-gami B(DE3) *E. coli*. The Native PAGE Bis-Tris Gel System (Thermo Fisher Scientific, BN1002BOX and BN2007) was used according to the manufacturer’s protocol. To detect the r00502–trypsin complex, 100 µg ml^−1^ or 400 µg ml^−1^ recombinant human PRSS2 was pretreated with 20 mM AEBSF trypsin inhibitor for 30 min, incubated with r00502 (100 µg ml^−1^) and then loaded to native PAGE gels. SERVANativ Marker Liquid Mix (SERVA, 39219) was used as the protein standard. For western blot analysis of blue native gels, proteins were blotted using the iBlot 2 Dry Blotting System with PVDF membranes (Thermo Fisher Scientific). A list of the primers used for the generation of the recombinants is provided in Supplementary Table 5.

### Protease activity assay

The Pierce Fluorescent Protease Assay Kit (Thermo Fisher Scientific, 23266) was used to determine the protease activity of the *P. clara* culture, the *P. clara* culture supernatant, and recombinant 00502 and 00509 according to the manufacturer’s protocol. The PerkinElmer 2030 Multilabel Reader with fluorescein excitation and emission filters (485/538 nm) was used to detect increased total fluorescence as the fluorescein isothiocyanate (FITC)–casein substrate was digested by proteases into smaller fluorescein-labelled fragments. Protease activity was expressed as change in relative fluorescence units (RFU).

### Ex vivo degradation of IgA by faecal and recombinant trypsin

Faeces from the 2-mix+WT *P. clara*-colonized mice and GF mice was filtered to remove the bacteria, diluted 50-fold in PBS, mixed at a ratio of 1:1 (in the presence or absence of 100 µM trypsin inhibitor TLCK) or mixed with an equal volume of PBS (final dilution 100-fold), followed by incubation at 37 °C for 24 h. Alternatively, filtered and diluted (100-fold in PBS) faeces from the 2-mix+WT *P. clara*-colonized mice was incubated at 37 °C for 24 h with different concentrations of recombinant mouse PRSS2 (0–16 µg ml^−1^). After incubation, the trypsin activity and the protein contents of the samples were analysed using a trypsin-activity assay and western blotting as described above.

### Metagenomic analysis of the human gut microbiome

Metagenomes from human faecal samples from PRISM^52^, HMP2^53^, FHS^36^, 500FG^54^, CVON^55^ and Jie^56^ were de novo assembled into a non-redundant gene catalogue, compiled into metagenomic species using MSPminer^57^ and quantified in terms of relative abundance in a previous study^36^. To search in the gene catalogue for the homologues of *P. clara* and *P. xylanphila* genes from the trypsin-associated locus containing the genes *00502* and *00509*, as well six other neighbouring genes, we used USEARCH^58^ UBLAST (at protein level) retaining hits with a minimum *e* value of 0.1. We confirmed the presence of all 8 genes in both species in the gene catalogue. To identify additional plausible homologues and species encoding this locus, we first evaluated the similarity between the corresponding homologues in *P. clara* and *P. xylanphila*, and set the following thresholds of minimal identity (Id) and coverage (Cov) for UBLAST hits to each gene in the locus: *00502*, Id = 25%, Cov = 90%; *00503*, Id = 70%, Cov = 90%; *00504*, Id = 60%, Cov = 90%; *00505*, Id = 60%, Cov = 90%; *00506*, Id = 50%, Cov = 90%; *00507*, Id = 25%, Cov = 90%; *00508*, Id = 45%, Cov = 80%; *00509*, Id = 20%, Cov = 30%. We then evaluated which other metagenomic species encoded homologues of *P. clara* and *P. xylanphila 00502*-*00509*, identifying MSP 0355 and MSP 0303. Although MSP 0355 and MSP 0303 were previously annotated to only the phylum Bacteroidetes^36^, we used UBLAST to compare their proteomes to the unified human gastrointestinal genome (UHGG) collection^59^. In both cases, most of the genes (>90%) mapped with high confidence (median amino acid identity >99% and *e* < 1 × 10^−184^) to a single species representative in UHGG, annotating MSP 0355 and MSP 0303 as GUT_GENOME140082 and GUT_GENOME016875, respectively; in UHGG^59^, both were phylogenetically classified as *Paraprevotella* spp. Moreover, we identified five MSPs that encoded homologues of only *00502* and *00509*: MSP 0081, MSP 0224, MSP 0288, MSP 0410 and MSP 0435. To evaluate which individuals in the COVID-19 cohort (described below) carried *P. clara*’s gene *00502* or its homologues, we quality controlled faecal metagenomic data using Trim_Galore! to detect and remove sequencing adapters (minimum overlap of 5 bp) and KneadData v.0.7.2 to remove human DNA contamination and trim low-quality sequences (HEADCROP:15, SLIDINGWINDOW:1:20), and retained reads that were at least 50 bp long. Paired-end quality-filtered reads were mapped to the same gene catalogue from a previous study^36^ with BWA^60^, filtered to include strong mappings with at least 95% sequence identity over the length of the read, counted and normalized to transcripts per million (TPM matrix). Detection (TMP > 0) of any of the *00502* homologues classified the sample as containing a *00502* gene in their gut microbiome. All of the metagenomic samples in the COVID-19 cohort had at least 8 million reads after quality filtering.

### AlphaFold modelling

The amino acids sequences of 00502 from *P. clara*, *P. xylaniohila*, *P. rara*, *P. rodentium* and *P. muris* were retrieved from GenBank (NZ_JH376591, EGG54658, LFQU01000025, NZ_JABKKH010000006 and NZ_JABKKF010000005, respectively). 00502 models were predicted using AlphaFold2 (ref. ^61^) through ColabFold^62^—an online platform for protein folding. Model confidence was evaluated through pLDDT scores, with a pLDDT > 90 considered to be very high model confidence. The resulting AlphaFold models were then aligned in PyMOL (Schrödinger) and visualized in ChimeraX^63^.

### COVID-19 cohort

The COVID-19 cohort was recruited as a part of the Japan COVID-19 Task Force (JCTF) study^64^. According to the study protocol approved by the institutional review board at Keio University (code 20190337), we recruited 146 patients who were diagnosed as having COVID-19 by physicians using the clinical manifestation and PCR test results and were hospitalized at Keio University Hospital from March 2020 to September 2021. Informed consent was obtained from each participant. Approximately 2 months after discharge from the hospital, faecal samples were collected and sent to the laboratory in DNA/RNA Shield (Zymo Research). Among the 146 participants, information of oxygen inhalation was available for all participants, whereas that of diarrhoea incidence was available for 141 cases from the medical records during hospital care. Microbial DNA was extracted from 100 μl of faecal suspension as described above. Extracted DNA was sheared using M220 Focused-ultrasonicater (Covaris) to obtain fragmented DNA of around 500 bp. Metagenomic sequencing libraries were prepared from 200 ng of fragmented DNA using the TruSeq DNA Nano Library Preparation kit with IDT for Illumina-TruSeq DNA UD Indexes (Illumina) according to the manufacturer’s recommended protocol. Libraries were pooled by equal DNA amount, and library size and concentration were evaluated using the 4200 TapeStation (Agilent Technologies) and Qubit 3 Fluorometer (Invitrogen), respectively. Sequencing was performed on the Illumina NovaSeq 6000 system with 151 bp paired-end reads. The quality control for the metagenomic data was conducted using ParDRe v.2.1.5 (ref. ^65^) to remove duplicated reads, and fastp v.0.20.0 (ref. ^66^) to remove low-quality sequences (<Q20, 50% of bases), adapter sequences and polyG tails. Minimap2 v.2.17 (ref. ^67^) was used to remove PhiX and human DNA contamination.

### Statistics

All statistical analyses were performed using GraphPad Prism software (GraphPad Software) and Excel. One-way ANOVA with Tukey’s test was used for multiple comparisons. Mann–Whitney *U*-tests with Welch’s correction (nonparametric) or unpaired *t*-tests (parametric) were used for comparisons between two groups. Spearman rank correlation was used to investigate the correlation between two variables. log-rank (Mantel-Cox) tests were used for survival analysis. One-sided Fisher’s tests were used to determine whether two groups differ in the proportion with which they fall into the two classifications.

### Reporting summary

Further information on research design is available in the Nature Research Reporting Summary linked to this article.

## Online content

Any methods, additional references, Nature Research reporting summaries, source data, extended data, supplementary information, acknowledgements, peer review information; details of author contributions and competing interests; and statements of data and code availability are available at 10.1038/s41586-022-05181-3.

## Supplementary information

Supplementary Figure 1 Uncropped images used to prepare the main and extended data figures.

Reporting Summary

Supplementary Table 1 Proteomic analysis of the caecal contents from SPF and GF mice.

Supplementary Table 2 Peptidomic analysis of GF mouse caecal contents incubated with *P. clara* 1C4 or medium control.

Supplementary Table 3 Proteomic analysis of the selected native PAGE gel bands.

Supplementary Table 4 Characteristics of the COVID-19 cohorts.

Supplementary Table 5 Primer information.

Peer Review File

## Data Availability

The sequenced *Paraprevotella* genome (accession code: DRA014249) and the 16S rRNA sequence data (accession code: DRA013874) are deposited in the DNA Data Bank of Japan. Metagenomic data of the COVID-19 cohort are deposited in NCBI under BioProject PRJNA821237. Proteomics and peptidomics data are deposited in the ProteomeXchange Consortium via the jPOST partner repository (IDs: PXD027678 and PXD032242). Publicly available datasets of the mouse proteome database (https://www.uniprot.org/proteomes/UP000000589) and human PRSS2 protein sequence (https://www.uniprot.org/uniprotkb/P07478/entry) were used in this study. Source data are provided with this paper.

## Source data

Source Data Fig. 1
Source Data Fig. 2
Source Data Fig. 3
Source Data Fig. 4
Source Data Extended Data Fig. 1
Source Data Extended Data Fig. 2
Source Data Extended Data Fig. 3
Source Data Extended Data Fig. 6
Source Data Extended Data Fig. 7
Source Data Extended Data Fig. 9
Source Data Extended Data Fig. 10
Source Data Extended Data Fig. 11
Source Data Extended Data Fig. 12

## Code availability

No code was developed for this analysis.
