# Supplementary material for: Identification of trypsin-degrading commensals in the large intestine
Source: Nature. 2022 Sep 7;609(7927):582–9. doi: 10.1038/s41586-022-05181-3 (PMC9477747; doi:10.1038/s41586-022-05181-3)

nature portfolio

https://doi.org/10.1038/s41586-022-05181-3

Supplementary information

Identification of trypsin-degrading commensals in the large intestine

In the format provided by the authors and unedited

Supplementary Figure 1

Original source images for data obtained by electrophoretic separation

Fig. 1c PRSS2

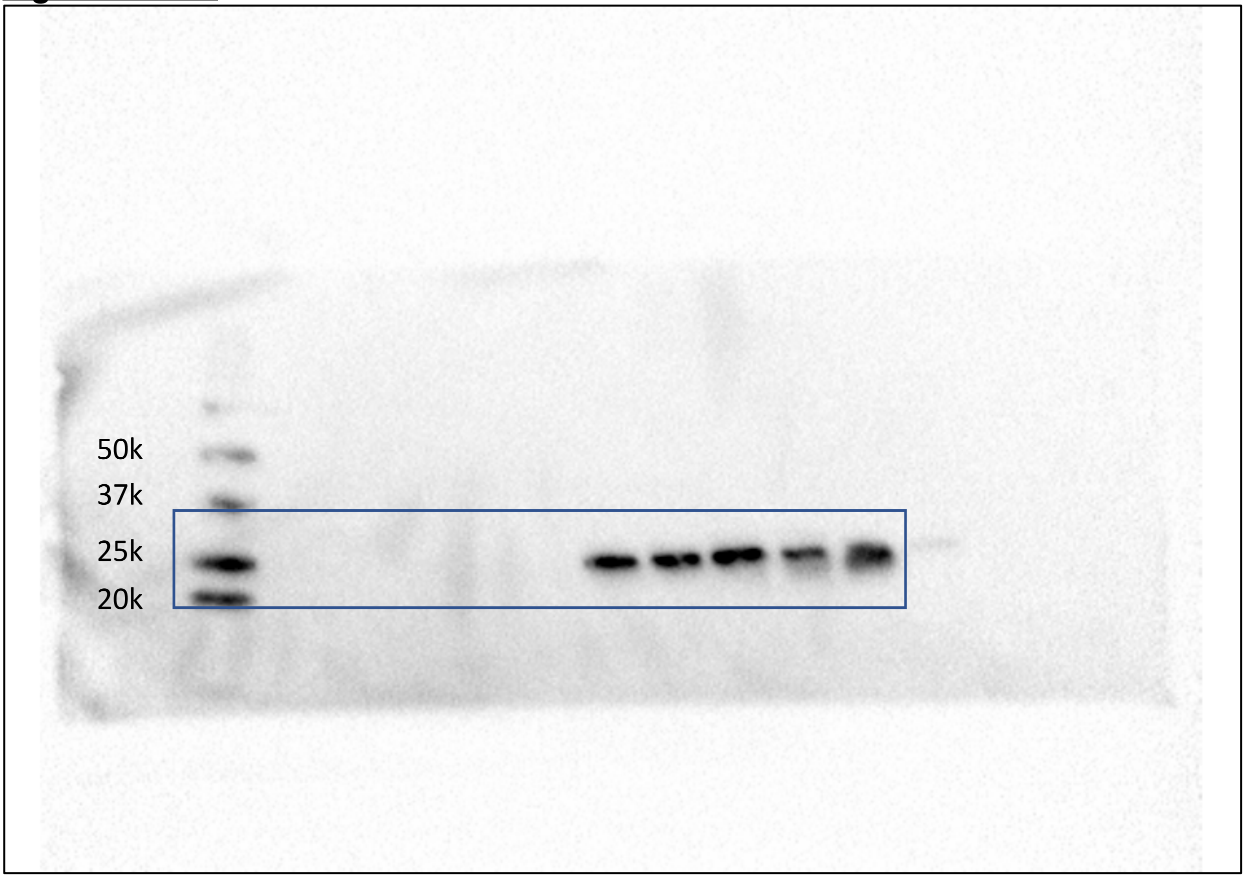

Fig. 1f top panel PRSS2

Note: positions of markers were based on the positions of the markers on the blotting membrane. The markers used in this experiment did not have an HRP conjugate hence hardly visible when scanned.

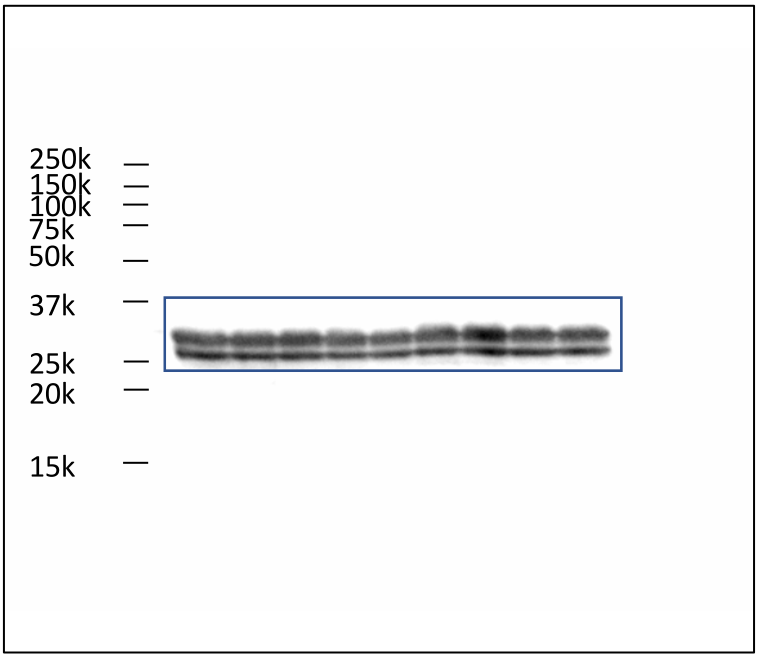

Long exposure time (from the notebook, for reference)

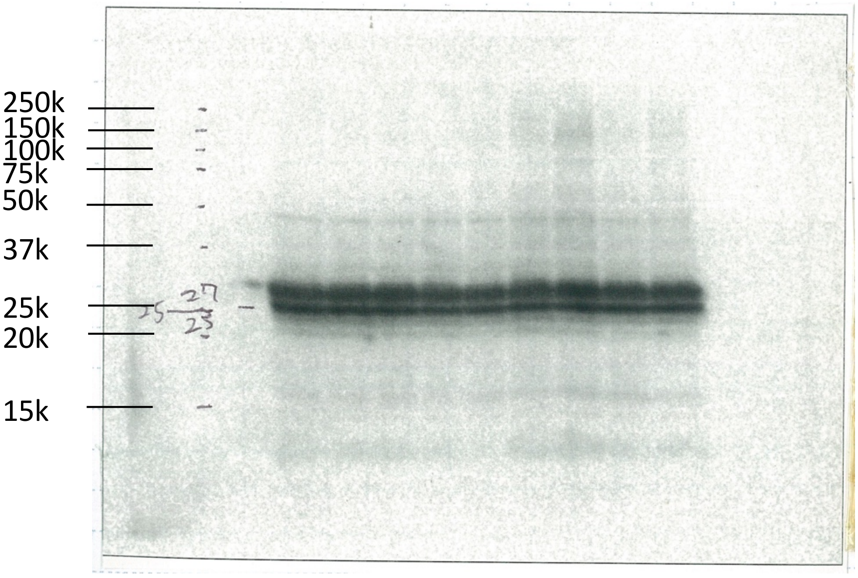

Fig 1f Bottom panel Hsp90 (separate membrane)

Note: positions of markers were based on the positions of the markers on the blotting membrane. The markers used in this experiment did not have an HRP conjugate hence hardly visible when scanned.

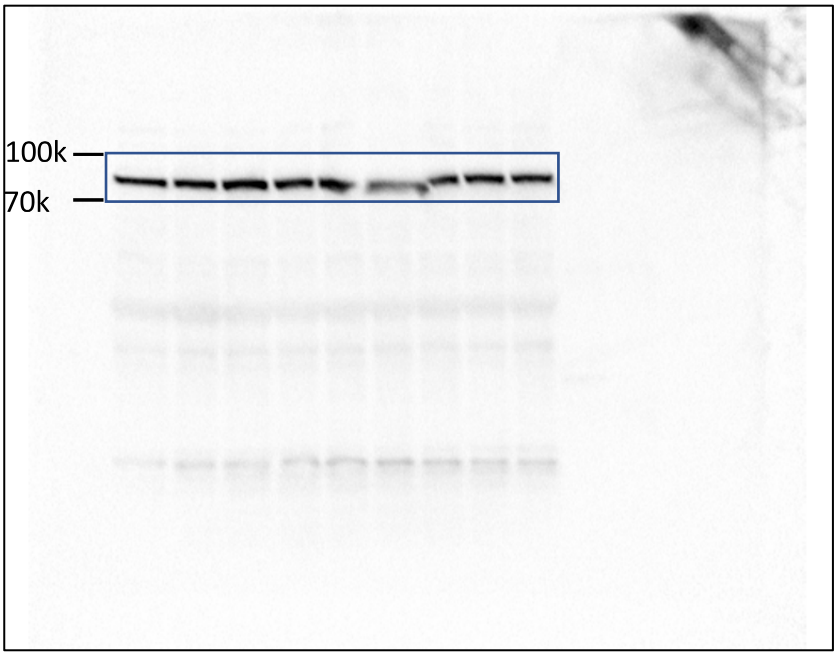

Original data from the notebook for reference:

short exposure time (from the notebook)

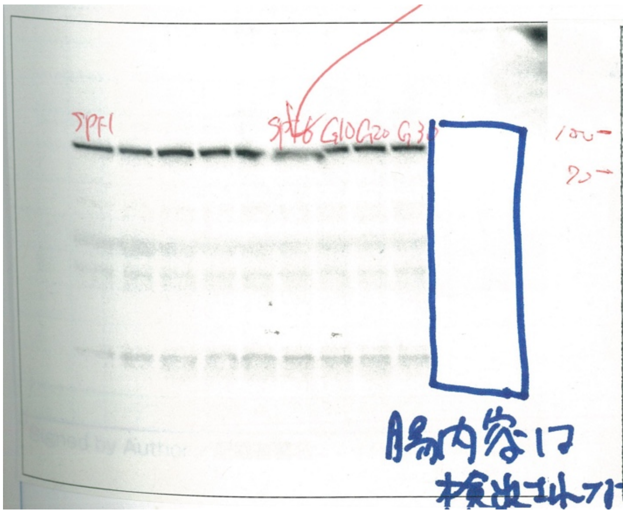

Long exposure time (from the notebook)

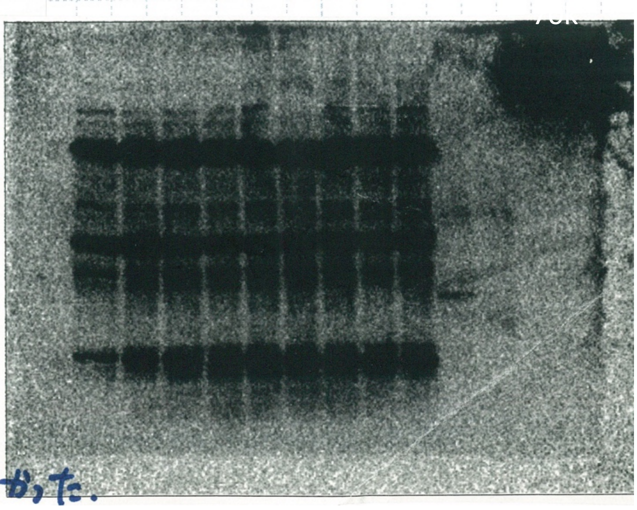

Fig 2f (rmPRSS2)

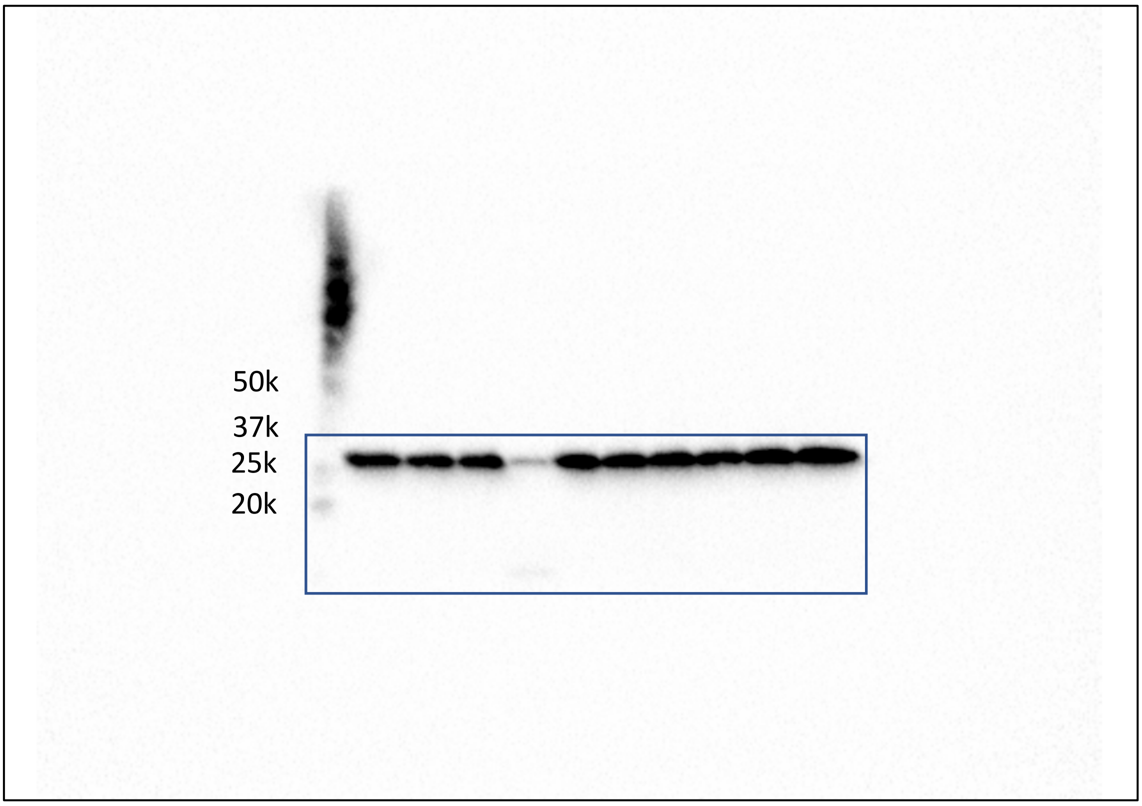

Fig 2i, left panel, hPRSS1

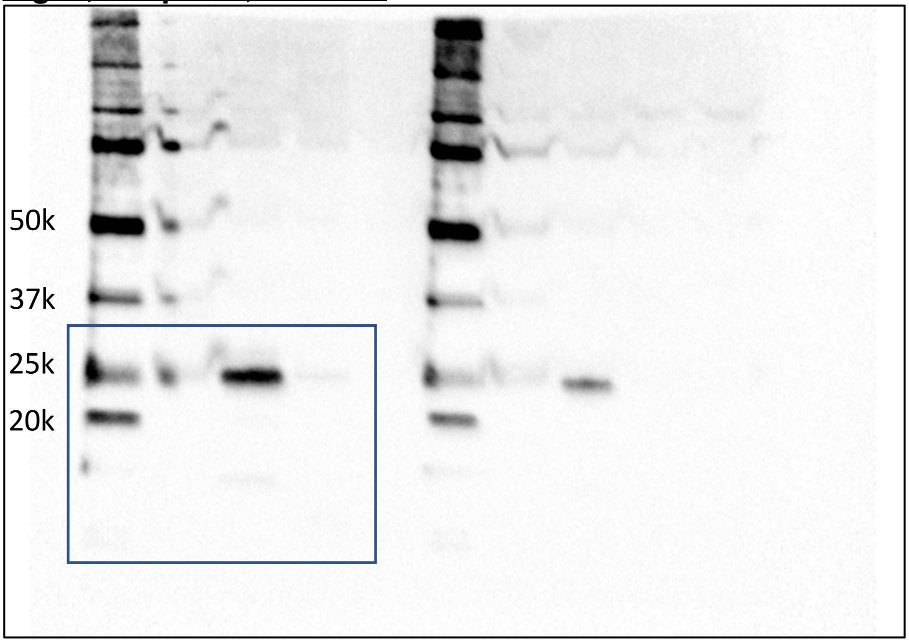

Fig 2i, middle panel, hPRSS2

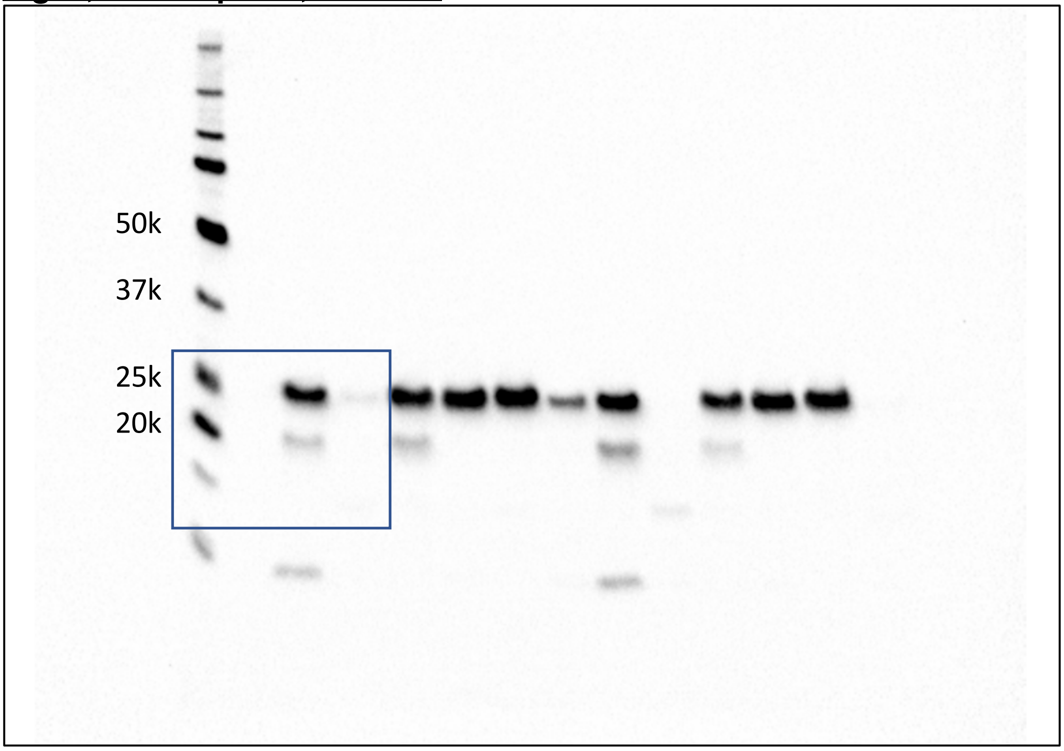

Fig 2i, right panel, hPRSS3

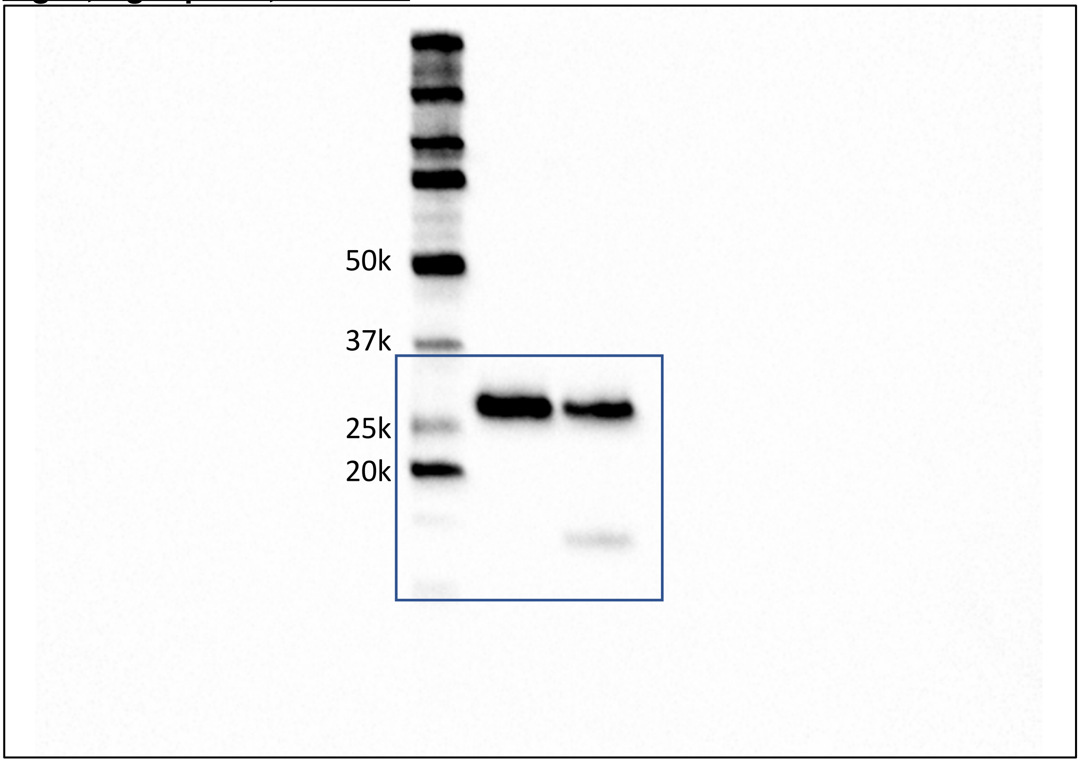

Fig 2j (rmPRSS2)

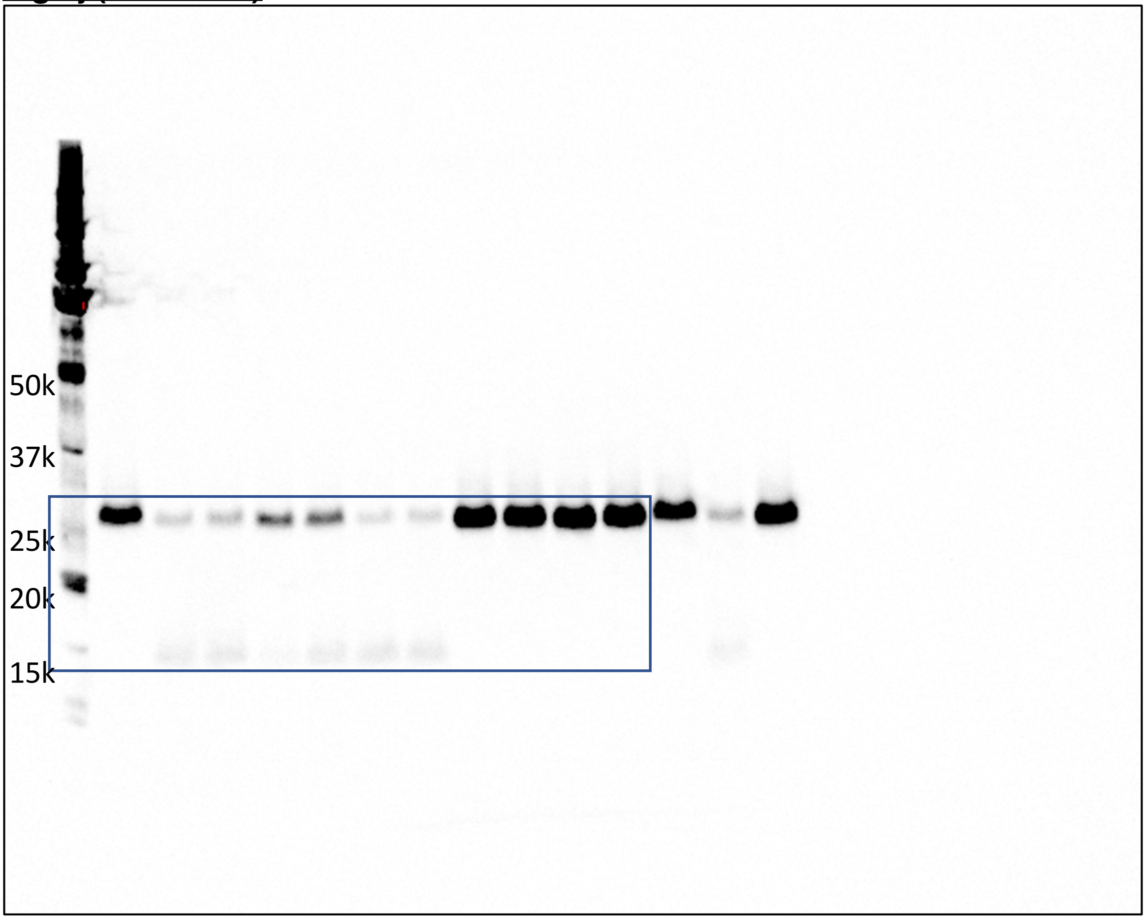

Fig 3a, rmPRSS2

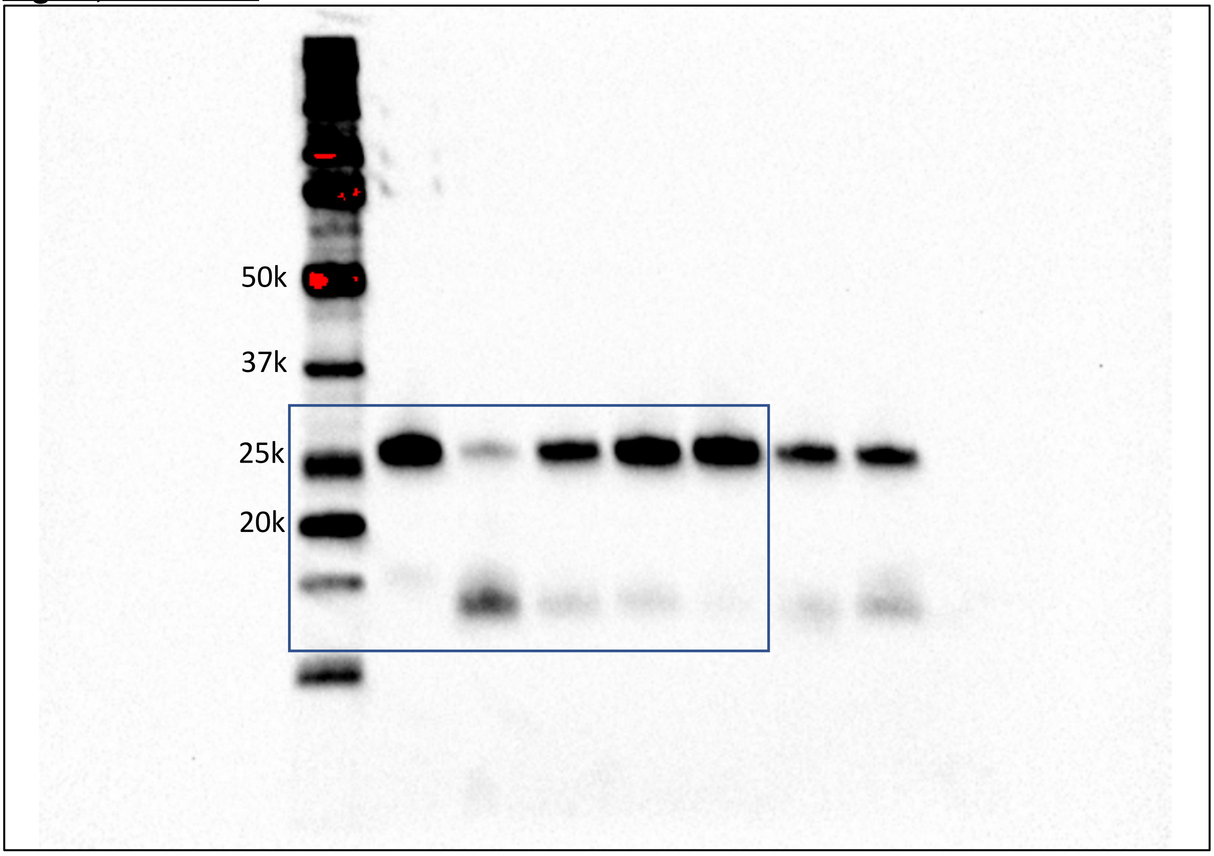

Fig 3c, rmPRSS2

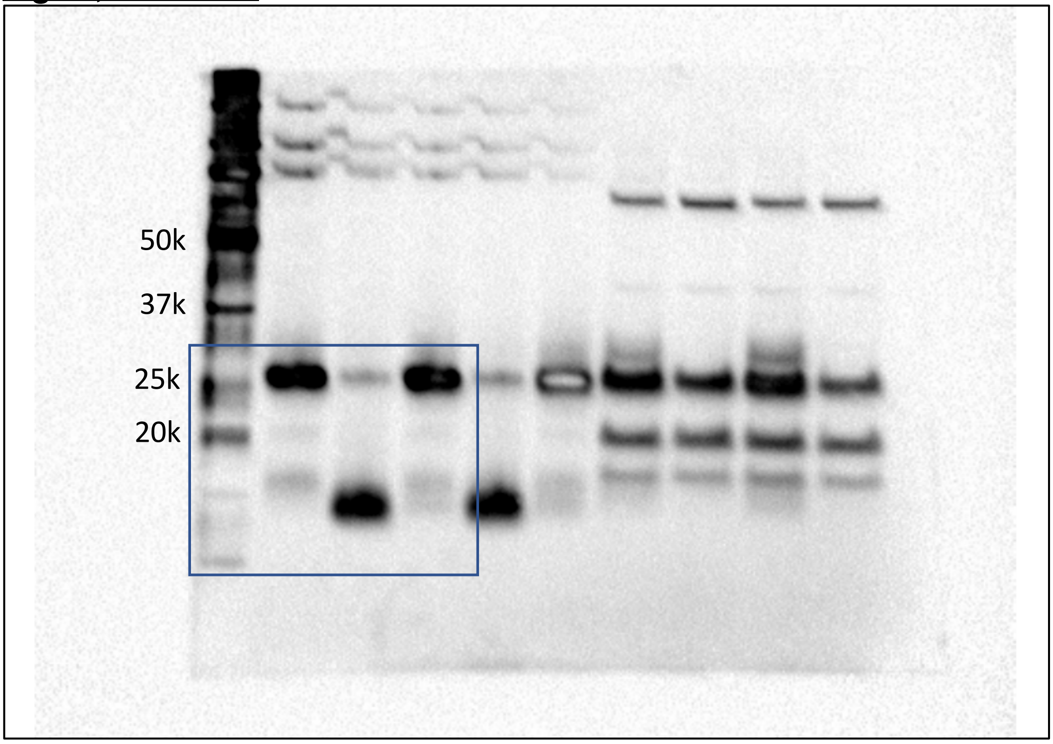

Fig 3e, rmPRSS2

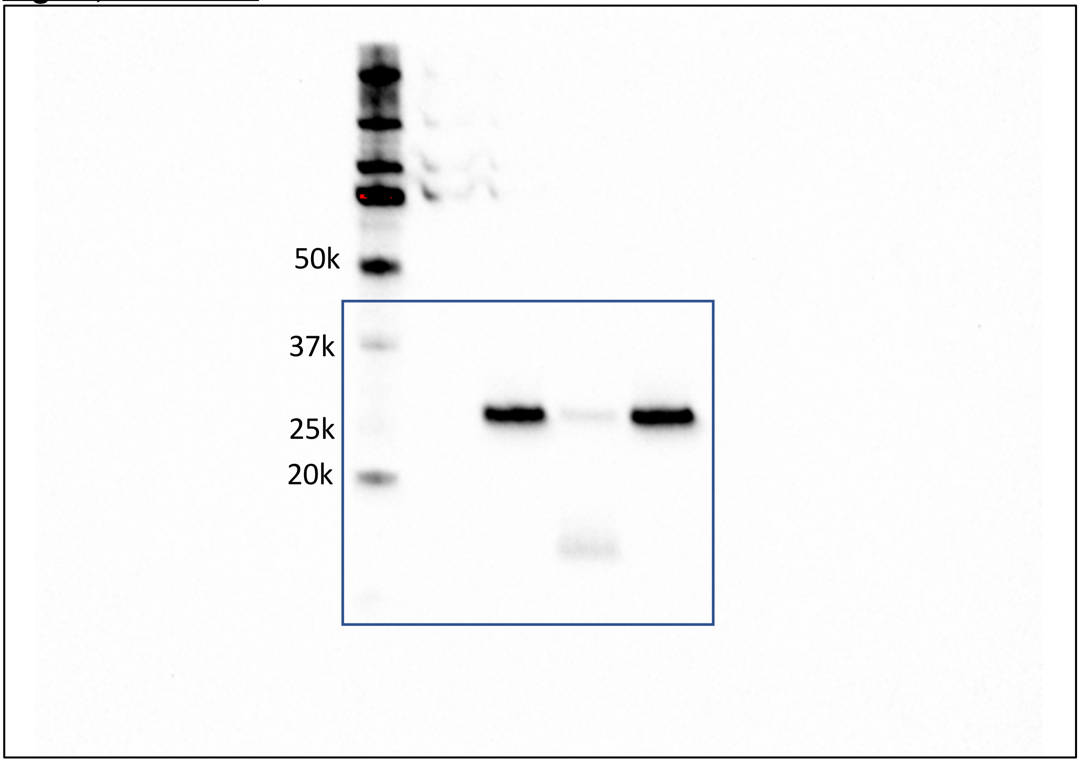

Fig 3g, left panel, rmPRSS2

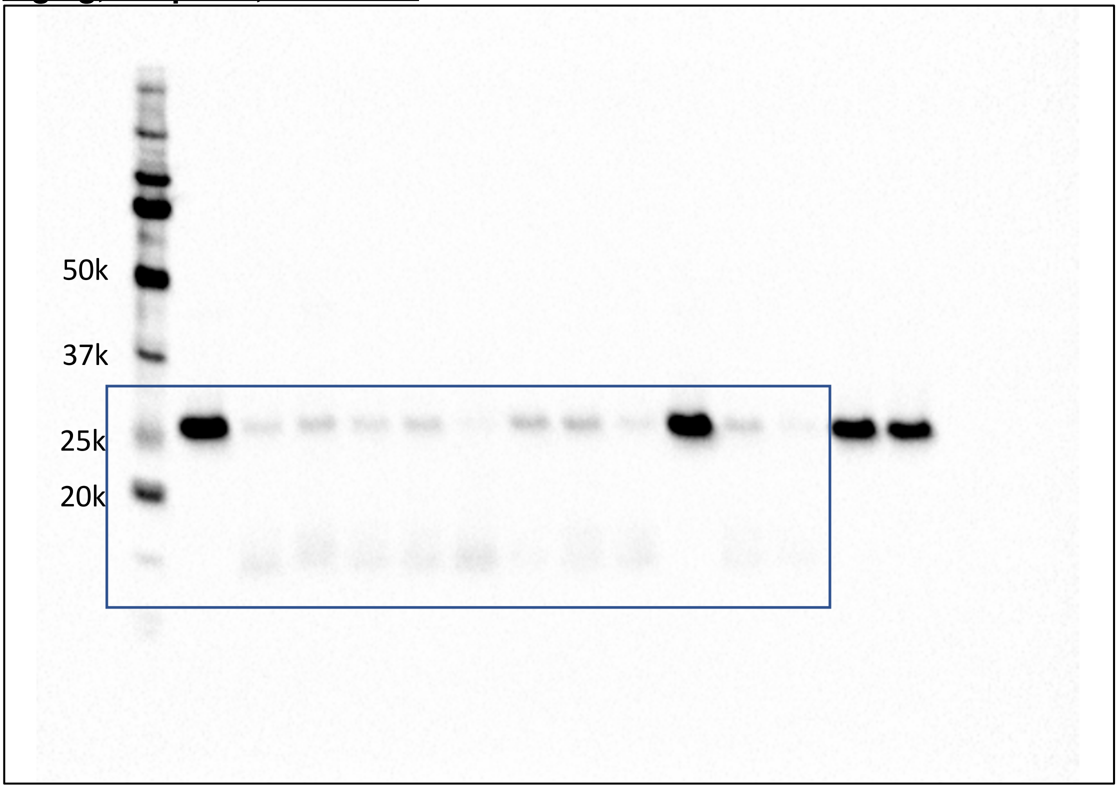

Fig 3g, right panel, rmPRSS2

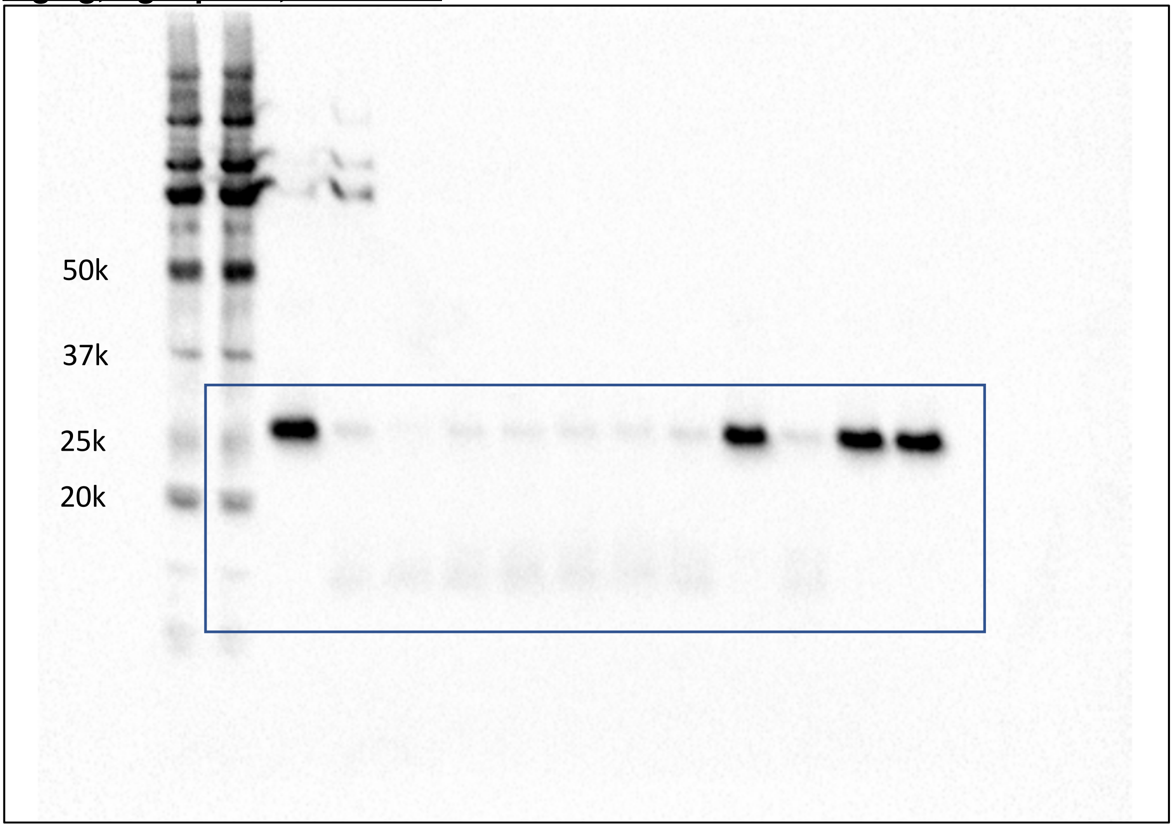

Fig 3j, rmPRSS2

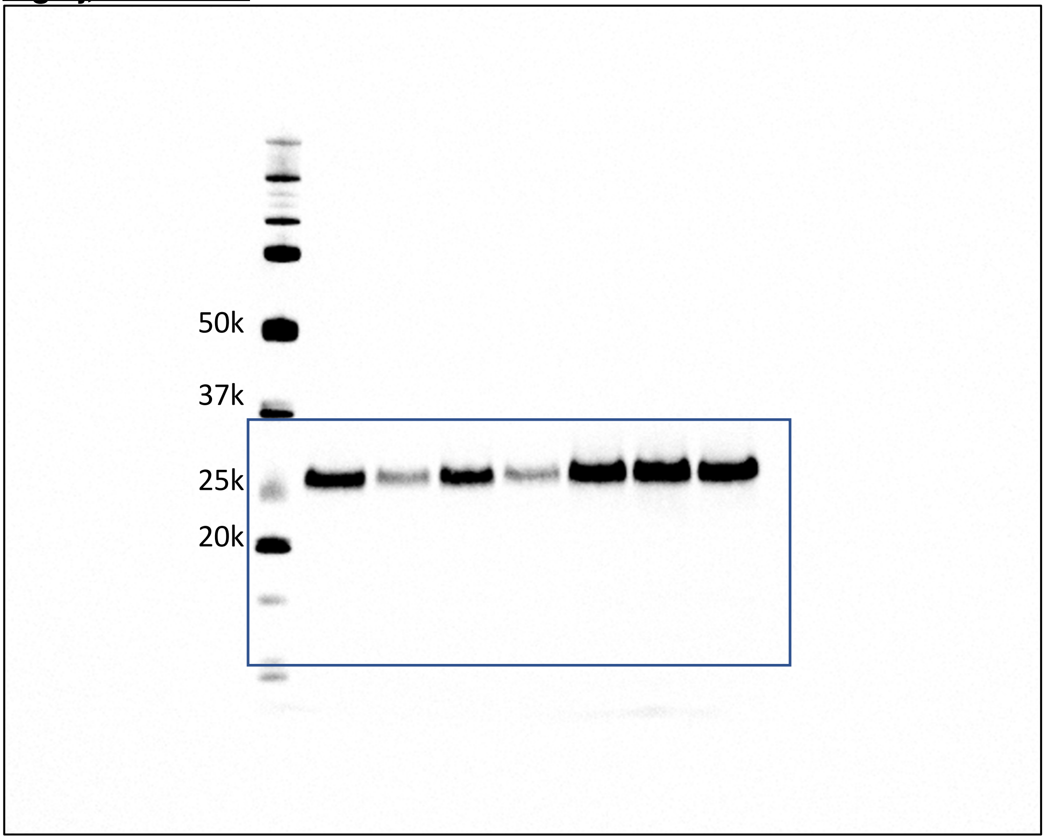

Fig 4b, panel 1, PRSS2

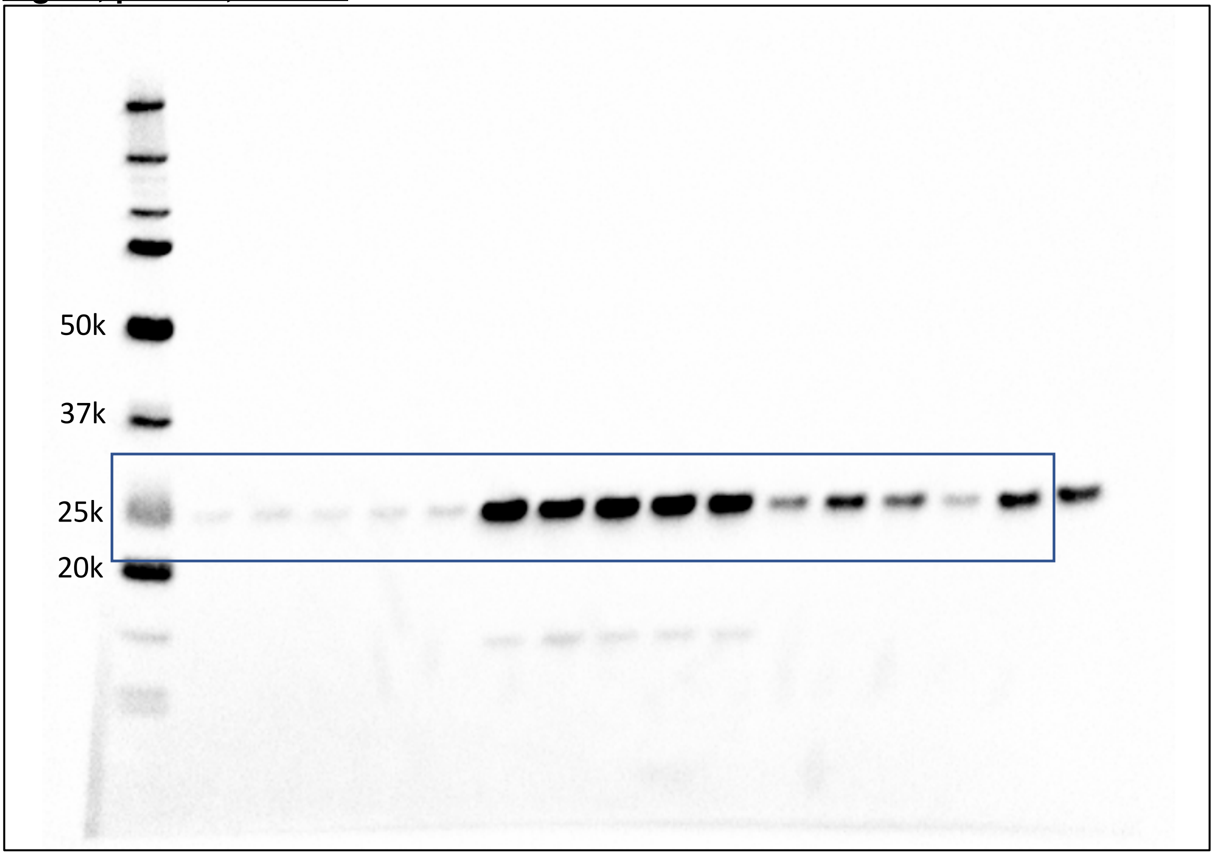

Fig 4b, panel 2, TMPRSS2

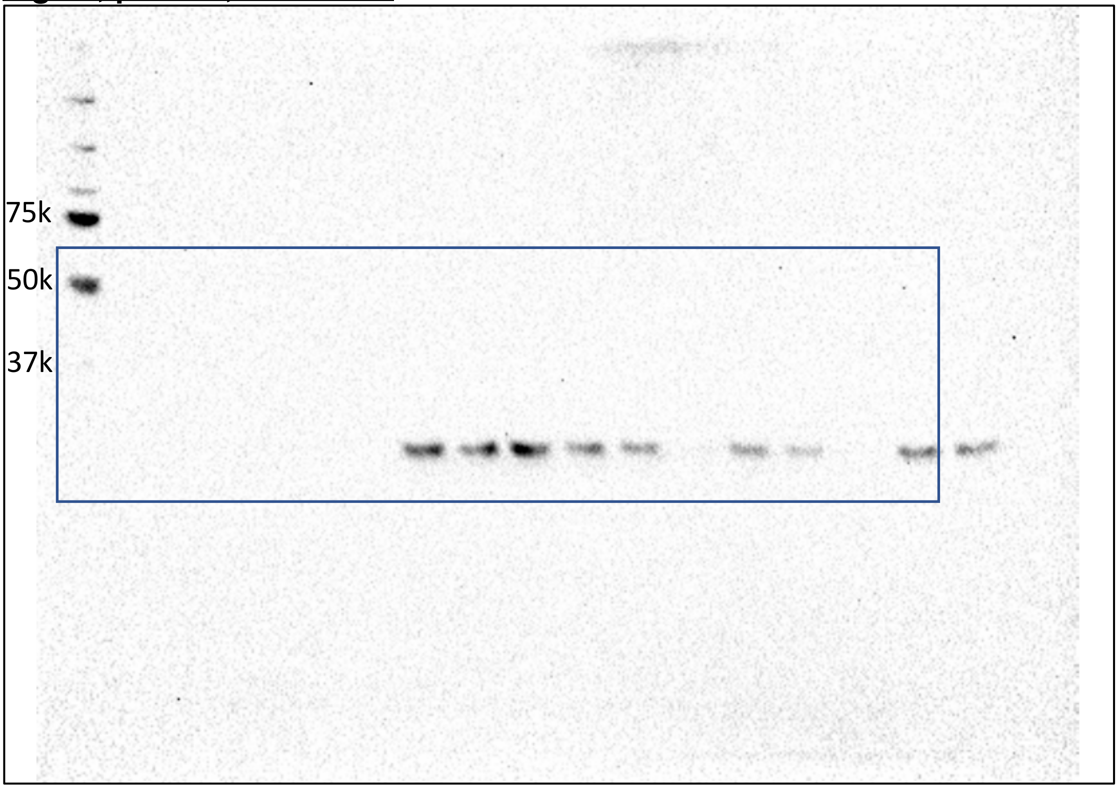

Fig 4b, panel 3, IgA (α chain)

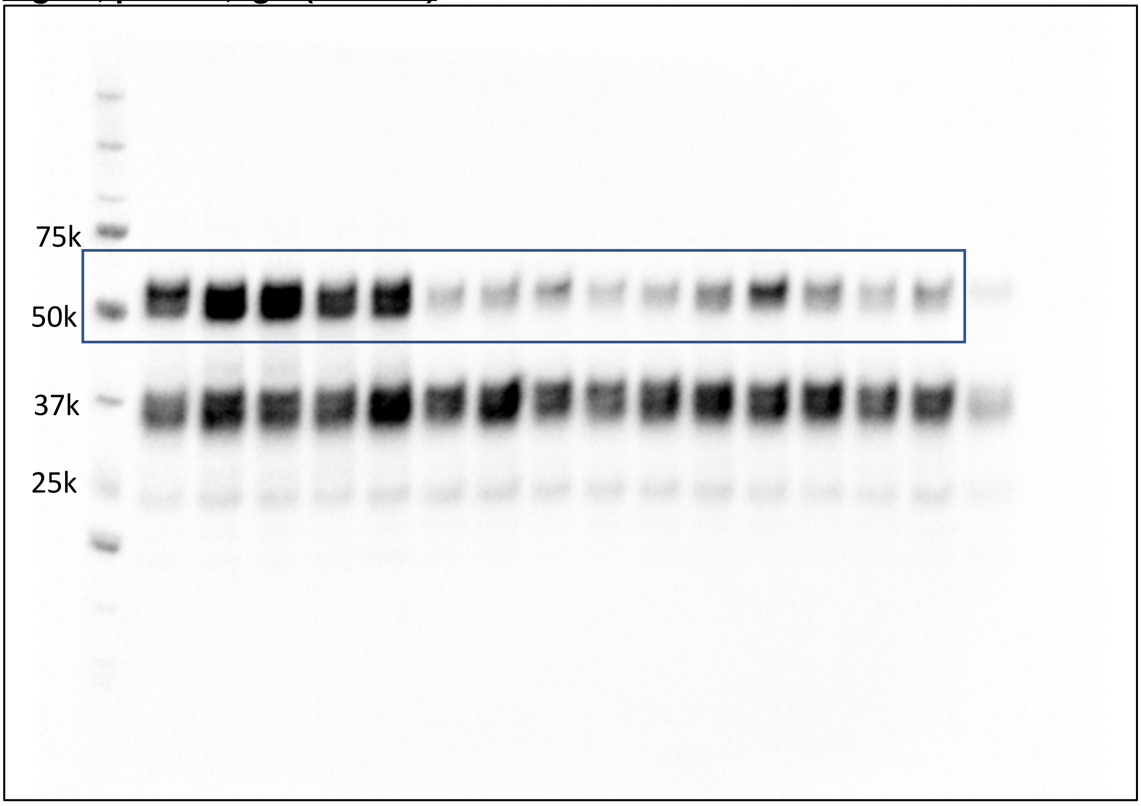

Fig 4b, panel 4, κ chain

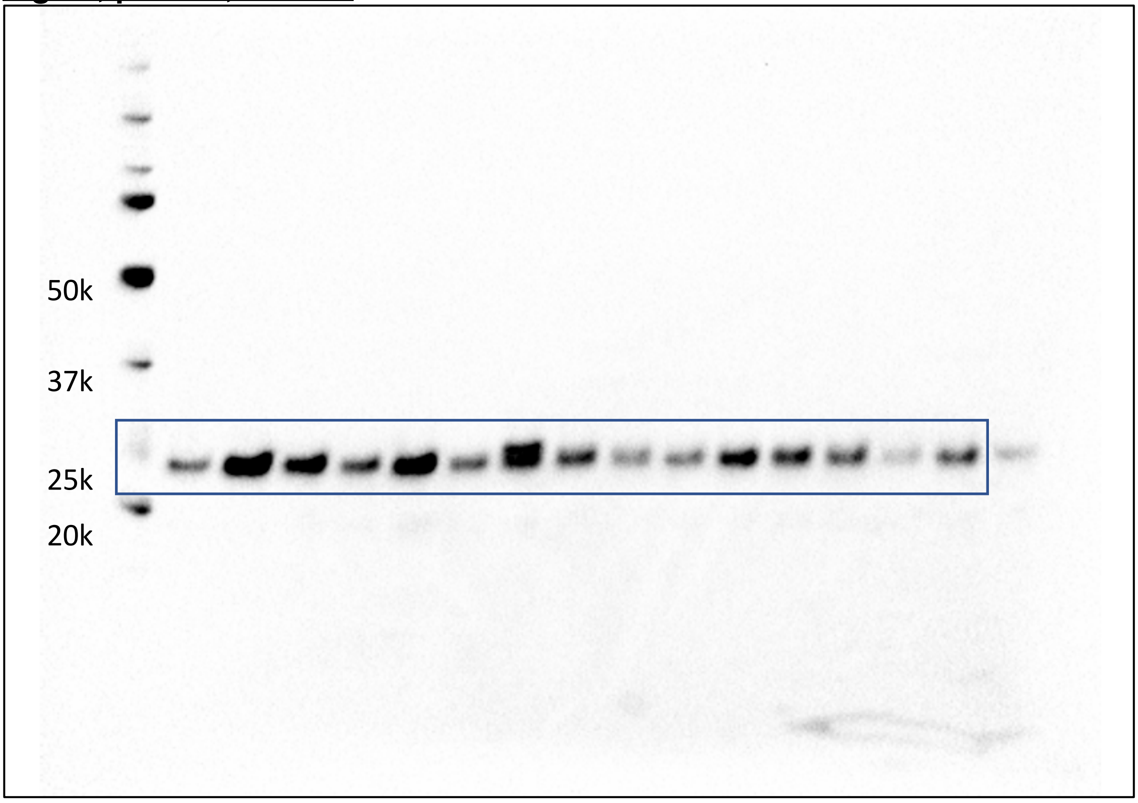

Fig 4b, panel 5, Reg3β

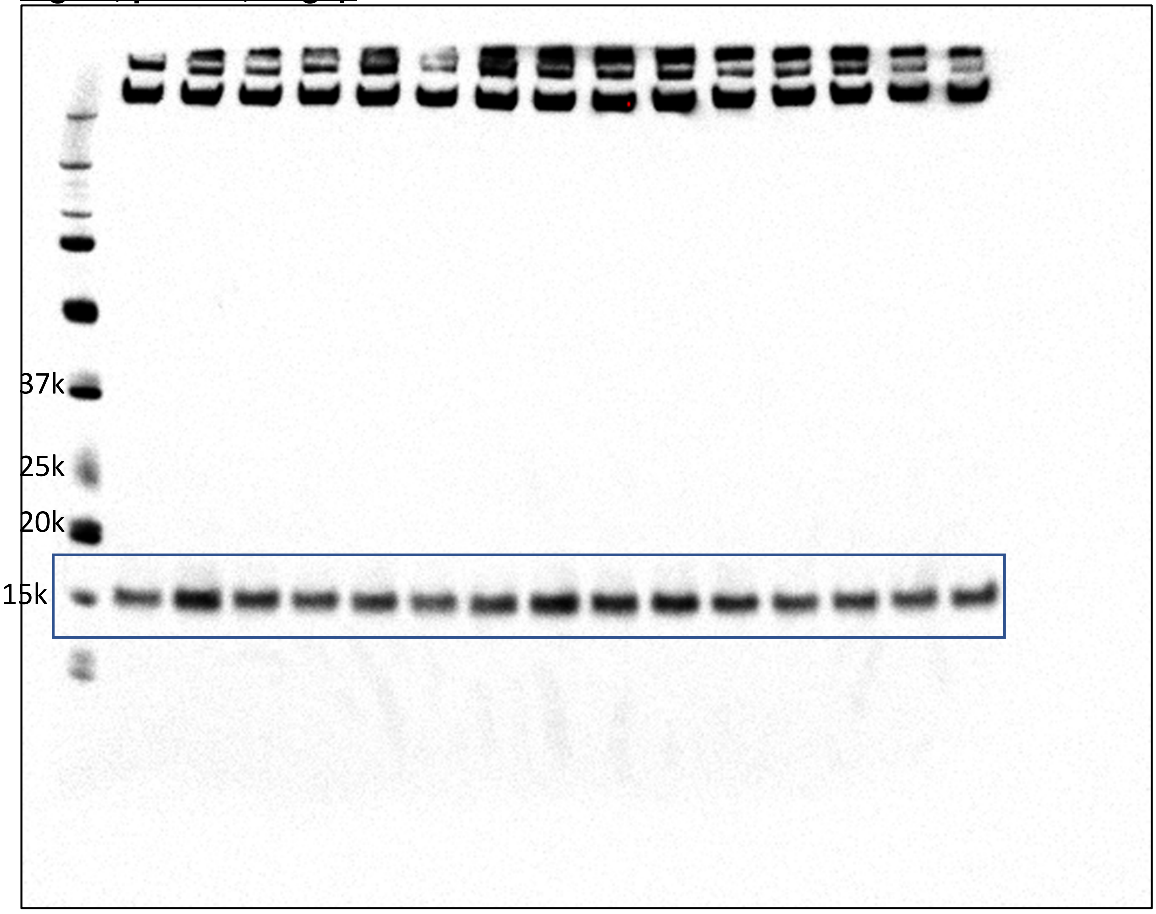

Fig 4b, panel 6, CELA3B, same membrane with Reg3β

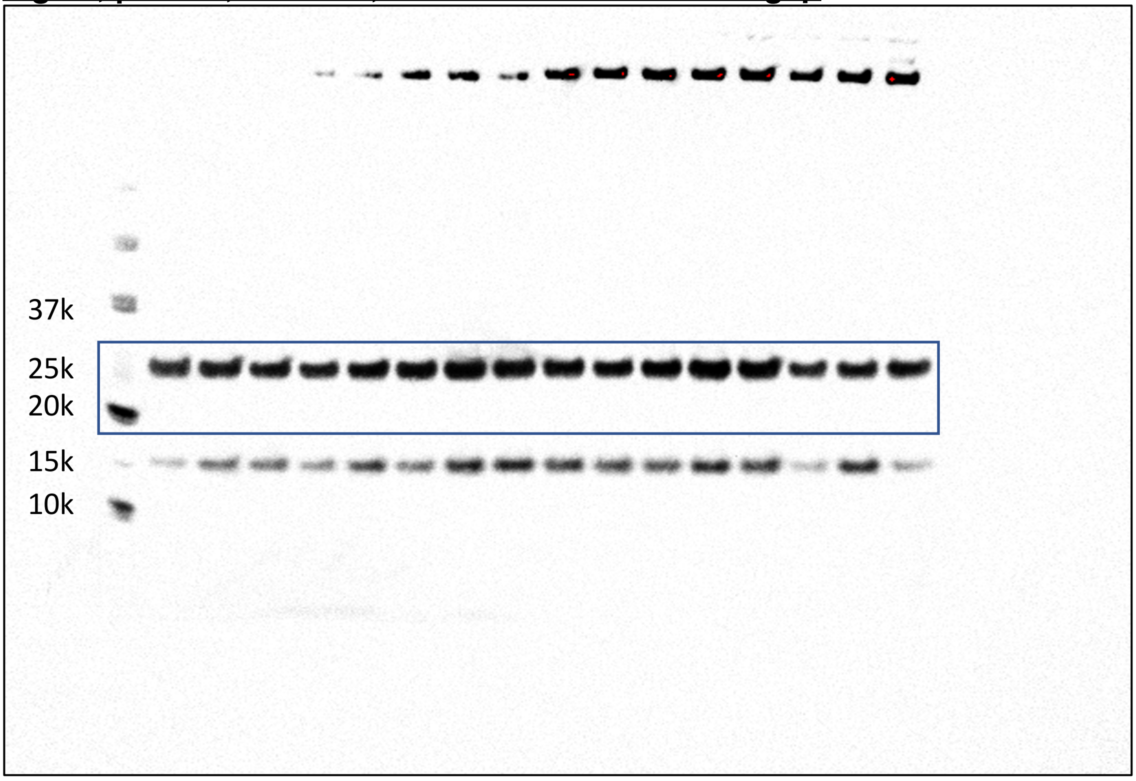

Extended Data Fig 2c, top panel, PRSS2

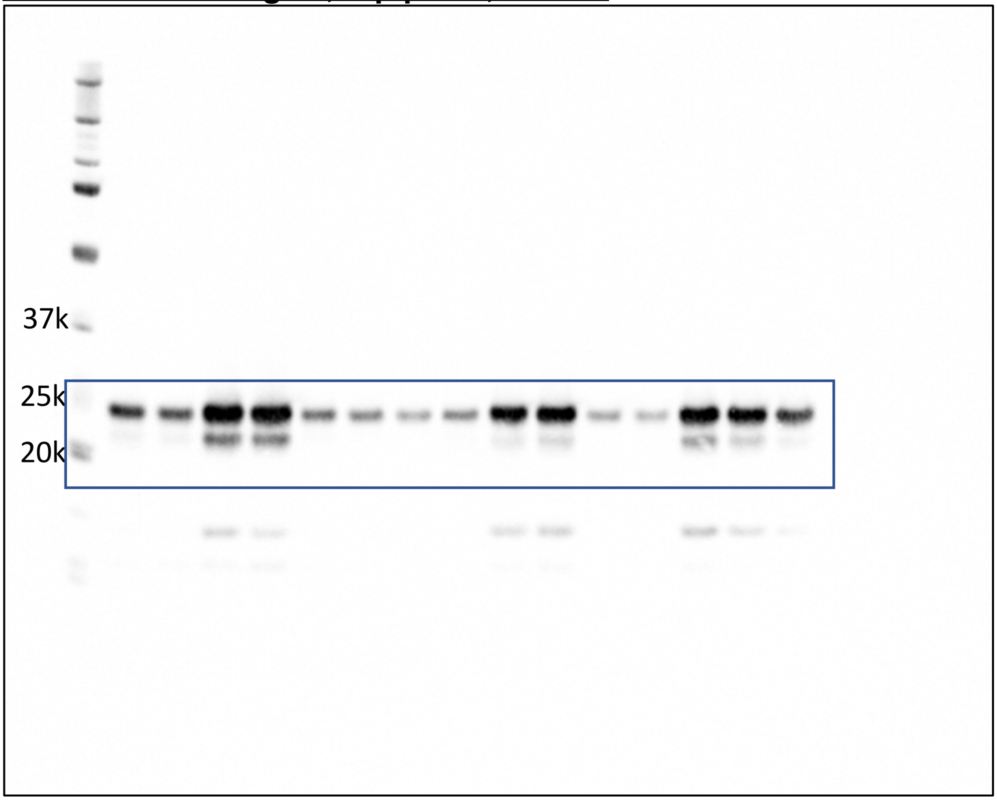

Extended Data Fig 2c, bottom panel, CELA3B, separate membrane

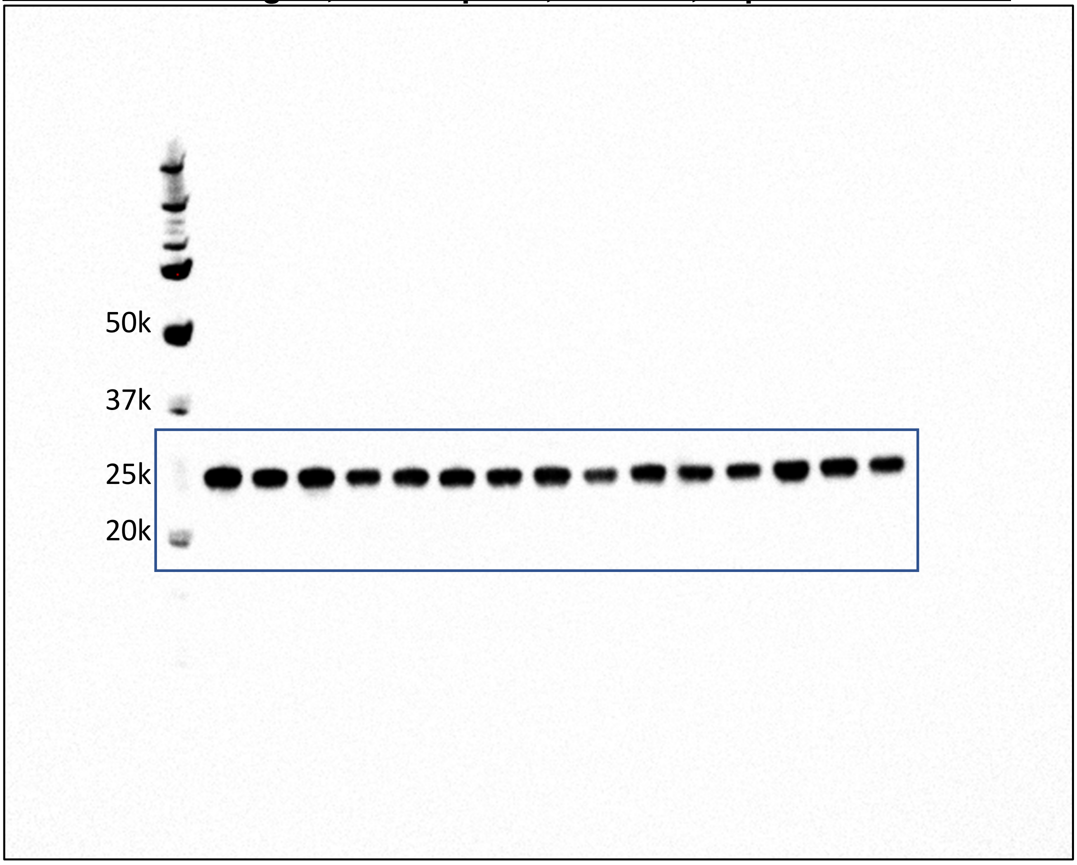

Extended Data Fig 3b, PRSS2

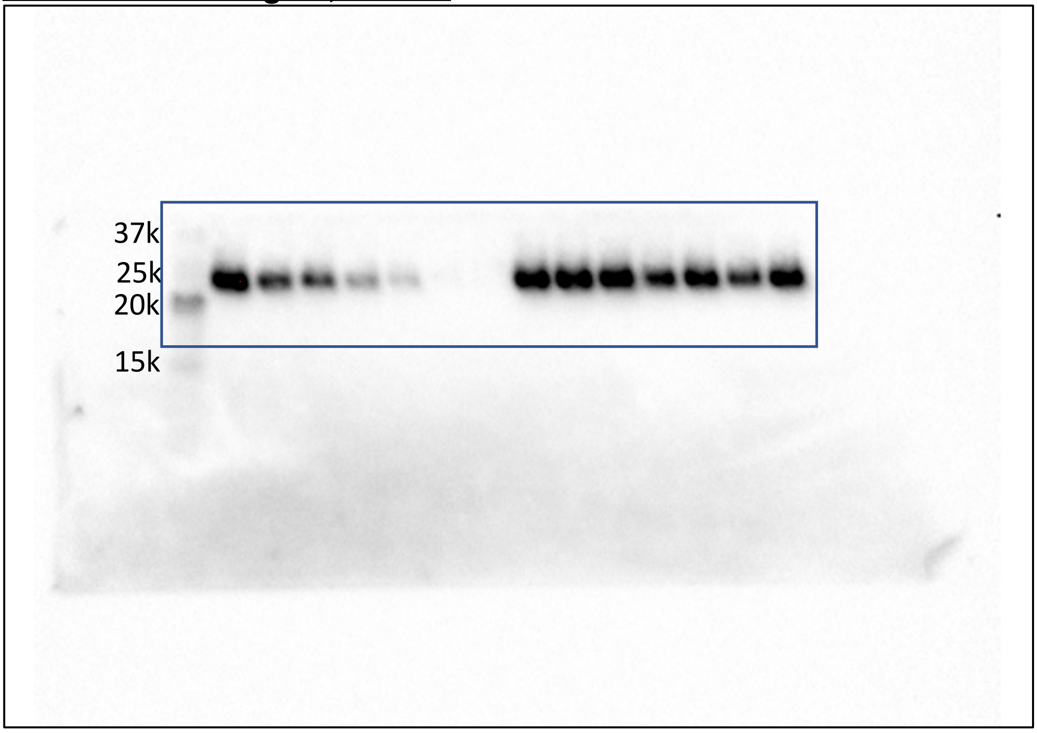

Extended Data Fig 3d, rmPRSS2

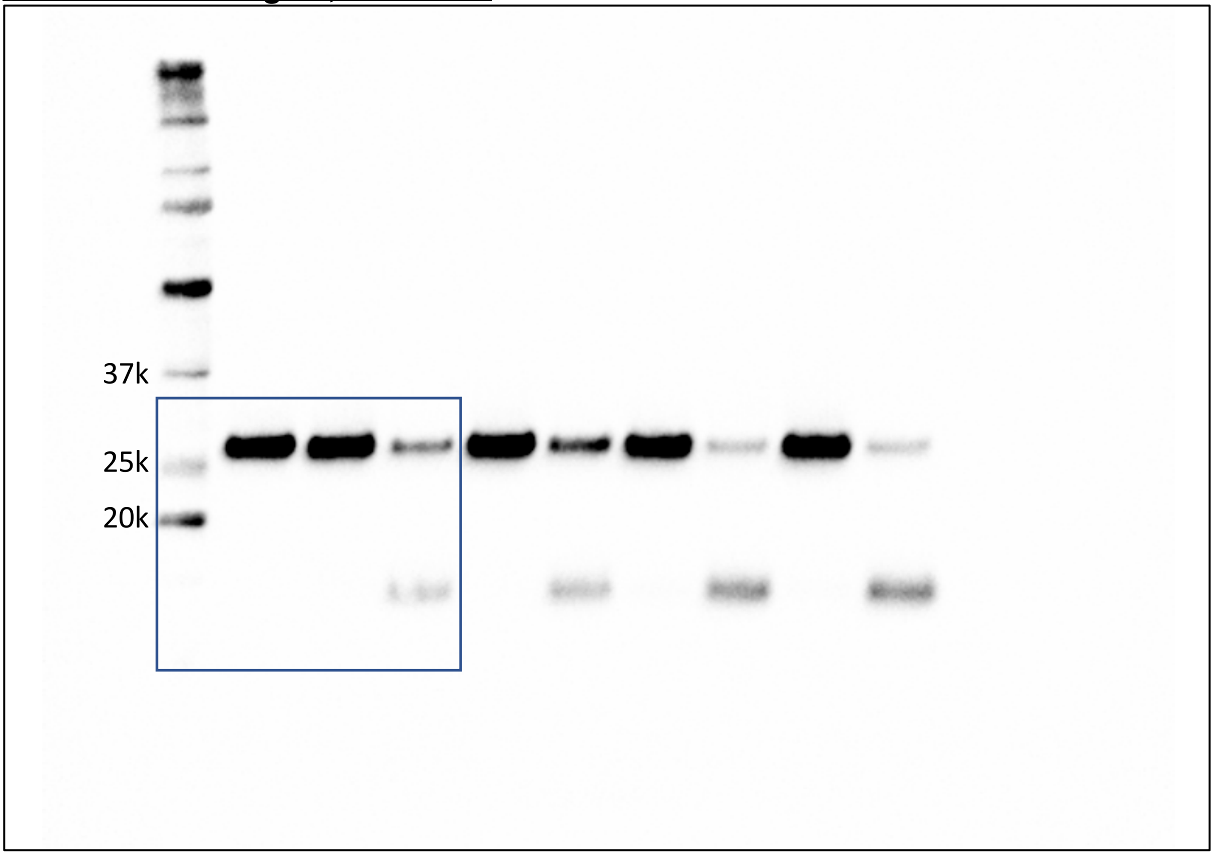

Extended Data Fig 3e, rmPRSS2

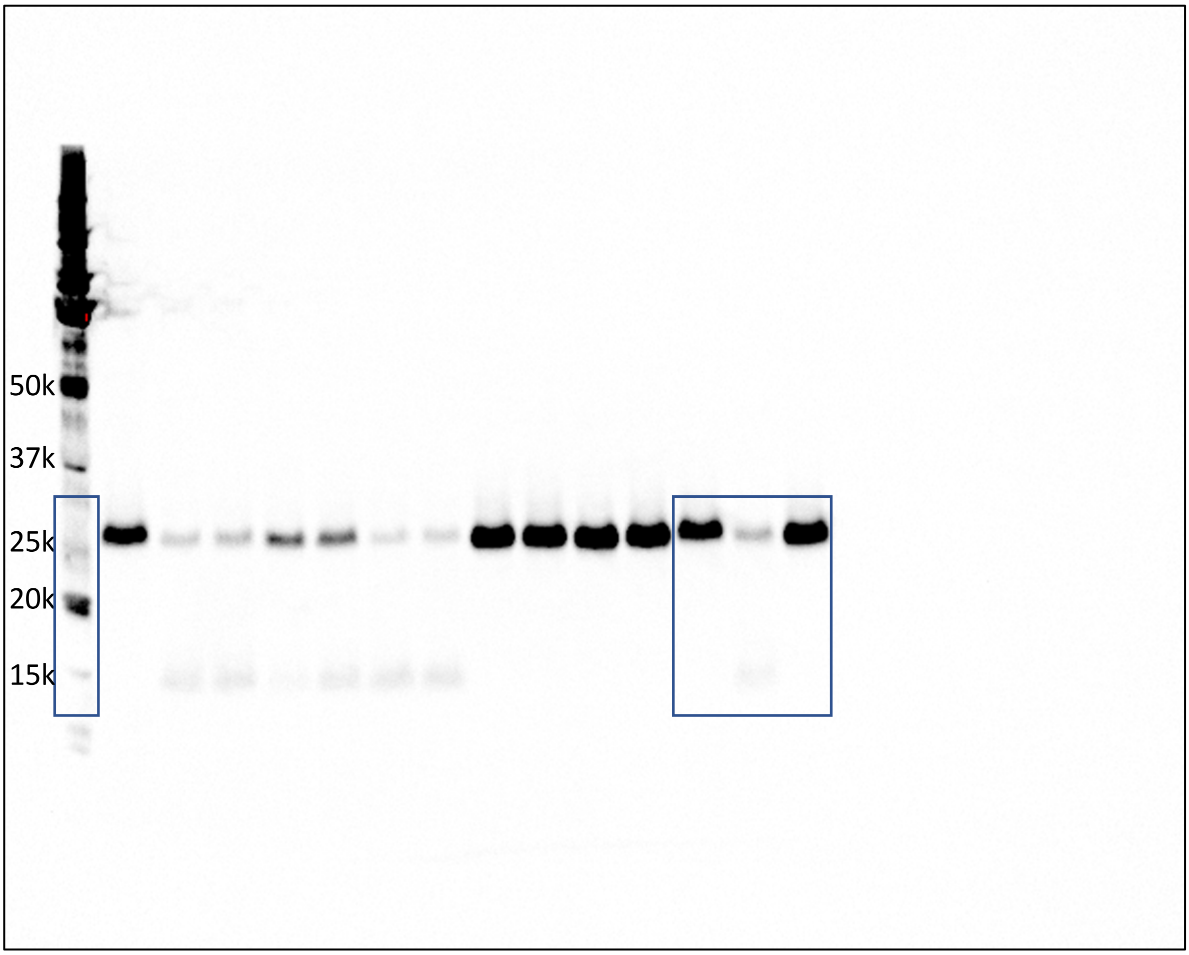

Extended Data Fig 3g, rmPRSS2

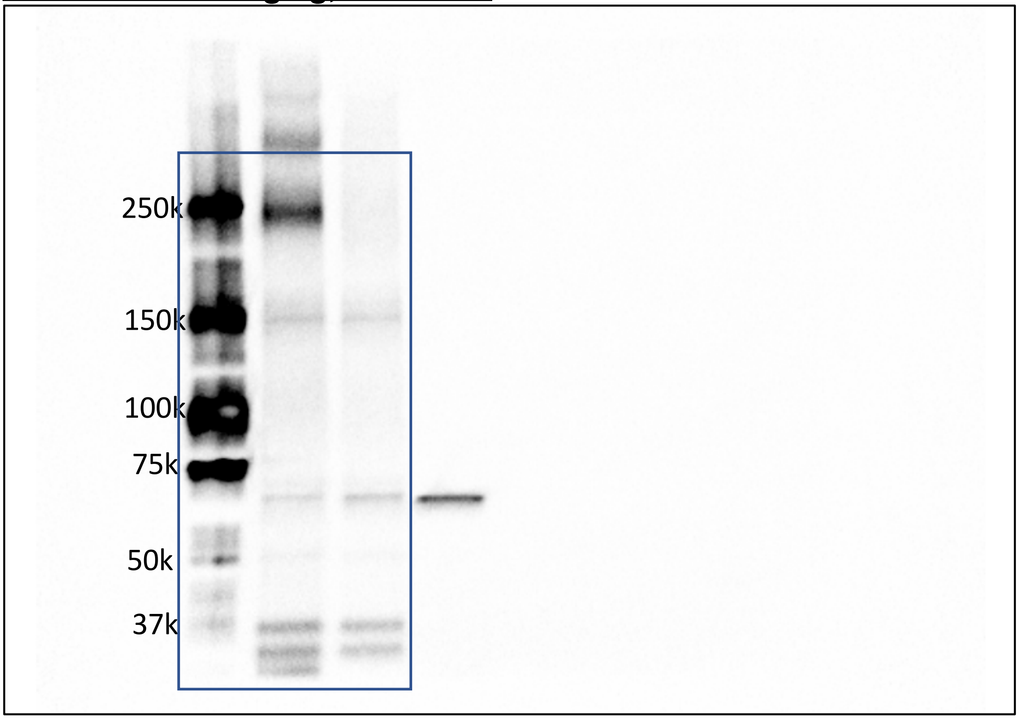

Extended Data Fig 4b, rmPRSS2

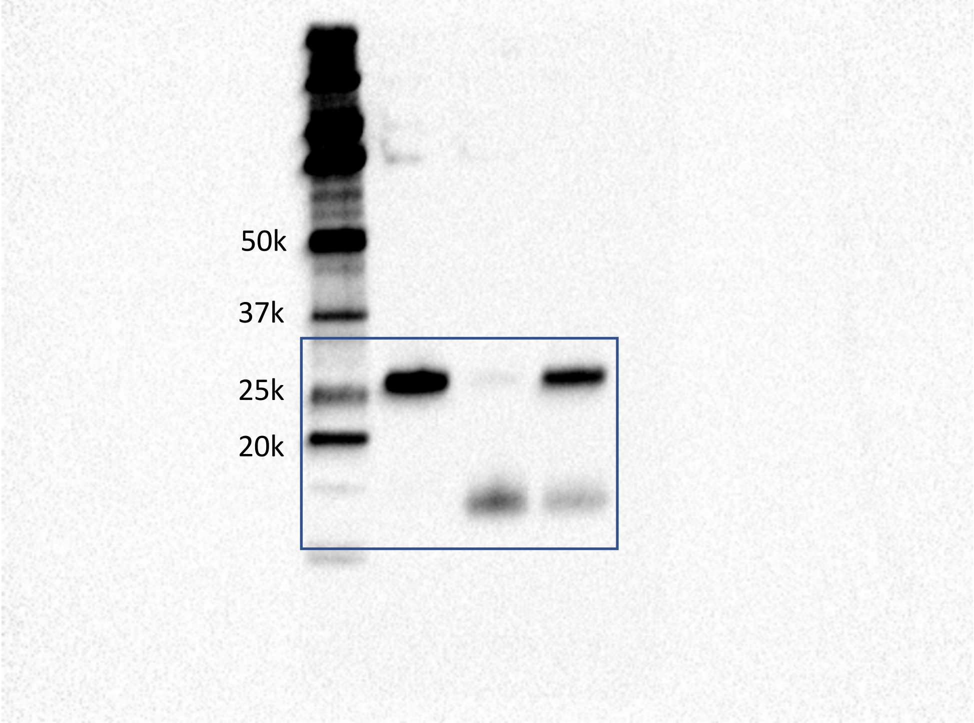

Extended Data Fig 4c, left panel

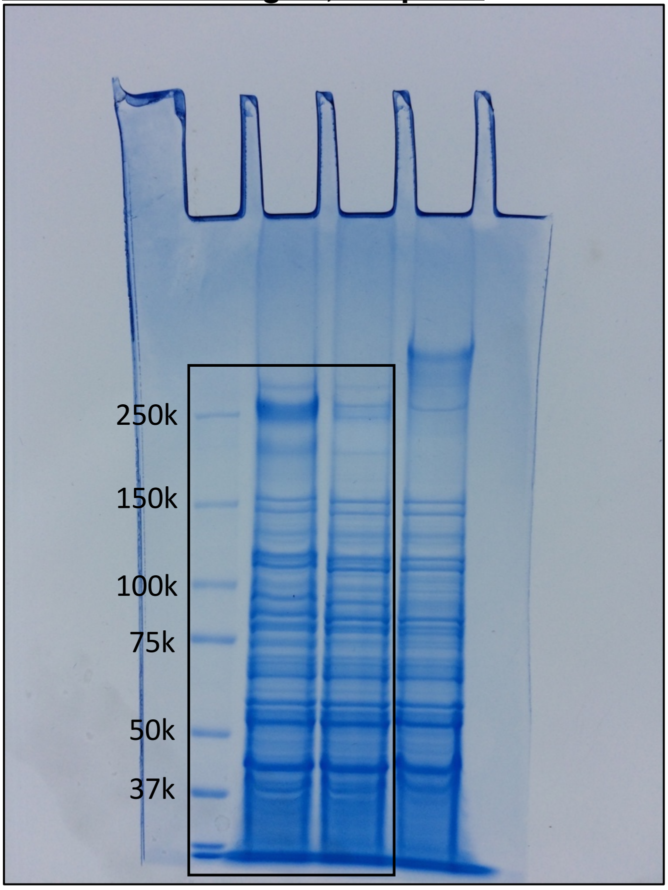

Extended Data Fig 4c, right panel

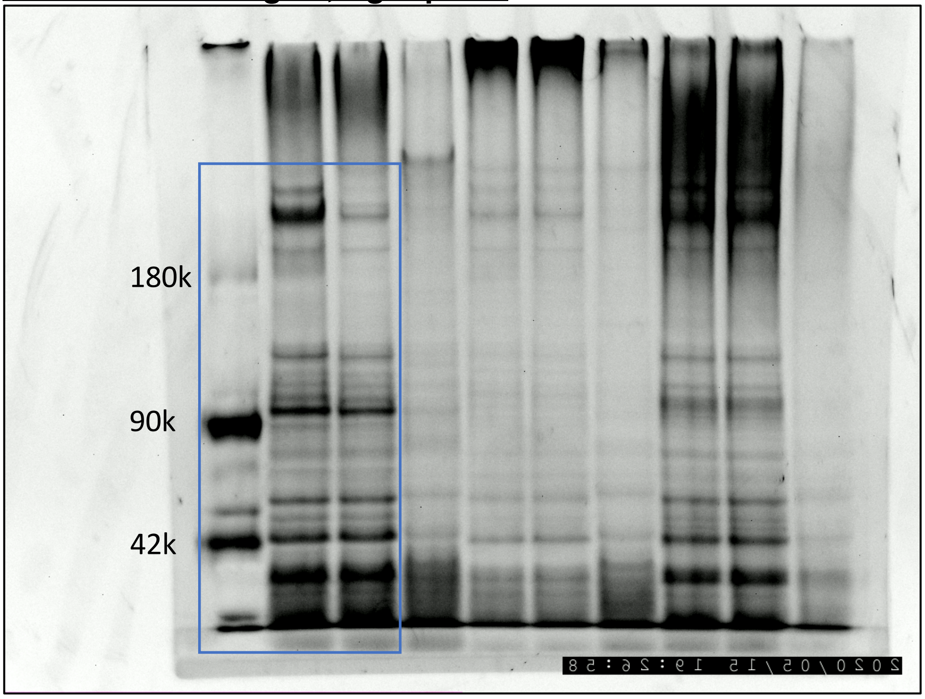

Extended Data Fig 4d

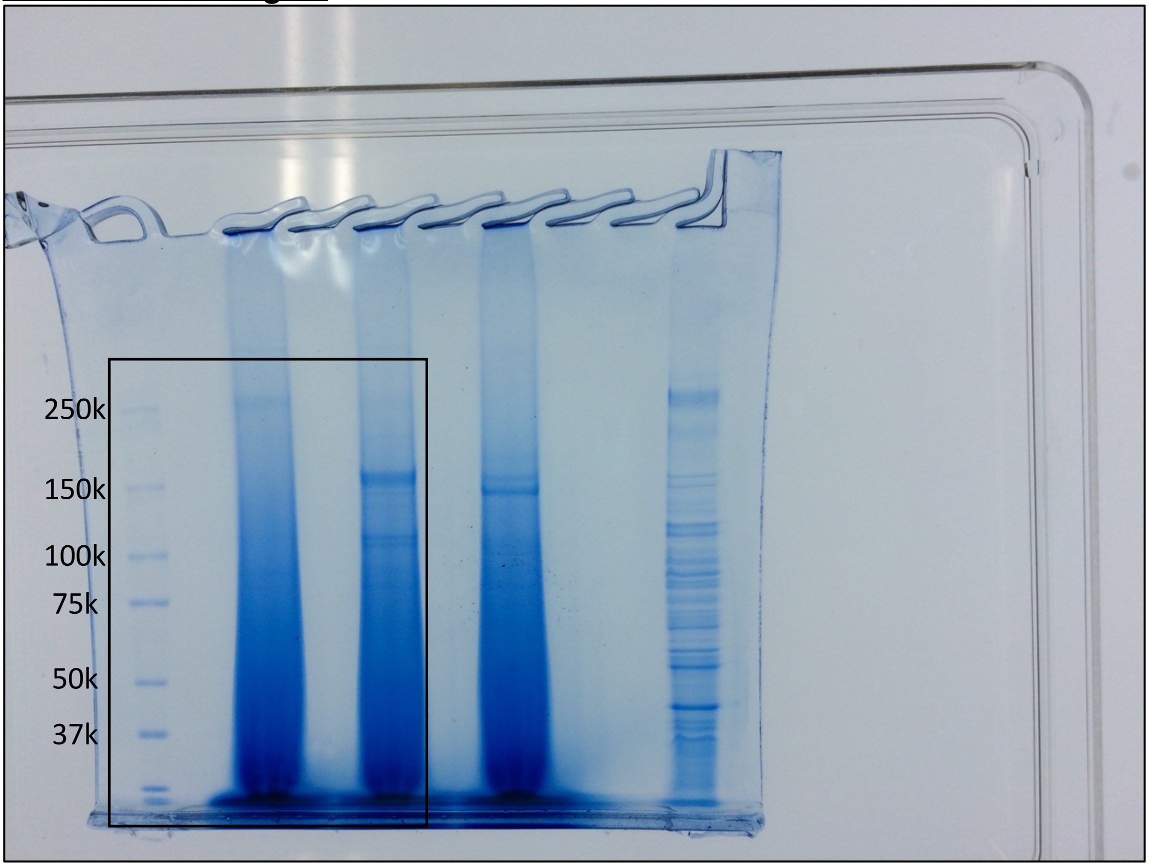

Extended Data Fig 5b top left panel, WecA mutant

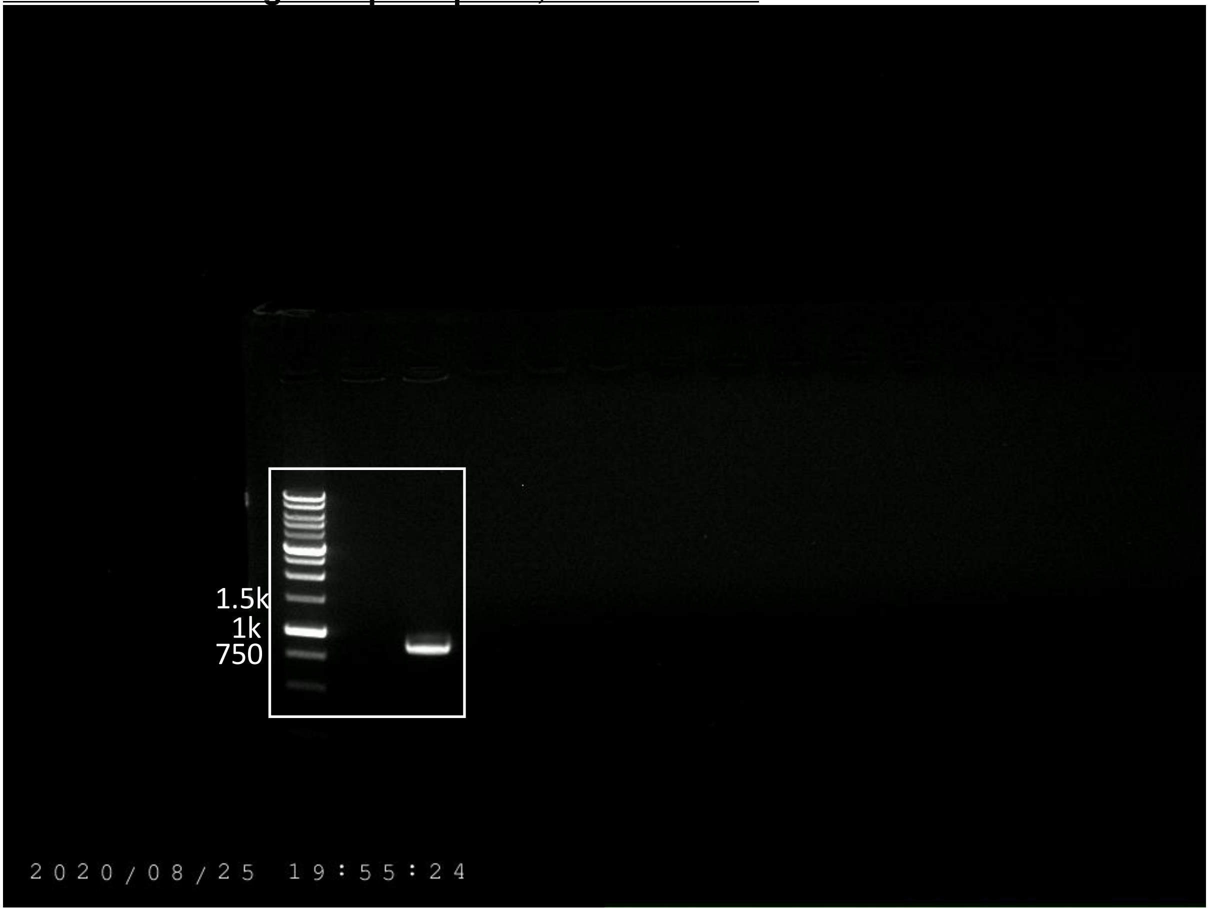

Extended Data Fig 5b, bottom left panel, PorU mutant

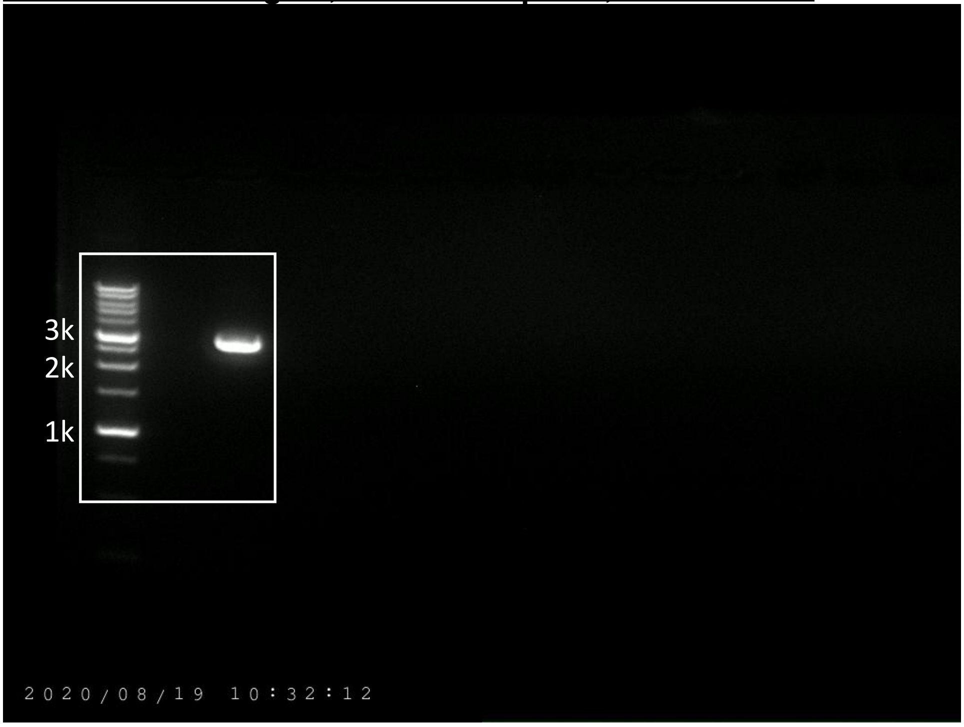

Extended Data Fig 5b, bottom right panel, 00503-00508 mutants

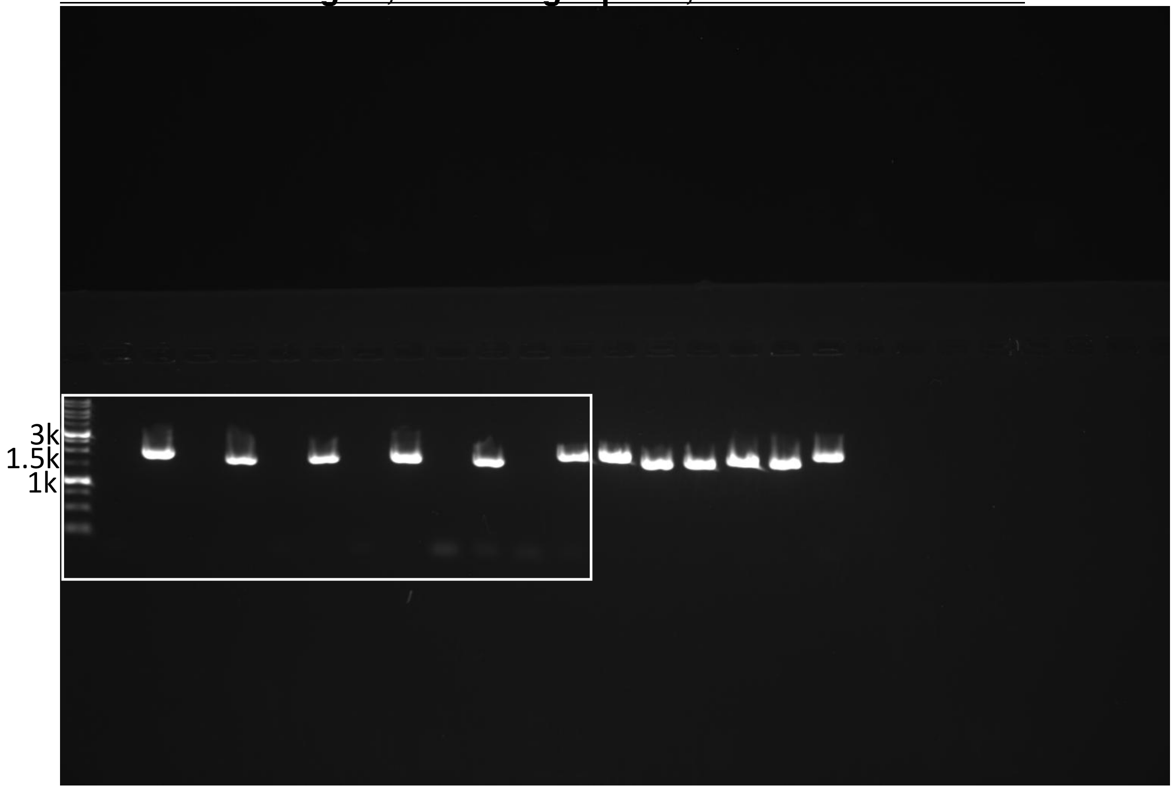

Extended Data Fig 5b, top and middle right panels, other mutants

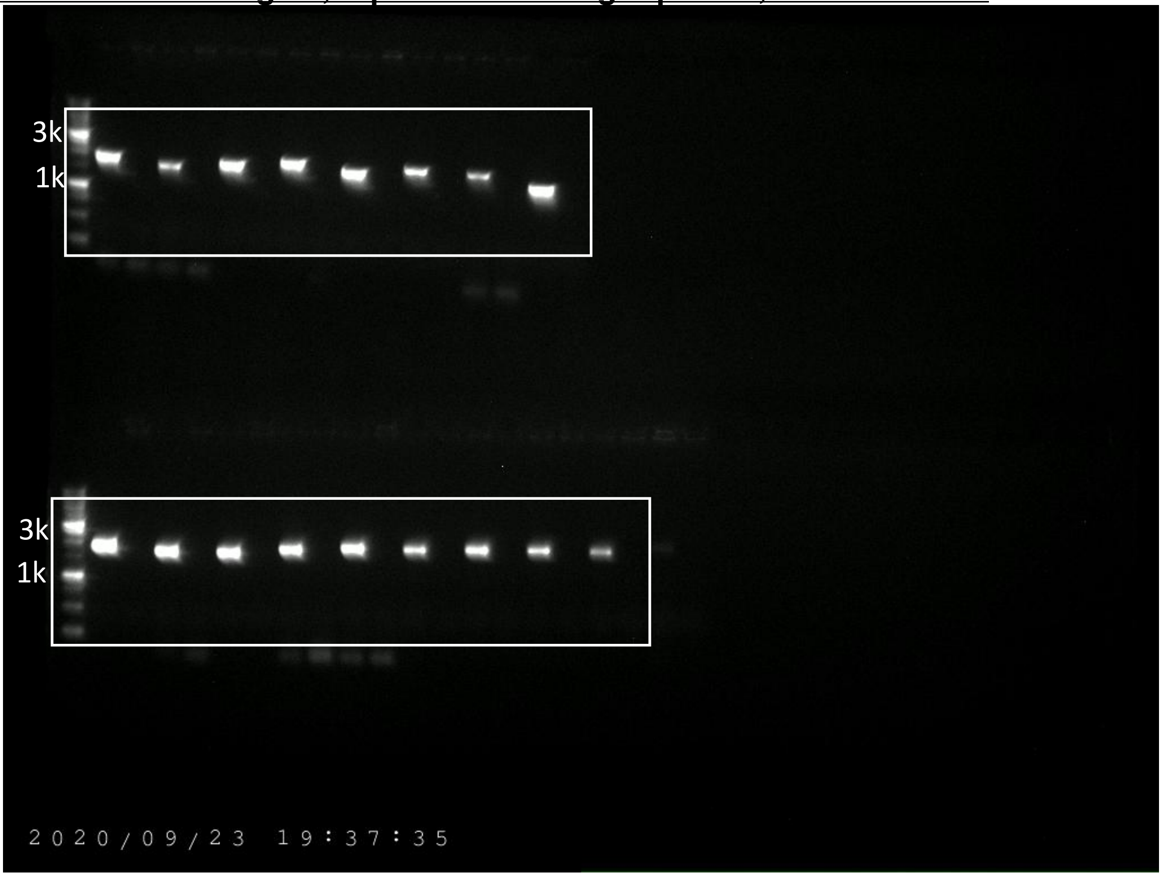

Extended Data Fig 6a, top panel, ∆03048-03053

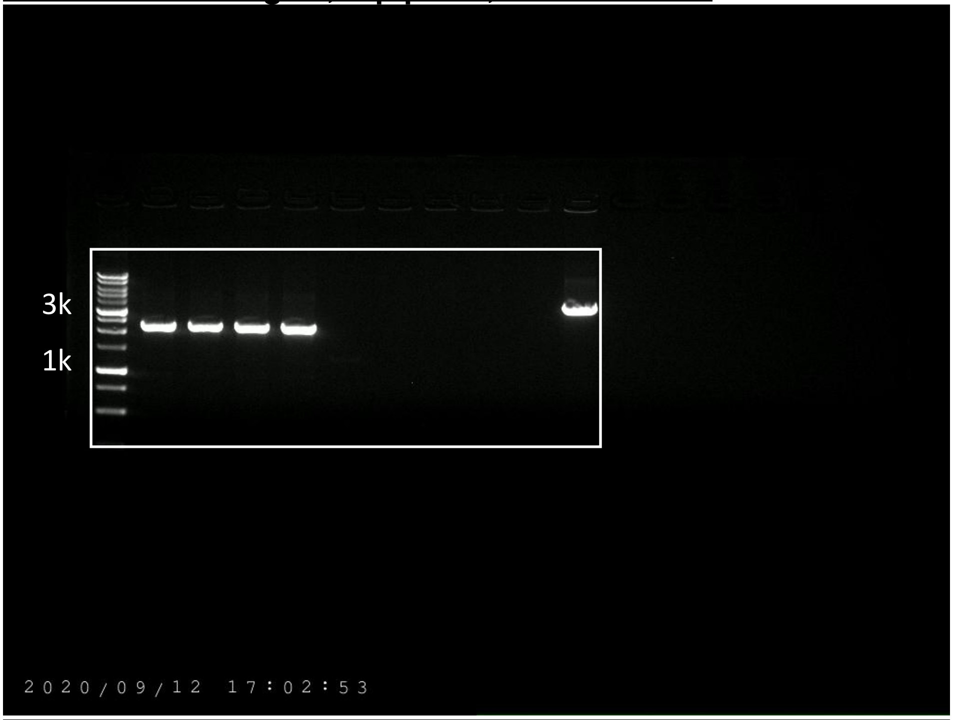

Extended Data Fig 6a, middle and bottom panels, Δ00502, Δ00509

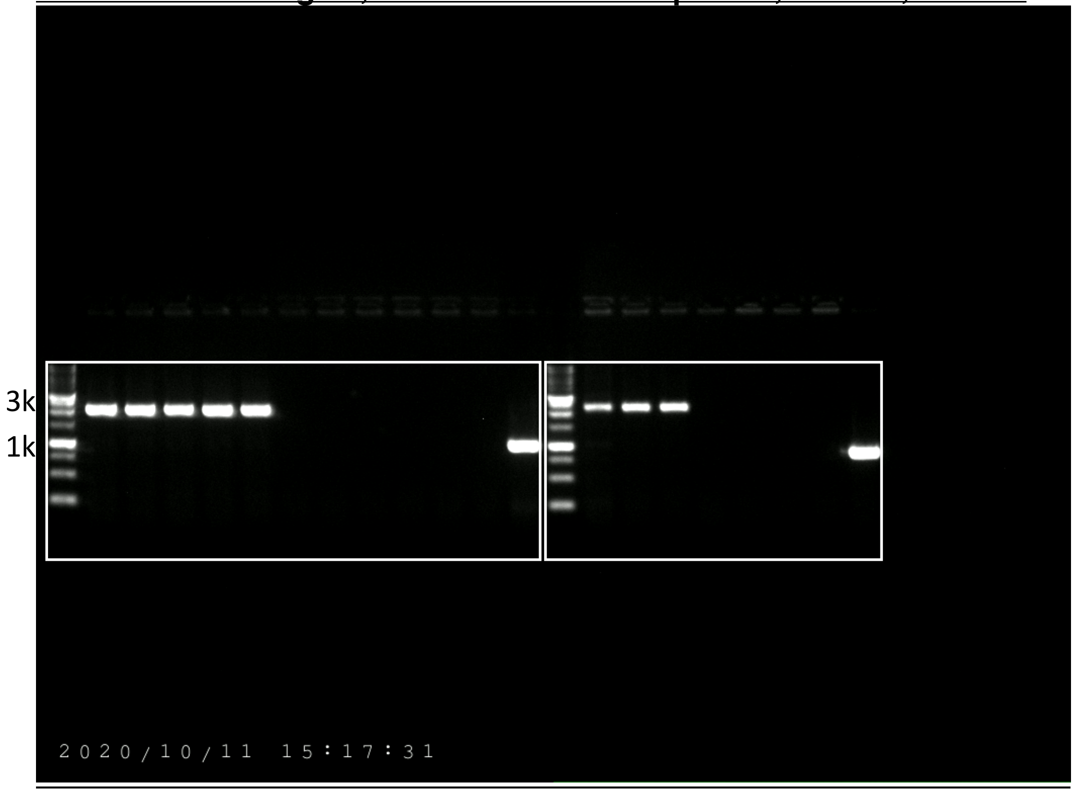

Extended Data Fig 6b rmPRSS2

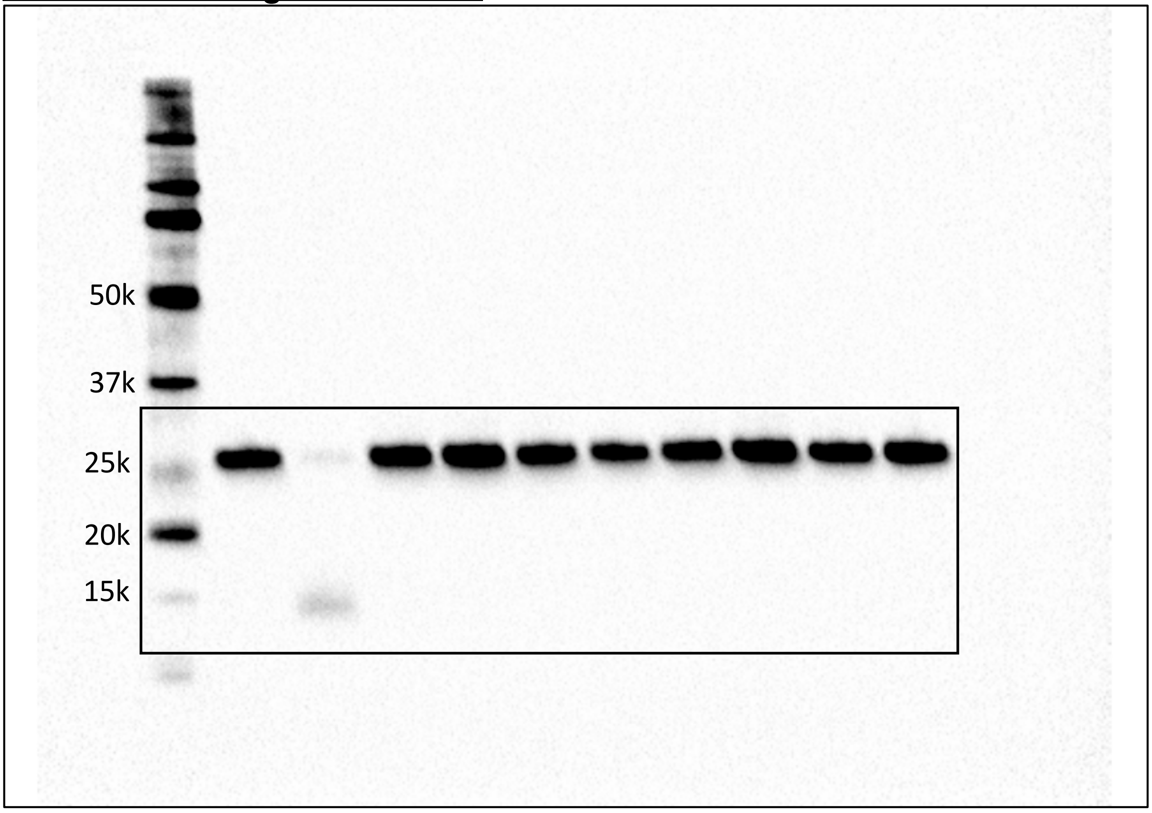

Extended Data Fig 6e rmPRSS2

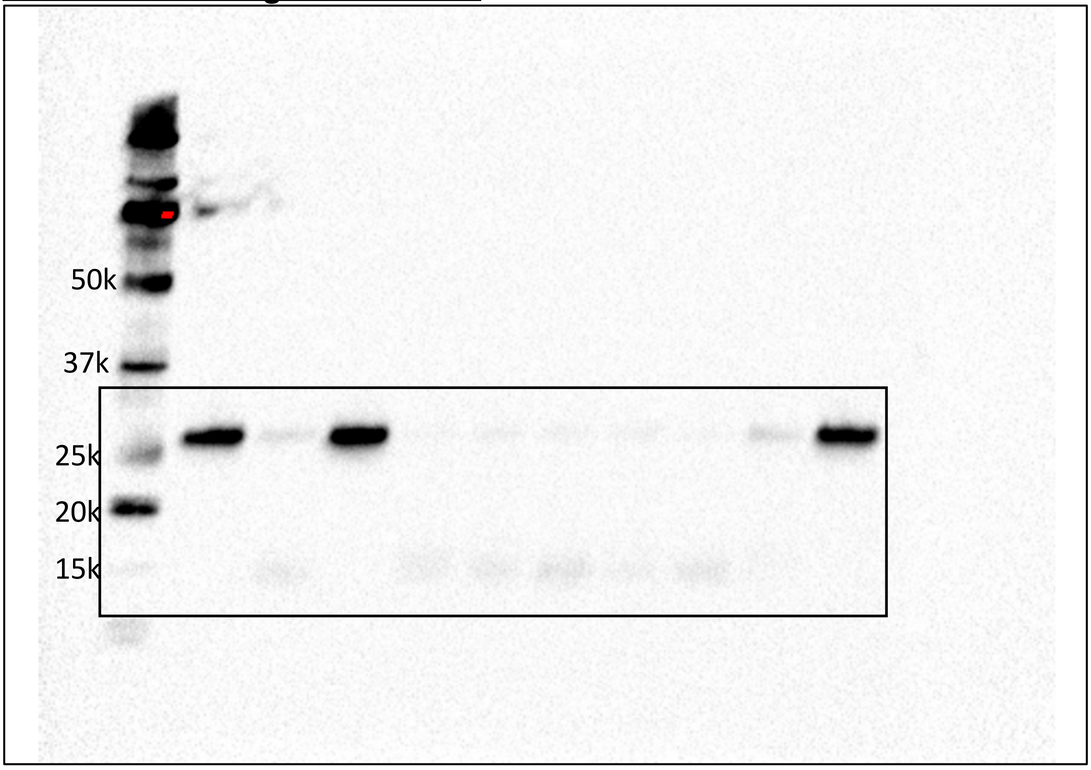

Extended Data Fig 7a

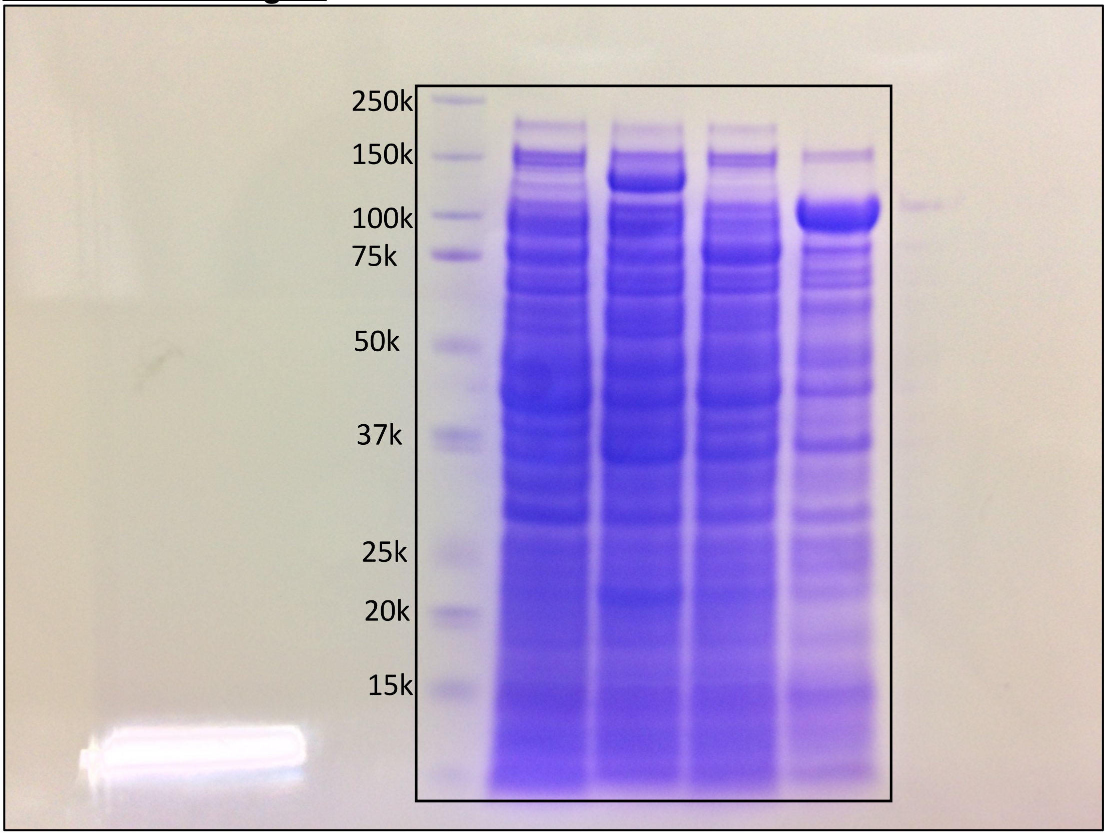

Extended Data Fig 7b

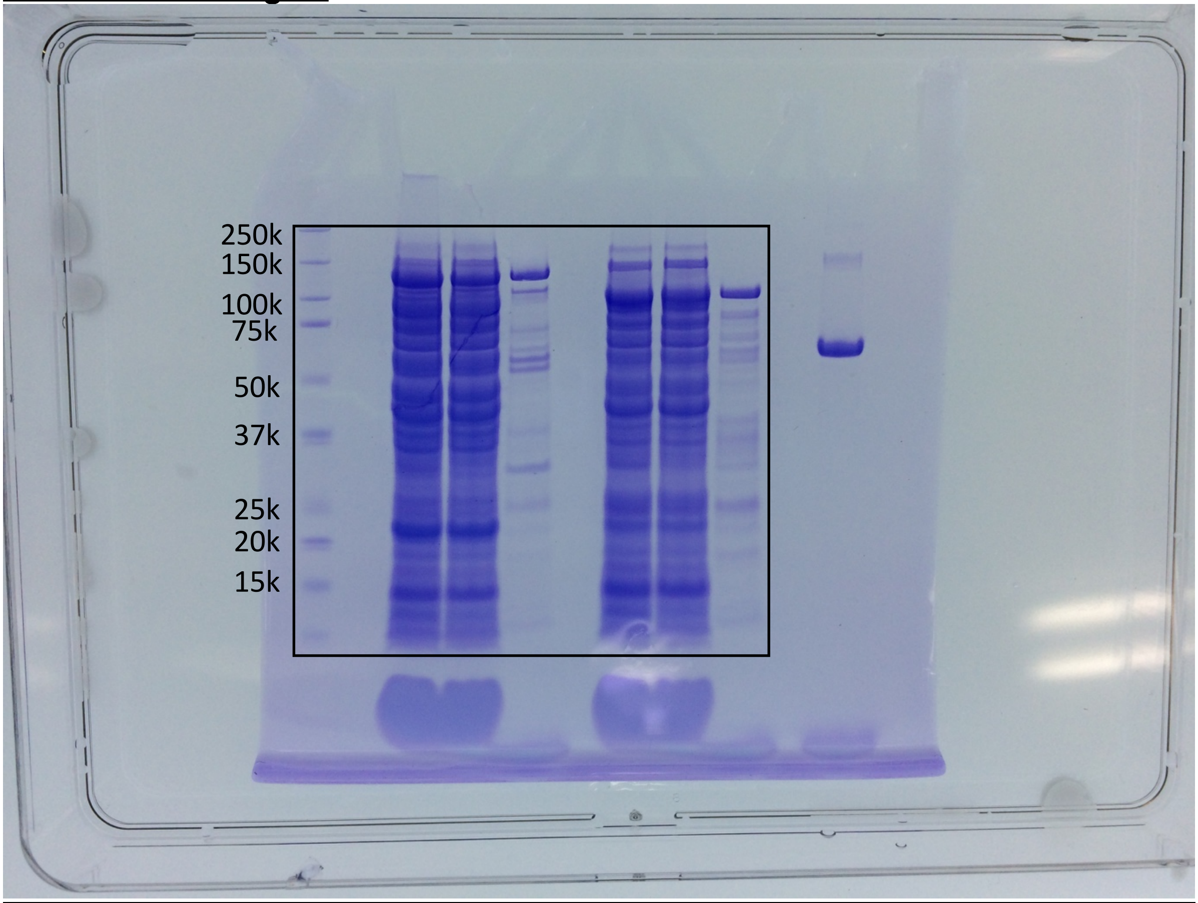

Extended Data Fig 7d, top panel, PRSS2

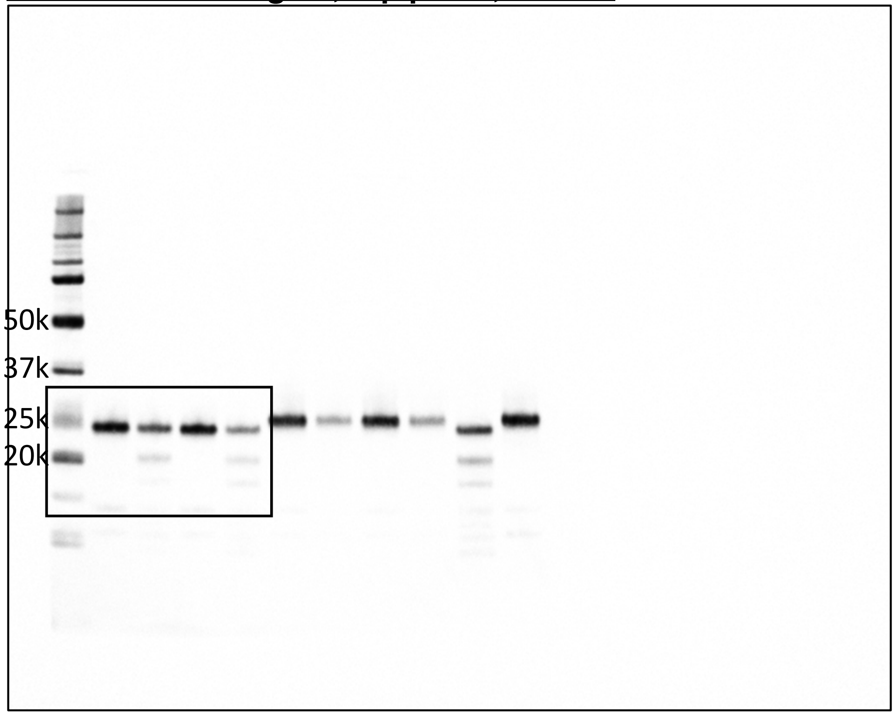

Extended Data Fig 7d, bottom panel, CELA3B (separate membrane)

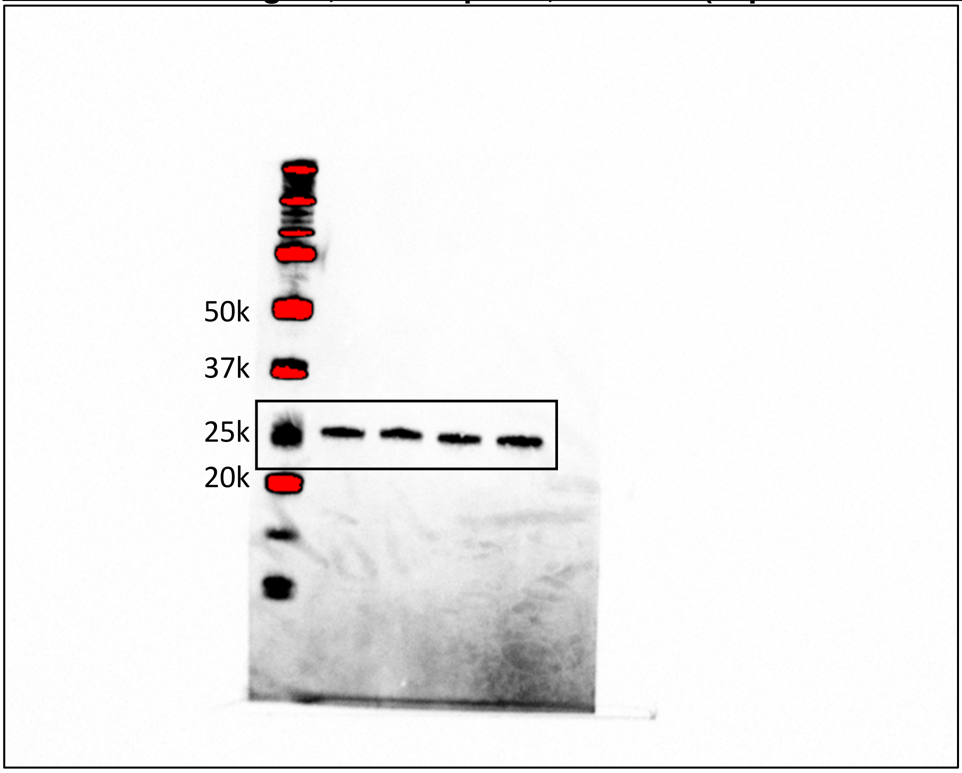

Extended Data Fig 7e, left panel

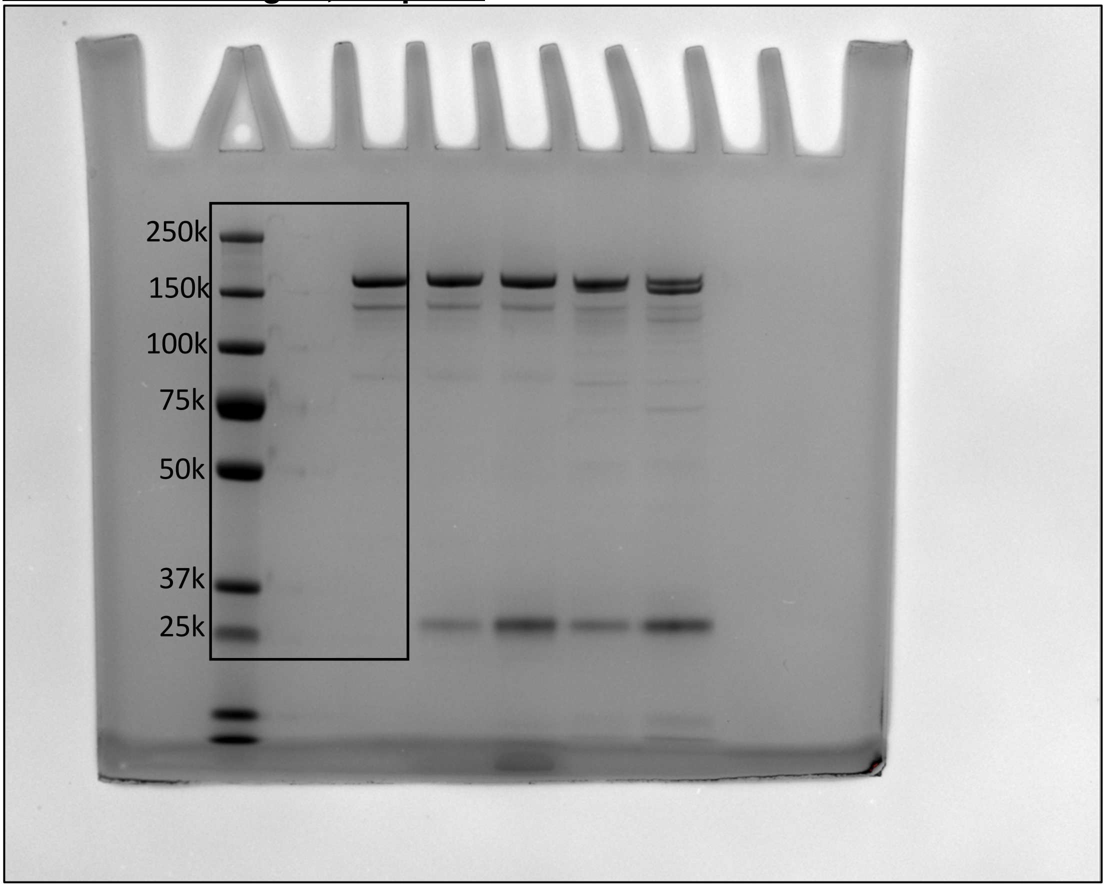

Extended Data Fig 7e, right panel

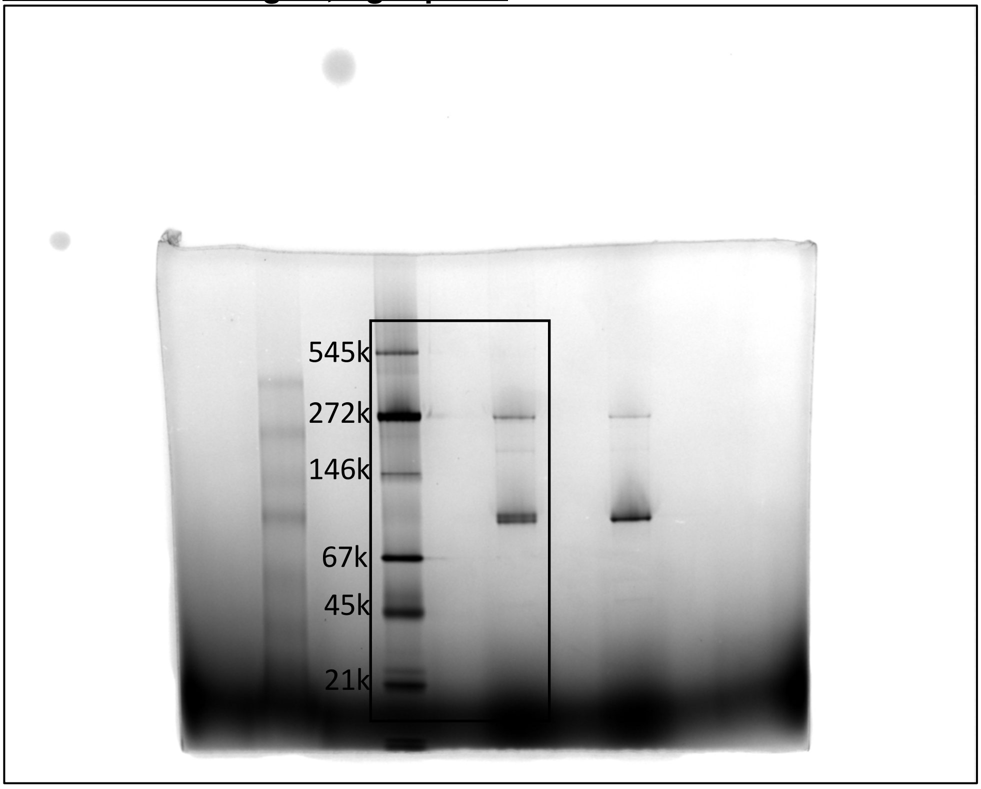

Extended Data Fig 7f, Left panel

Note: The marker used here is designed for SDS-PAGE-based chemiluminescent Western blot and does not reflect the actual molecular weight on a Native PAGE gel. It was used only for the purpose of aligning the individual bands between the gel and the blots.

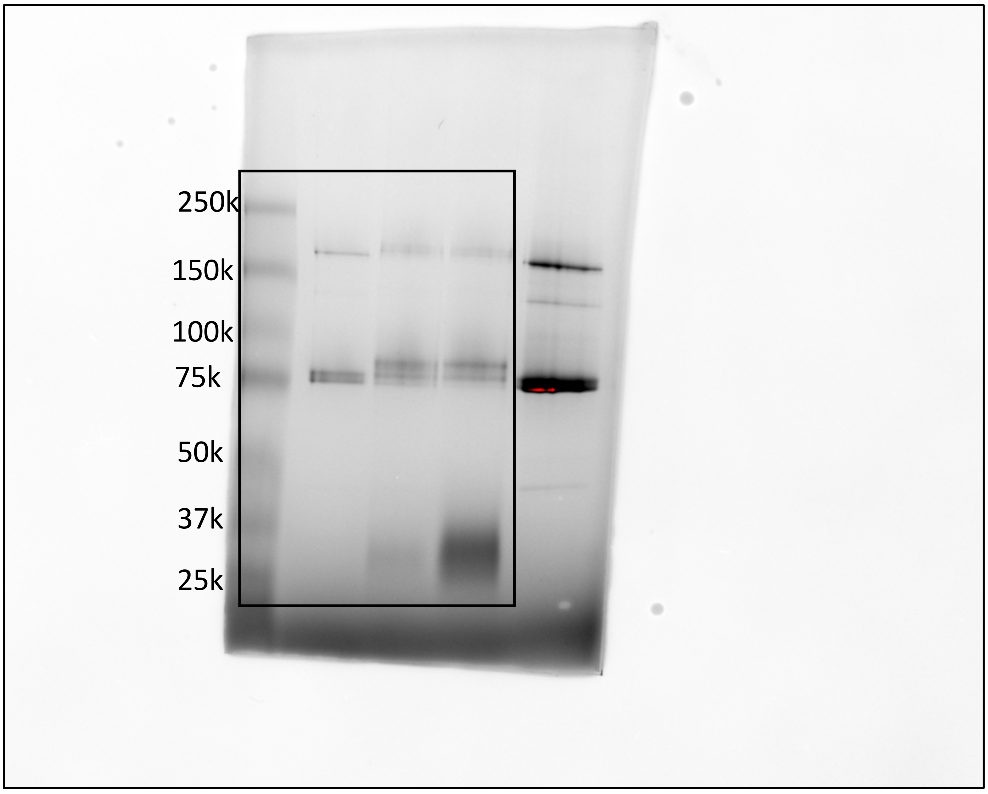

Extended Data Fig 7f, middle panel

Note: The marker used here is designed for SDS-PAGE-based chemiluminescent Western blot and does not reflect the actual molecular weight on a Native PAGE gel. It was used only for the purpose of aligning the individual bands between the gel and the blots.

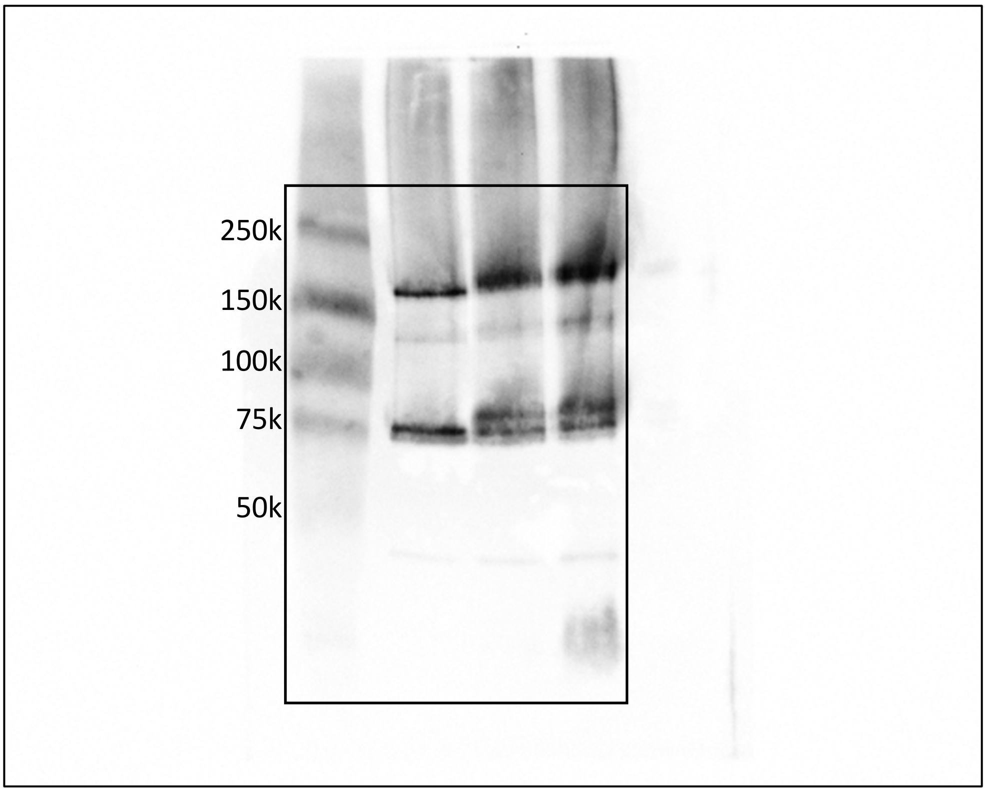

Extended Data Fig 7f, right panel

Note: The marker used here is designed for SDS-PAGE-based chemiluminescent Western blot and does not reflect the actual molecular weight on a Native PAGE gel. It was used only for the purpose of aligning the individual bands between the gel and the blots.

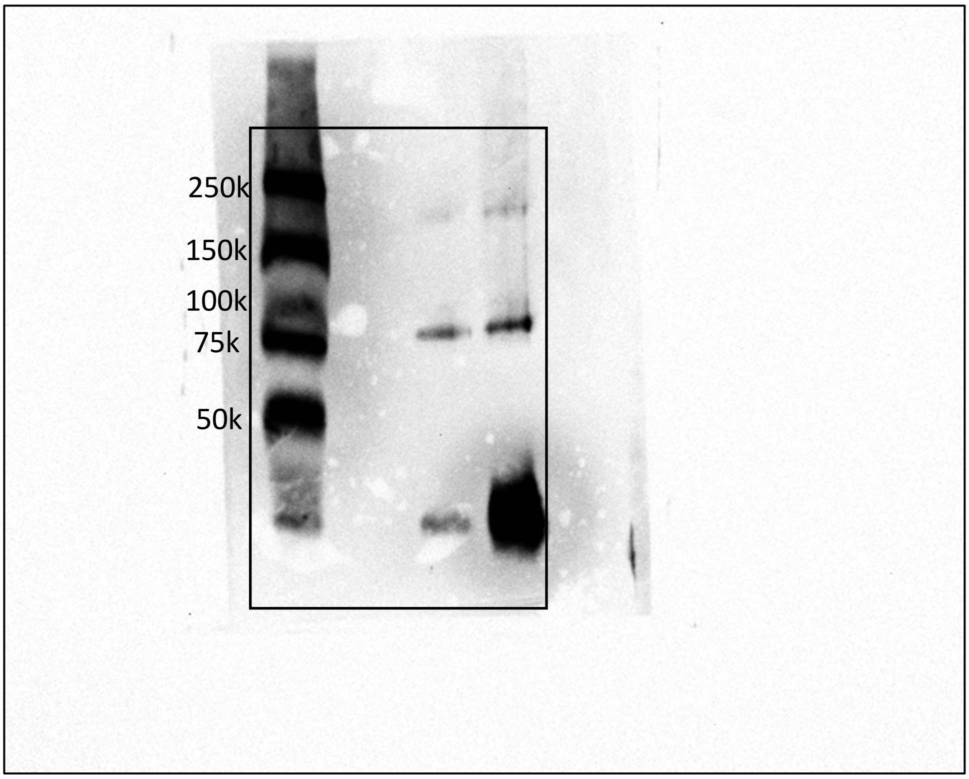

Extended Data Fig 7g

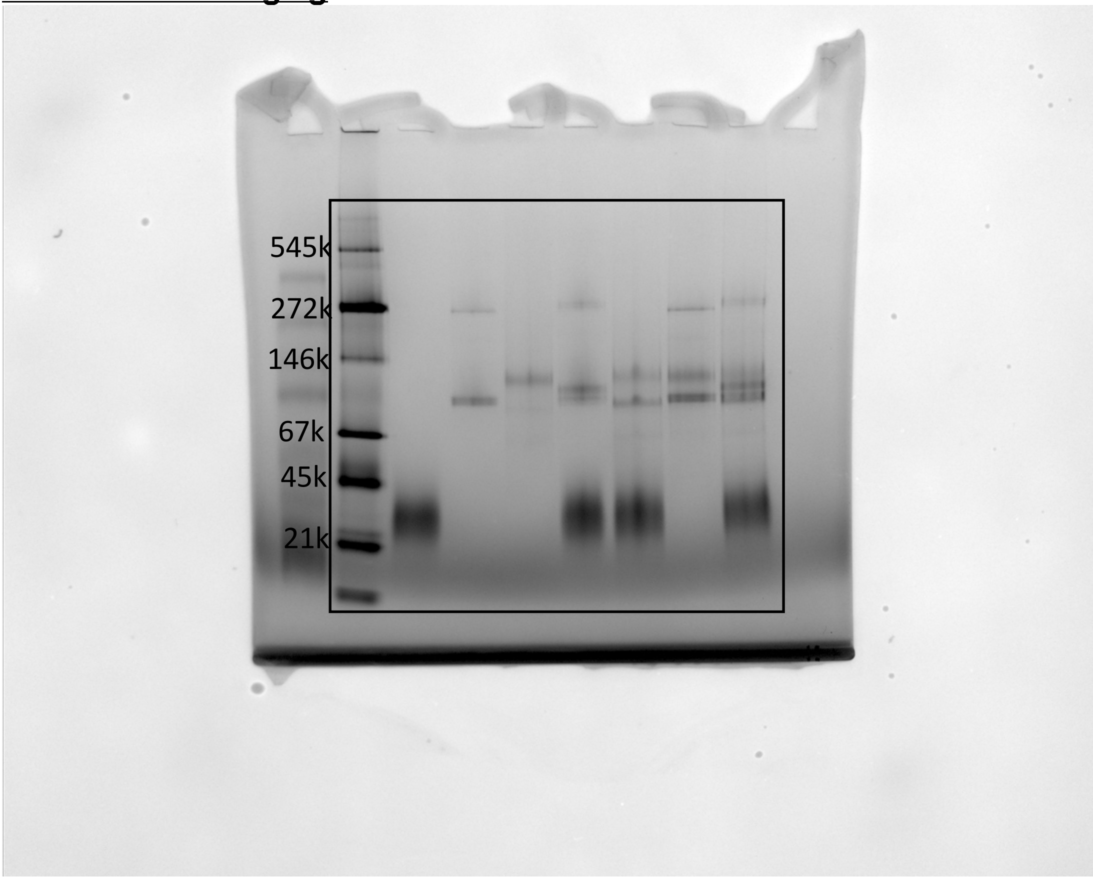

Extended Data Fig 9e

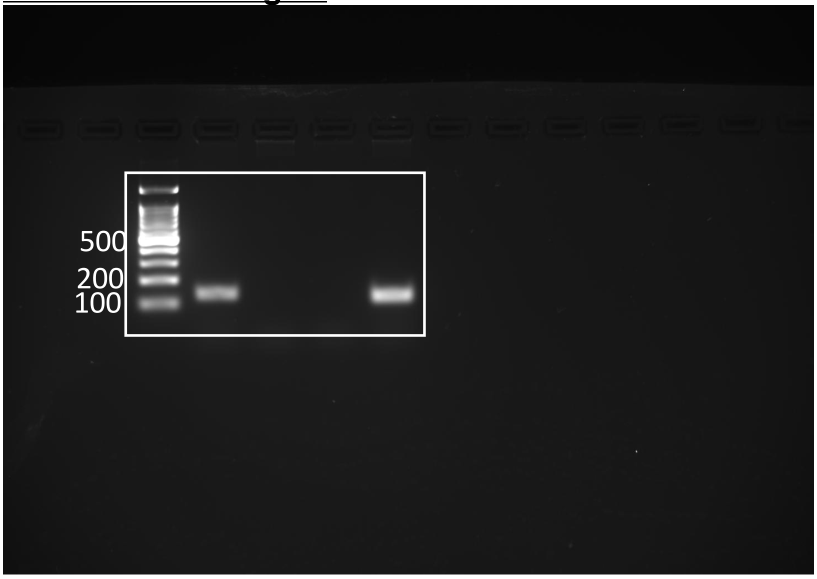

Extended Data Fig 10a, panel 1, both left and right, PRSS2 [same membrane with IgA (α chain)]

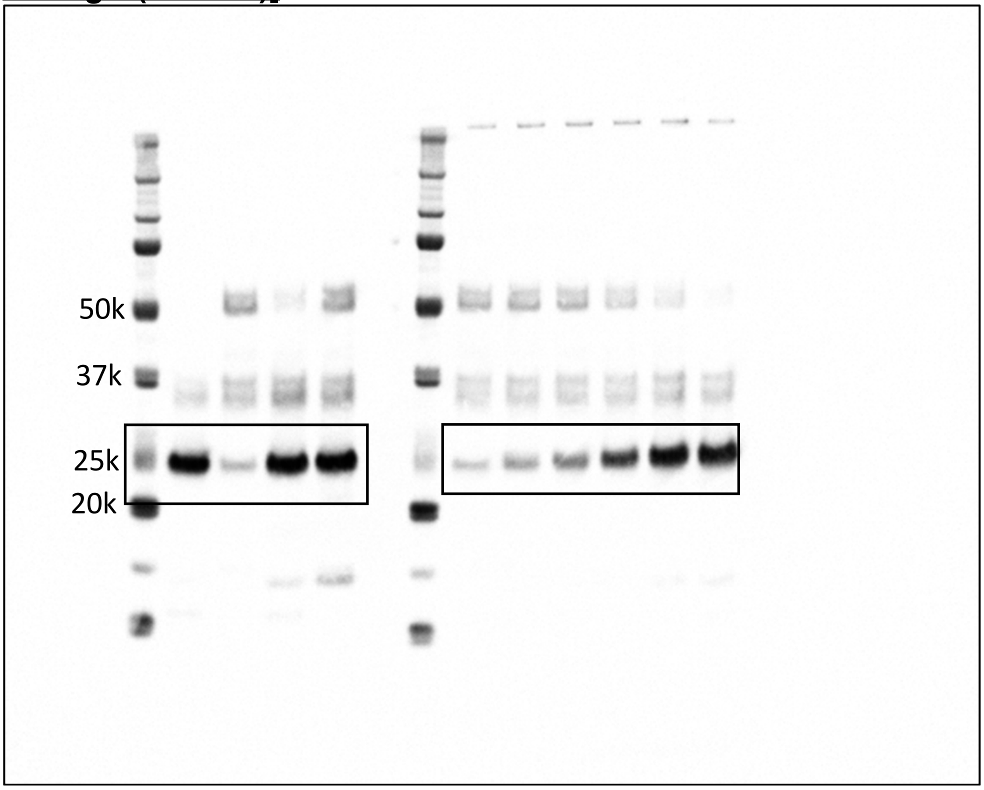

Extended Data Fig 10a, panel 2, both left and right, IgA (α chain)

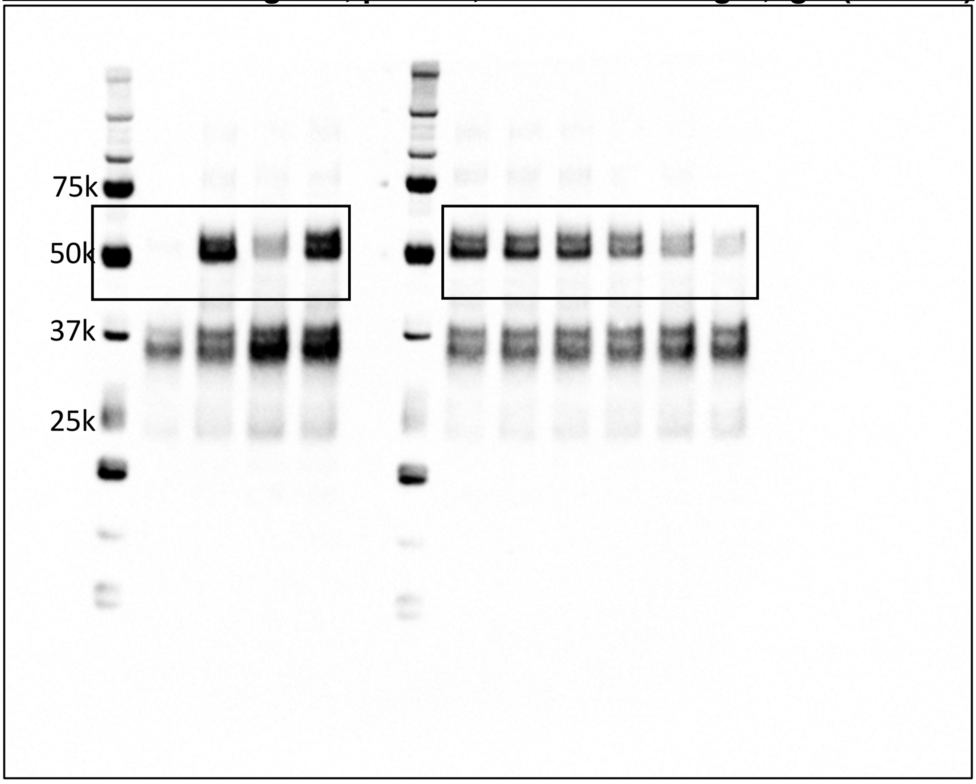

Extended Data Fig 10a, panel 3, both left and right, κ chain

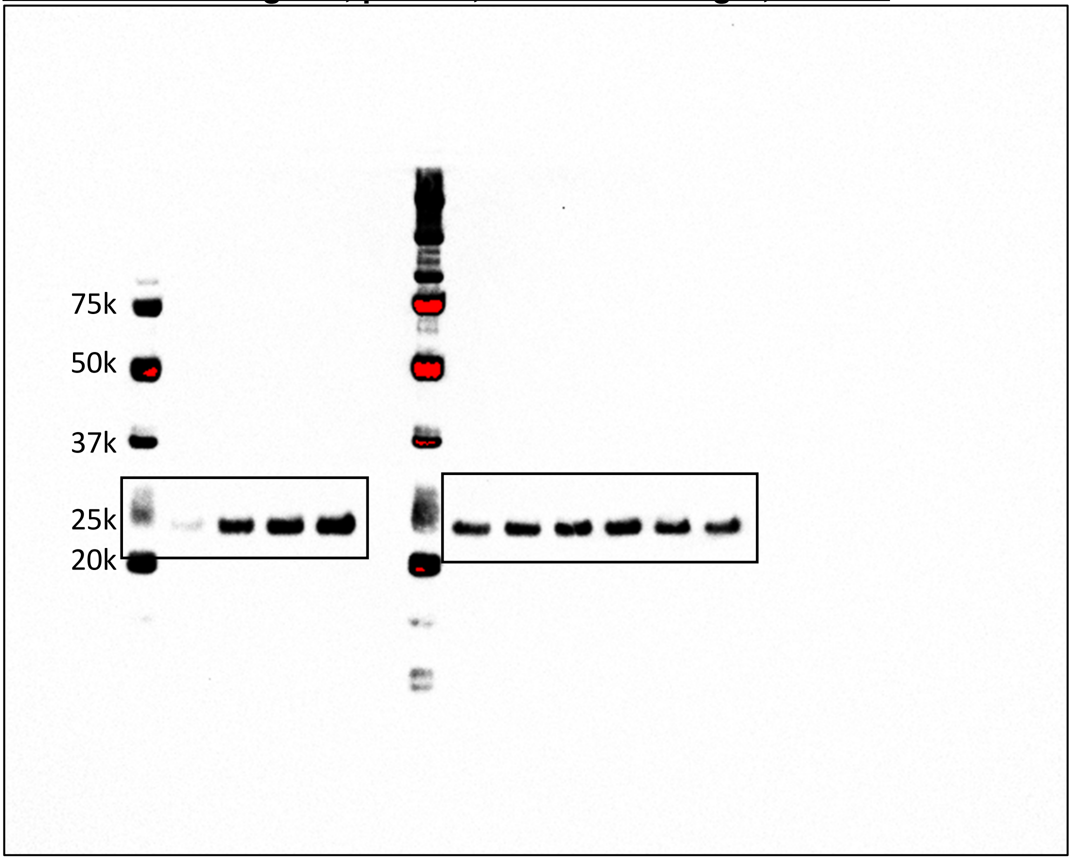

Extended Data Fig 10a, panel 4, left, CELA3B (separate membrane)

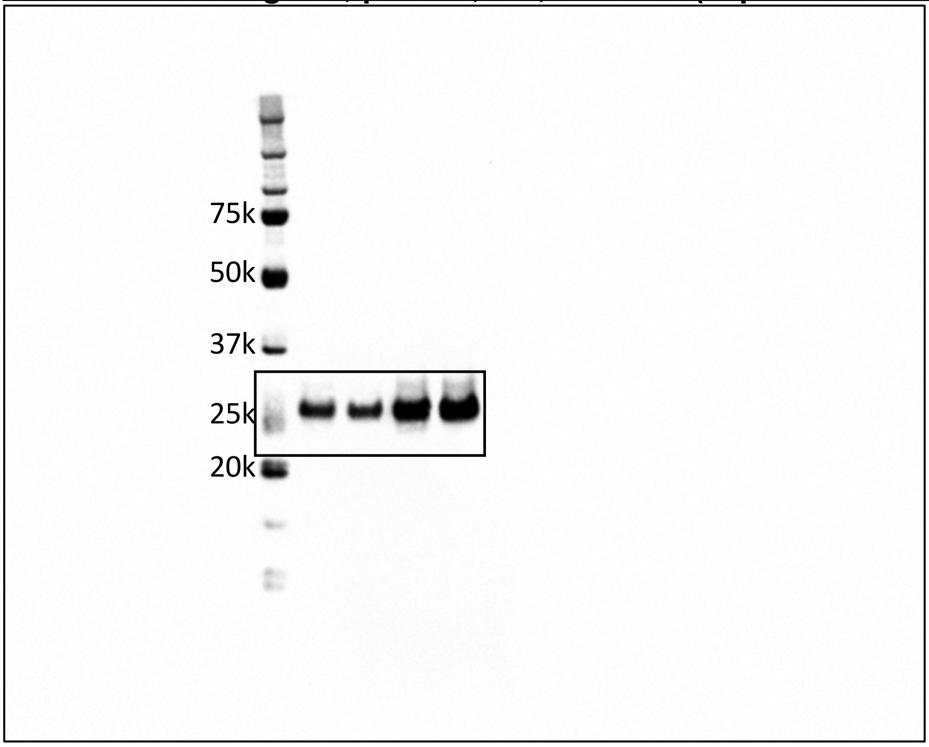

Extended Data Fig 10a, panel 4, right, CELA3B (separate membrane)

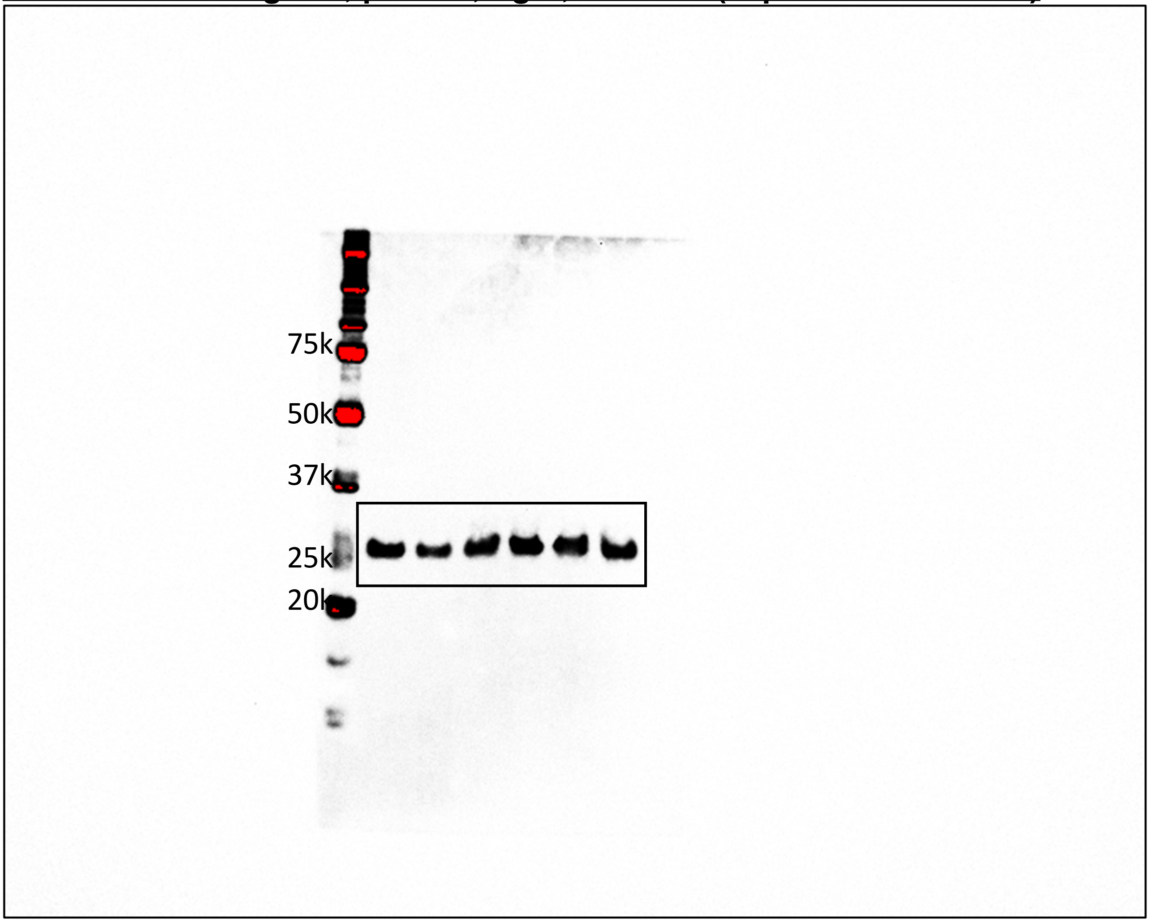

Extended Data Fig 10e, panel 1, PRSS2 [same membrane with total IgA (α chain)]

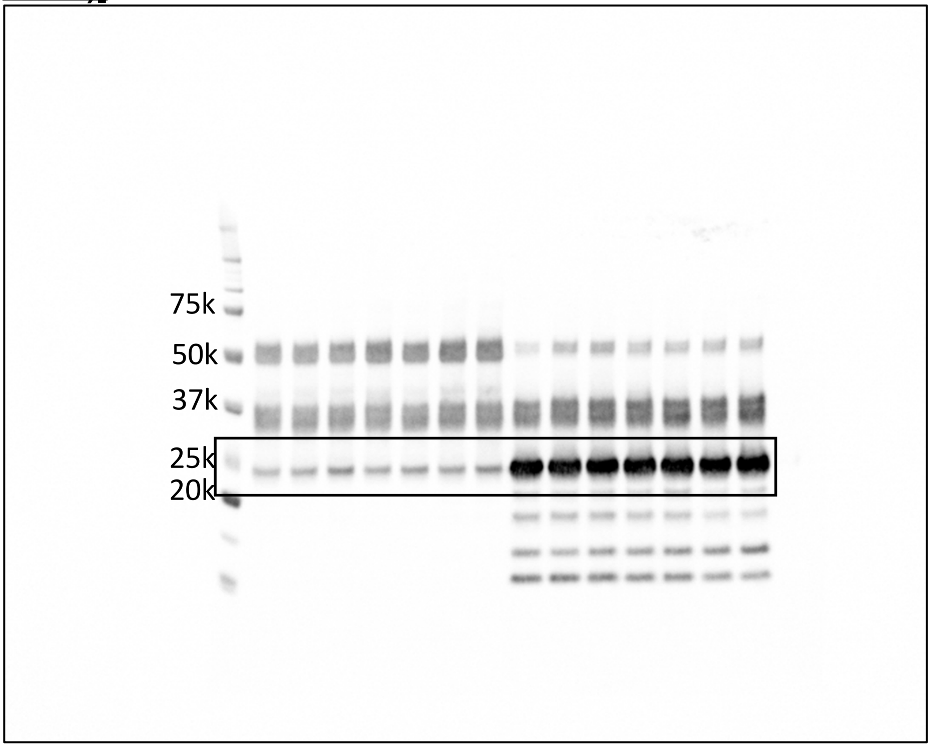

Extended Data Fig 10e, panel 2, total IgA (α chain)

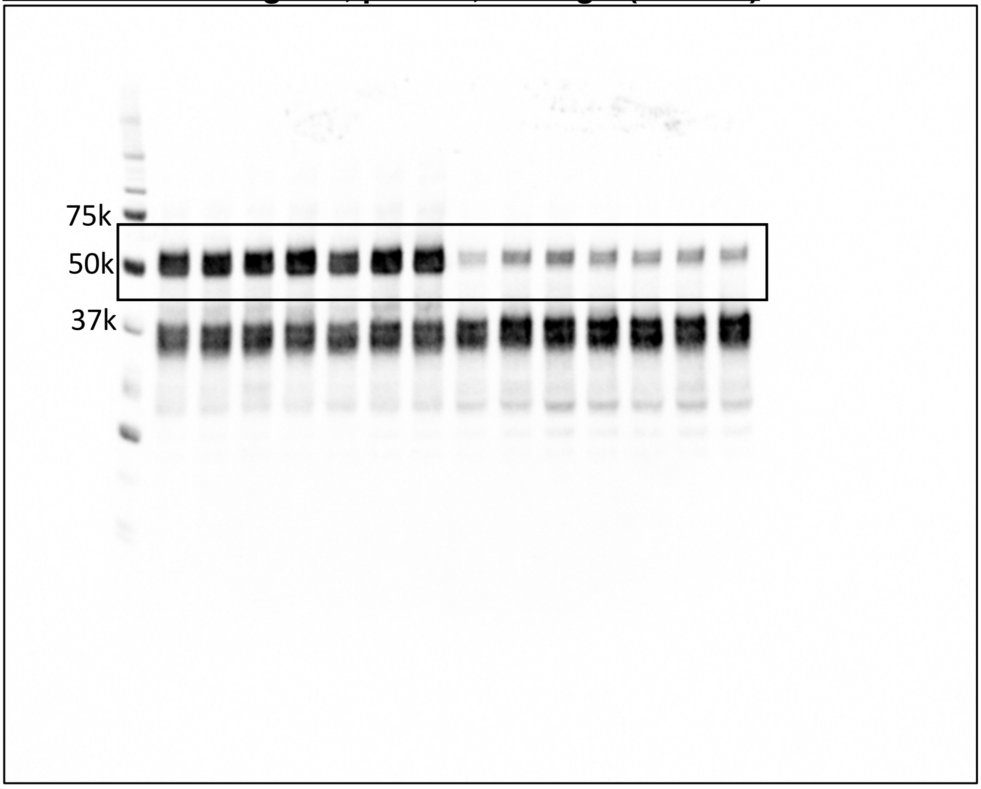

Extended Data Fig 10e, panel 3, anti-C. rodentium IgA (α chain)

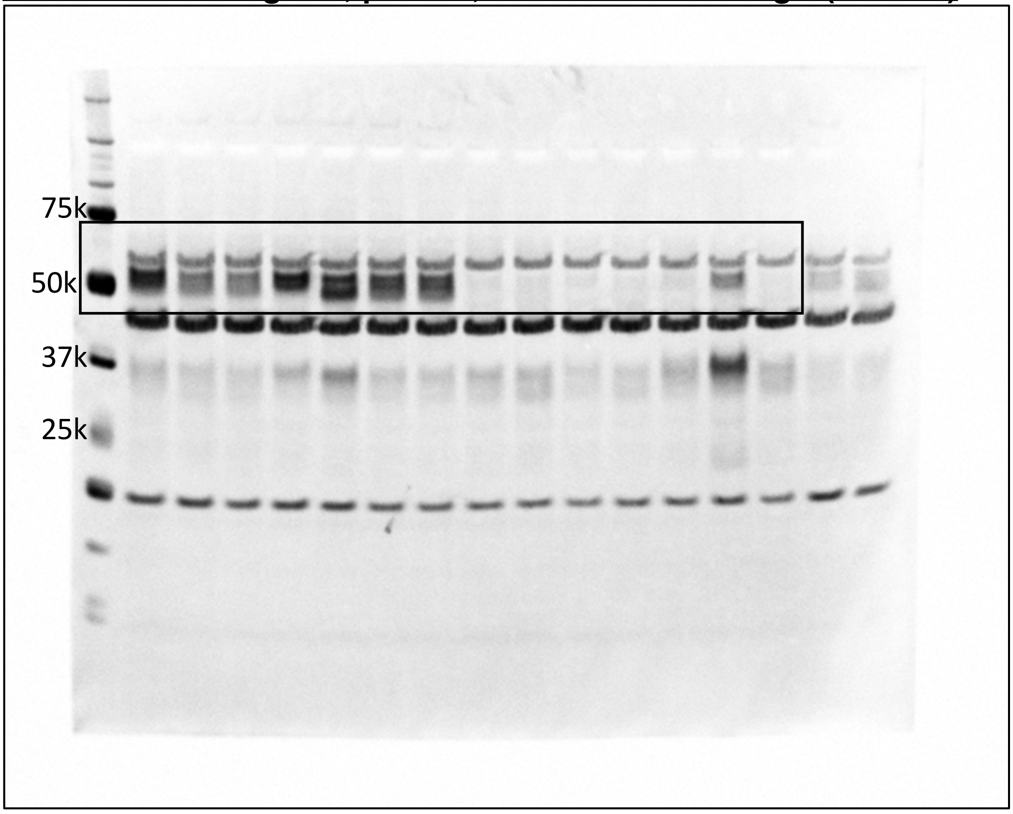

Extended Data Fig 10e, panel 4, CELA3B (separate membrane)

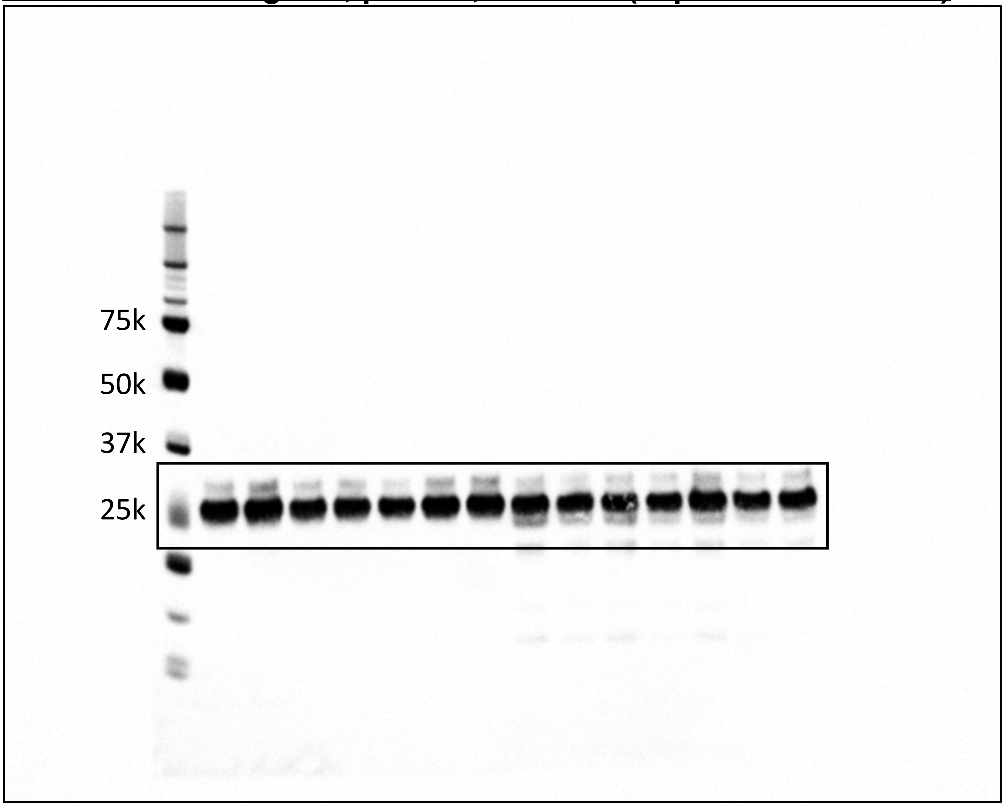

Extended data Fig 12a, rmPRSS2

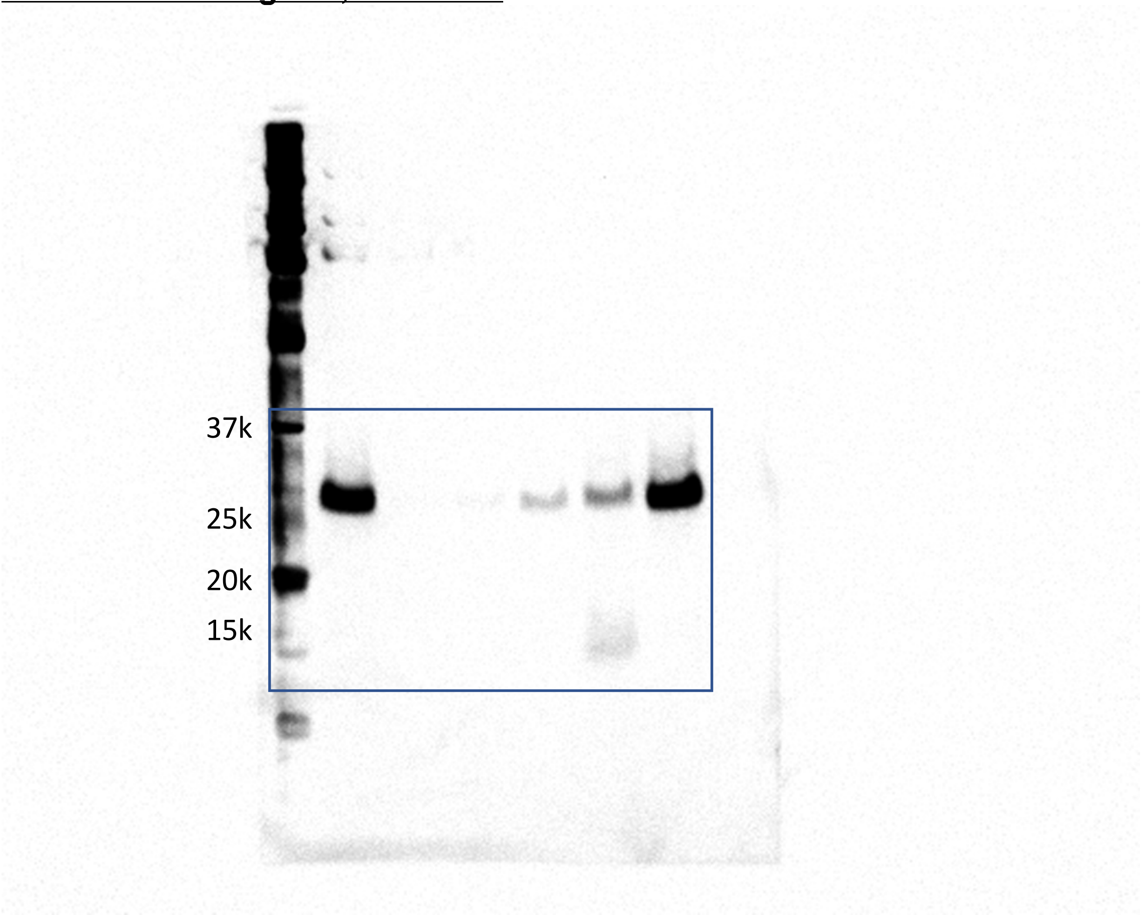

Supplement: Supplementary file 1 — Uncropped images used to prepare the main and extended data figures. [file 41586_2022_5181_MOESM1_ESM.pdf]
