# Supplementary material for: Identification of trypsin-degrading commensals in the large intestine
Source: Nature. 2022 Sep 7;609(7927):582–9. doi: 10.1038/s41586-022-05181-3 (PMC9477747; doi:10.1038/s41586-022-05181-3)
Supplement: Supplementary file 2 — Reporting Summary [file 41586_2022_5181_MOESM2_ESM.pdf]

nature research

Corresponding author(s): Kenya Honda

Last updated by author(s): 12-07-2022

Reporting Summary

Nature Research wishes to improve the reproducibility of the work that we publish. This form provides structure for consistency and transparency in reporting. For further information on Nature Research policies, see our Editorial Policies and the Editorial Policy Checklist.

Statistics

For all statistical analyses, confirm that the following items are present in the figure legend, table legend, main text, or Methods section.

n/a	Confirmed	
☐	☒	The exact sample size (*n*) for each experimental group/condition, given as a discrete number and unit of measurement
☐	☒	A statement on whether measurements were taken from distinct samples or whether the same sample was measured repeatedly
☐	☒	The statistical test(s) used AND whether they are one- or two-sided<br>*Only common tests should be described solely by name; describe more complex techniques in the Methods section.*
☐	☒	A description of all covariates tested
☐	☒	A description of any assumptions or corrections, such as tests of normality and adjustment for multiple comparisons
☐	☒	A full description of the statistical parameters including central tendency (e.g. means) or other basic estimates (e.g. regression coefficient) AND variation (e.g. standard deviation) or associated estimates of uncertainty (e.g. confidence intervals)
☐	☒	For null hypothesis testing, the test statistic (e.g. *F*, *t*, *r*) with confidence intervals, effect sizes, degrees of freedom and *P* value noted<br>*Give P values as exact values whenever suitable.*
☒	☐	For Bayesian analysis, information on the choice of priors and Markov chain Monte Carlo settings
☒	☐	For hierarchical and complex designs, identification of the appropriate level for tests and full reporting of outcomes
☒	☐	Estimates of effect sizes (e.g. Cohen's *d*, Pearson's *r*), indicating how they were calculated

Our web collection on statistics for biologists contains articles on many of the points above.

Software and code

Policy information about availability of computer code

Data collection	Light Cycler 480 software version 1.5.1 was used for collecting qPCR data.<br>PerkinElmer 2030 Manager was used for collecting protease and trypsin activity data.
Data analysis	GraphPadPrism v8.0 and Excel for Mac version 16.16.27 (201012) were used for statistical analysis.<br>For proteome analysis of caecal contents, all shotgun-MS files were searched against the mouse UniProt reference proteome (Proteome ID UP000000589, reviewed, canonical, https://www.uniprot.org/proteomes/UP000000589) using ProteinPilot software v. 4.5 with the Paragon algorithm (Sciex) for protein identification. The identified proteins were quantified from SWATH-MS data using PeakView v.2.2 (Sciex).<br>For proteome analysis of P. clara culture supernatant, an isolation width for MS2 was set to 4 m/z and overlapping window patterns in 500-780 m/z were used window placements optimized by Skyline. MS files were searched against a P. clara spectral library using Scaffold DIA (Proteome Software, Inc., Portland, OR). The spectral library was generated from P. clara protein sequence databases by Prosit. Peptide quantification was calculated by EncyclopeDIA algorithm in Scaffold DIA.<br>For peptidome analysis of P. clara incubated caecal contents, MS files were searched against the mouse UniProt reference proteome (Proteome ID UP000000589, reviewed, canonical, https://www.uniprot.org/proteomes/UP000000589) by PEAKS Studio.<br>For In-gel digestion and LC-MS/MS analysis, MS files were searched against the P. clara protein sequence database with human PRSS2 sequence (https://www.uniprot.org/uniprotkb/P07478/entry) using PEAKS Studio.<br>Metagenomes from human stool samples from PRISM, HMP2, FHS, 500FG, CVON and Jie were de novo assembled into a non-redundant gene catalogue, compiled into metagenomic species using MSPminer. To search in the gene catalogue for the homologs of P. clara and P. xylanphila genes from the trypsin associated locus containing genes 00502 and 00509, as well six other neighboring genes, we employed USEARCH 50 ublast (at protein level) retaining hits with a minimum e-value of 0.1.<br>For 16s analysis, UCLUST (https://www.drive5.com/) was used to construct OTUs. Taxonomy was assigned to each OTUs by search against the National Center for Biotechnology Information (NCBI) using the GLSEARCH program. Bacterial whole-genome sequencing was prepared using TruSeq DNA PCR-Free kit, FASTX-toolkit v0.0.13, SMRTbell template prep kit 2.0, and Canu v1.8. Sequences were assembled using Unicycler v0.4.8 and annotated using the Rapid Annotations based on Subsystem Technology (RAST) Prokaryotic Genome Annotation Server and Prokka: rapid prokaryotic genome annotation software tool.

Bacterial genome sequencing was performed by the whole-genome shotgun strategy supported by PacBio Sequel and Illumina MiSeq sequencing platforms. TruSeq DNA PCR-Free kit was used to prepare the library of the Illumina Miseq 2 x 300bp paired-end sequencing with target length = 550bp, and FASTX-toolkit (hannonlab.cshl.edu/fastx_toolkit) was used to trim and filter all the MiSeq reads with a >20 quality value (QV). SMRTbell template prep kit 2.0 was used to generate the library of the PacBio Sequel sequencing with target length = 10 - 15kbp without DNA shearing. Error correction of the trimmed reads was conducted by Canu (v1.8) with additional options (corOutCoverage = 10,000, corMinCoverage = 0, corMhapSensitivity = high) following internal control removal and adaptor trimming by Sequel. De novo hybrid assembly of the filter-passed MiSeq reads and the corrected Sequel reads was performed by Unicycler (v0.4.8), including a check of overlapping and circularization, and a circular contig was generated. Rapid Annotations based on Subsystem Technology (RAST) server and Prokka software tool were used for gene prediction and annotation of the generated contig. Default parameters were used for all software unless specified otherwise.
00502 models were predicted using AlphaFold2 through ColabFold– an online platform for protein folding. Model confidence was evaluated through pLDDT scores with a pLDDT > 90 considered as very high model confidence. The resulting AlphaFold models were then aligned in PyMOL (Schrödinger) and visualized in ChimeraX.
To evaluate which individuals in the COVID-19 cohort carried P. clara's gene 00502 or its homologues, we quality controlled stool metagenomic data using Trim_Galore! to detect and remove sequencing adapters (minimum overlap of 5 bp) and KneadData v.0.7.2 to remove human DNA contamination and trim low-quality sequences (HEADCROP:15, SLIDINGWINDOW:1:20), and retained reads that were at least 50 bp long. Paired-end quality filtered reads were mapped to the same gene catalogue from a previous study with BWA, filtered to include strong mappings with at least 95% sequence identity over the length of the read, counted and normalized to transcript-per-million (TPM matrix). Detection (TMP>0) of any of the 00502 homologs classified the sample as containing a 00502 gene in their gut microbiome. All metagenomic samples in the COVID-19 cohort had at least 8 million reads after quality filtering.

For manuscripts utilizing custom algorithms or software that are central to the research but not yet described in published literature, software must be made available to editors and reviewers. We strongly encourage code deposition in a community repository (e.g. GitHub). See the Nature Research guidelines for submitting code & software for further information.

Data

Policy information about availability of data

All manuscripts must include a data availability statement. This statement should provide the following information, where applicable:

- Accession codes, unique identifiers, or web links for publicly available datasets
- A list of figures that have associated raw data
- A description of any restrictions on data availability

The sequenced Paraprevotella genome (accession code: DRA014249) and the 16S rRNA sequence data (accession code: DRA013874) are deposited in the DNA Data Bank of Japan. Metagenomic data of the COVID-19 cohort are deposited in NCBI under BioProject PRJNA821237. Proteomics and peptidomics data are deposited in the ProteomeXchange Consortium via the jPOST partner repository (ID: PXD027678 and PXD032242). Publicly available datasets of the mouse proteome database (https://www.uniprot.org/proteomes/UP000000589) and human PRSS2 protein sequence (https://www.uniprot.org/uniprotkb/P07478/entry) were used in this study.

Field-specific reporting

Please select the one below that is the best fit for your research. If you are not sure, read the appropriate sections before making your selection.

☒ Life sciences ☐ Behavioural & social sciences ☐ Ecological, evolutionary & environmental sciences

For a reference copy of the document with all sections, see nature.com/documents/nr-reporting-summary-flat.pdf

Life sciences study design

All studies must disclose on these points even when the disclosure is negative.

Sample size

No statistical methods were used to predetermine sample size. For the human cohorts, no sample size calculation was conducted as we were not testing for end clinical outcomes nor testing any intervention. Sample sizes therefore represent the maximum number of samples we could obtain during the recruitment period. The number of animals studied per treatment group was based on our previous knowledge of the reproducibility, balancing statistical robustness and animal welfare.

Data exclusions

No data were excluded.

Replication

For all experiments involving animals or human subjects, except for the MHV experiments in Fig. 4d-f, data from a single experiment were shown with the number of mice/subject used in each experiment clearly indicated in the figure panels.
For the MHV experiments Fig. 4d-f, pooled data from three independent experiments were presented .
For all the remaining experiments (except for Fig. 3i, Extended data Fig. 2c, 3b, 5b, 6a, 6b, 6e, 9e, 10a, 12c, which were conducted once), reproducibility was verified by conducting the experiment at least twice, which yielded comparable results.
Extended data Fig. 5b, 6a & 9e involve genotyping of the bacteria, the technique (PCR) is rudimentary and the results were conclusive with a single experiment.
Whenever possible, hypotheses were verified by multiple types of experiments/data. For example, Fig. 3i (TEM data) was additionally validated by our Western (Extended data Fig. 6a) and confocal data (Fig. 3h); Extended data Fig. 2c (Western data) was additionally validated by trypsin activity assay (Fig. 2b-e, g); Extended data Fig. 6b (Western data) was additionally validated by the confocal data (Fig. 3h) and supported by the in vivo data (Fig. 4a, b); Extended data Fig. 6e (Western data) was further supported by Fig. 4j and Extended data 12a, c (P. rara, P. rodentium and P. muris do not carry genes 00503-00508 yet are capable of degrading trypsin). Western data in Fig. 10a (left panel) was validated by trypsin activity assay (Fig. 10a, right panel). Confocal data in Fig. 12c were additionally supported by the Western data (Fig. 2j, Extended data Fig. 12a)

Randomization	For the human cohorts no random allocation was used as our study was observational and did not test any intervention. For animal studies, mice were randomized into separate cages upon arrival from the vendor. Sex-matched littermates were used and the experiments were designed to test a single variable.
Blinding	For the human cohorts no blinding was performed as our study was observational and did not test any intervention. The remaining experiments were designed to test a single variable therefore blinding was not relevant.

Reporting for specific materials, systems and methods

We require information from authors about some types of materials, experimental systems and methods used in many studies. Here, indicate whether each material, system or method listed is relevant to your study. If you are not sure if a list item applies to your research, read the appropriate section before selecting a response.

Materials & experimental systems

n/a	Involved in the study	
☐	☒	Antibodies
☐	☒	Eukaryotic cell lines
☒	☐	Palaeontology and archaeology
☐	☒	Animals and other organisms
☐	☒	Human research participants
☒	☐	Clinical data
☒	☐	Dual use research of concern

Methods

n/a	Involved in the study	
☒	☐	ChIP-seq
☒	☐	Flow cytometry
☒	☐	MRI-based neuroimaging

Antibodies

Antibodies used	Antibodies used in this study are as follows: Rabbit anti-PRSS2 antibody (LSBio LS-C296077), Alexa 488-labelled goat anti-rabbit IgG (ThermoFisher Scientific #A11008), Rabbit anti-mouse PRSS2 (Cosmo Bio Co., Ltd., CPA, Japan, custom-made), Rabbit anti-mouse HSP90 antibody (#4877, clone C45G5, Cell Signaling TECHNOLOGY), Rabbit anti-human PRSS2 (LS-B15726, LSBio), Rabbit anti-human PRSS1 (LS-331381, LSBio), Rabbit anti-mouse TMPRSS2 (LS-C373022, LSBio, raised against a sequence at the protease domain), Rabbit anti-6-His Antibody [A190-214A, Bethyl laboratories, to probe His-tagged recombinant mouse PRSS2 (rmPRSS2) and human PRSS3 (hPRSS3)], Goat anti-mouse IgA alpha-chain (HRP) (ab97235, Abcam), Rat anti-mouse kappa-chain (HRP) (ab99632, Abcam), Rabbit anti-mouse CELA3b (OACD03205, Avivasysbio), Anti-rabbit IgG (HRP-linked Antibody) (#7074, Cell Signaling TECHNOLOGY), Rabbit anti-mouse Reg3beta (51153-R005, Sino Biological). Rabbit anti-6-His Antibody (A190-214A, Bethyl laboratories) was used to probe rmPRSS2 throughout the study except in Fig. 3j, where rabbit anti-mouse PRSS2 (Cosmo Bio Co., Ltd., CPA, Japan, custom-made) was used to differentiate rmPRSS2 from recombinant 00502 and 00509 (also His-tagged).
Validation	All primary antibodies were carefully validated in house with the specific bands at the expected molecular weights confirmed. Rabbit anti-PRSS2 antibody (LSBio LS-C296077) was validated by Western blot using mouse faecal samples prior to its use for immunofluorescence. Rabbit anti-mouse PRSS2 (Cosmo Bio Co., Ltd., CPA, Japan, custom-made) was validated by Western blot using mouse faecal samples (Fig. 1c, 4b). Rabbit anti-mouse HSP90 antibody (#4877, clone C45G5, Cell Signaling TECHNOLOGY) was validated by Western blot using mouse pancreas lysates (Fig. 1f). Rabbit anti-human PRSS2 (LS-B15726, LSBio) and Rabbit anti-human PRSS1 (LS-331381, LSBio) were validated by Western blot using recombinant human PRSS2 and PRSS1, respectively (Fig. 2i). Rabbit anti-mouse TMPRSS2 (LS-C373022, LSBio, raised against a sequence at the protease domain), Goat anti-mouse IgA alpha-chain (HRP) (ab97235, Abcam), Rat anti-mouse kappa-chain (HRP) (ab99632, Abcam), Rabbit anti-mouse Reg3beta (51153-R005, Sino Biological) and Rabbit anti-mouse CELA3b (OACD03205, Avivasysbio) were validated by Western using mouse faecal samples (Fig. 4b). Rabbit anti-6-His Antibody [A190-214A, Bethyl laboratories] was validated by Western using recombinant mouse PRSS2 and human PRSS3 (both his-tagged, Fig. 2f, i, j).

Eukaryotic cell lines

Policy information about cell lines

Cell line source(s)	MDCK cell line from ATCC
Authentication	The cell line was supplied and certified by ATCC and was immediately used for the experiment upon receipt to avoid any comtamination.
Mycoplasma contamination	The cell line was verified to be mycoplasma negative by the supplier.
Commonly misidentified lines (See ICLAC register)	N/A

Animals and other organisms

Policy information about studies involving animals; ARRIVE guidelines recommended for reporting animal research

Laboratory animals	C57BL/6N mice maintained under SPF or germ-free conditions were purchased from Sankyo Laboratories Japan, SLC Japan, Charles River Japan or CLEA Japan. Gnotobiotic mice were maintained within the gnotobiotic facility of RIKEN IMS. 8-15 weeks old SPF and

	germ-free WT male and female mice were used in this study. Sex-matched littermates were used in all experiments. All animals were maintained on the 12-hour light-dark cycle and received gamma-irradiated (50 kGy) pellet food (CMF, Oriental Yeast). Temperature of 20-24°C and humidity 40-60% were used for the housing conditions.
Wild animals	The study did not involve wild animals.
Field-collected samples	The study did not involve samples collected from the field.
Ethics oversight	All animal experiments were approved by the Animal Care and Use Committee of RIKEN Yokohama Institute.

Note that full information on the approval of the study protocol must also be provided in the manuscript.

Human research participants

Policy information about studies involving human research participants

Population characteristics	This study used faecal samples from healthy human donors and IBD patients, as well as faecal samples from COVID-19 patients.<br>For healthy human donors, 41 participants were recruited. Age 23-56 yo (average 34.5 yo). 29 male and 12 female.<br>For the IBD patients, we used 39 subjects (5 CD patients & 34 UC patients). Age 17-78 yo (average 48.5 yo), 27 male & 12 female.<br>For the COVID-19 cohort, 146 patients were recruited. Age 17-79 yo (average age of 50.95 yo). 93 male and 53 female.
Recruitment	For the healthy control group, volunteers were recruited by posting information leaflets or e-mailing through institutional mailing lists of Keio University School of Medicine and RIKEN Yokohama institute. Subjects (all Japanese residents) were eligible for the study if they were over the age of 20 and provided written, informed consent. Subjects were not eligible if they underwent antibiotic exposure in the previous month. We worked to ensure gender balance in the recruitment of human subjects.<br><br>For patients with IBD, we recruited patients (all Japanese) with gastrointestinal diseases or suspected gastrointestinal diseases who visited the Department of Gastroenterology of Osaka City University. Participants were informed about the significance and methods of the study prior to participation, and their consent to participate was obtained. The endoscopy was done to diagnose disease and assess disease activity. We worked to ensure gender balance in the recruitment of human subjects.<br><br>The COVID-19 cohort was recruited as a part of the Japan COVID-19 Task Force (JCTF) study. We recruited 146 patients who were diagnosed as COVID-19 by physicians using the clinical manifestation and PCR test results and hospitalized at Keio University Hospital from March 2020 to September 2021. Approximately two months after discharge from the hospital, faecal samples were collected and sent to the laboratory in DNA/RNA Shield (Zymo Research), following a protocol approved by the Institution Review Board of Keio University School of Medicine (code 20190337).Informed consent was obtained from each subject.<br><br>There was no bias towards selection of any particular group.
Ethics oversight	For collection of human faecal samples for gnotobiotic studies and for the comparison of faecal trypsin activity between IBD patients and healthy controls, human faecal samples were collected at RIKEN Institute (code H30-4, for patients with IBD) and Keio University (code 20150075, for healthy donors) according to the study protocols approved by the institutional review boards. Informed consent was obtained from each subject.<br>For the COVID-19 cohort, a protocol approved by the Institution Review Board of Keio University School of Medicine (code 20190337) was followed. Informed consent was obtained from each subject.

Note that full information on the approval of the study protocol must also be provided in the manuscript.
