# Supplementary material for: Identification of trypsin-degrading commensals in the large intestine
Source: Nature. 2022 Sep 7;609(7927):582–9. doi: 10.1038/s41586-022-05181-3 (PMC9477747; doi:10.1038/s41586-022-05181-3)
Supplement: Supplementary file 8 — Peer Review File [file 41586_2022_5181_MOESM8_ESM.docx]

**Peer Review File**

**Manuscript Title: Identification of trypsin-degrading commensals in the large intestine**

**Reviewer Comments & Author Rebuttals**

**Reviewer Reports on the Initial Version:**

Referee expertise:

Referee #1: mucosal immunology

Referee #2: microbiota-virus interactions

Referee #3: microbial metabolites, metabolomics

Referee #4: TMPRSS2, viral entry

Referees' comments:

Referee #1 (Remarks to the Author):

Li and coworkers report that trypsin is regulated via degradation by members of Paraprevotella species. They isolated Paraprevotella strains from the microbiome of healthy donors as potent trypsin-degrading commensals. Paraprevotella clara/xylaniphila recruits trypsin to the bacterial surface via T9SS-dependent polysaccharide-anchoring proteins and promote trypsin autolysis. Colonization with these strains inhibits lethal infection with murine hepatitis virus, a coronavirus dependent on trypsin and trypsin-like proteases for entry into host cells.

Investigating microbial degradation of digestive enzymes is an interesting direction that has not been well studied. The mouse experiments are well-designed, controlled, and the results are clear. The combined use of gnotobiotics, microbial genetics, and biochemistry provide strong support for their conclusions that goes well beyond the current state of the field. The genes that they identified are really poorly annotated, so their structures and activities are likely completely novel, which adds to the impact this paper will have on the field.

Minor point:

- Most blots (e.g. Fig1f, 2f/j, 3a/c/e/g/j, 4c) were only visible if using Adobe Acrobat (but not apple preview)- final figures edits should try to remediate this issue.

-----------------------------------------------------------------------------------------------------------------------------------------

Referee #2 (Remarks to the Author):

This manuscript by Li et al. explores the role of trypsin and trypsin-degrading Paraprevotella strains in regulation of enteric viral infection by murine hepatitis virus. The authors convincingly demonstrate enhanced levels of trypsin protease in the cecal and fecal contents of germ-free mice, that is not associated with increased production in pancreas or levels in the small intestine, supporting a microbiota-dependent degradation/suppression of trypsin levels in the distal intestine. Healthy human microbial communities were screened and manipulated with antibiotics to identify a community enriched in trypsin-degrading bacteria, from which 35 isolates were cultured. The authors narrow to 14, then 9, then 3-member communities, finally identifying a single Paraprevotella clara isolate sufficient to degrade trypsin alone, and finding that other Paraprevotella strains, but not Prevotella strains, have a similar trypsin-degradaing capacity. Inhibition of glycan-mediated binding to

trypsin by administration of tunicamycin or 2-F-Fuc prevents trypsin degradation, leading to implication of the T9SS as being necessary for trypsin degradation. Further screening of proteins increased in the supernatant after tunicamycin treatment reveals two additional required factors for degradation, 502 and 509. 502 attached to microbeads is shown to be sufficient to facilitate recruitment and likely autodegradation by trypsin though 509 is not. Colonization of GF mice with communities -/+ WT Paraprevotella or 502/509 mutants demonstrates 502 is required for in vivo degradation of trypsin, and IgA degradation as well as enhanced MHV infection are explored as relevant in vivo effects for trypsin regulation.

Overall, this is a well-written, carefully-executed, and really beautiful set of studies which convincingly demonstrate a great deal of the mechanism involved in a single microbe’s regulation of intestinal trypsin levels. The authors do a nice job of pointing to some important functional effects of this trypsin regulation by some limited studies looking at IgA levels and MHV infection, though these are less thoroughly explored. Some clarifications/expansions of the presented work would be important for completeness.

1. In Figure 2, trypsin degradation by multiple Paraprevotella strains is demonstrated. It would be helpful to confirm at least a subset of downstream mechanisms/assays observed with Paraprevotella clara in Paraprevotella xylaniphila strains (e.g. accumulation of fluorescent trypsin on bacterial surface) to clarify whether mechanisms are conserved.

2. Please provide in vitro growth data for the various bacterial mutants with genes implicated in trypsin degradation (PorU, WecA, insertional and targeted mutants for 502 and 509) – how do these defects affect bacterial fitness and was this ruled out as a potential confounder for loss of trypsin degradation capacity?

3. While absolute abundance of P. clara administered to GF mice in the 3-member community is demonstrated to be unaffected by presence or absence of 502/509, it would be helpful to also show the community composition in these mice by relative abundance as well as overall bacterial load – does the community of the 3- or 34-mix change +/- trypsin degradation? This seems especially important to fully interpret the IgA and MHV infection data.

4. The IgA data, especially in comparison to the rest of the data presented, seems somewhat premature to draw such a strong conclusion, as IgA levels in the intestine are sensitive to both differential inductive and degradative influences and there could be multiple factors at play here. Can the authors support their assertion by demonstrating similar results using more simplified in vitro cultures?

5. While MHV infection serves as a nice read-out of trypsin activity in the GF system and demonstrates some potential in vivo implications, it is a little unclear how relevant this mechanism is in the normal MHV-susceptible mouse host. In comparable assays, do SPF mice fall into the “protected” or “susceptible” bin? Conversely, with a growing number of studies analyzing SARS-CoV-2 infection (including enteric) and correlation of severity/symptoms to microbiota composition, are there any published/available data that might be leveraged to determine whether Paraprevotella carriage is actually protective in humans?

Minor:

1. In the title, “murine” coronavirus should be specified. Also, not sure the title is really capturing the main mechanistic thrust of the paper, which is a great study independent of any relationship to the omnipresent topic of coronavirus infection.

2. Extended Figure 9 “f” should be “d” in the figure.

3. Are there any Paraprevotella strains that have been isolated/sequenced that lack 502/509 and/or other components identified required for trypsin degradation?

-----------------------------------------------------------------------------------------------------------------------------------------

Referee #3 (Remarks to the Author):

I enjoyed this manuscript. The work described in Figures 1-3 was impressive; the in vivo antibiotic treatment, strain isolation, bioinformatics, and n-mix experiments in germ-free mice to identify P. clara as the trypsin degrader were very convincing. I was impressed by the biochemical and molecular genetic work the authors did to to narrow down to 00502 as a gene involved in trypsin degradation. This portion of the story is excellent.

But I have two related concerns, one minor and one major.

1. Minor concern: I am not sure why trypsin was chosen as the focus of this study from among the other proteins shown in Figure 1a. I presume it has something to do with the fact that trypsin is well known, but I worry that it was driven more by convenience than by a conviction that trypsin - among all the other luminal proteins - is doing something important.

2. My major concern is the lack of a convincing biological function for trypsin degradation by P. clara. I get that 2-mix+WT has a survival advantage over 2-mix+00502 in MHV infection, but I wonder whether trypsin degradation by the microbiome has a material effect on viral infection in real life. Most fecal communities have some trypsin degrading organisms, and I'm not convinced that differences in degree of trypsin degradation in humans impact the course of viral infection.

Given the conservation of the 00502 locus in Paraprevotella, it is more likely that trypsin degradation plays an important role in creating a metabolic or physical niche for P. clara. I wonder whether wild-type would be more fit than 00502 in the context of a complex community? I would be more convinced by that experiment or something like it.

-----------------------------------------------------------------------------------------------------------------------------------------

Referee #4 (Remarks to the Author):

Li and colleagues investigated the regulation of intestinal trypsin in mouse models. In brief, they report that Paraprevotella strains can induce trypsin autolysis and reveal the underlying molecular mechanism. Further, the presence of the respective bacteria is correlated with relative resistance to infection with mouse hepatitis virus 2 (MHV-2), a coronavirus that can use trypsin for the activation of its spike protein. Finally, the respective Paraprevotella strains and their relevant gene products were detected in the human microbiome.

This is an interesting and elegant study. However, regarding the MHV-2 infection experiment some questions remain open:

1. Is the S protein of the MHV-2 studied cleaved by murine PSS2?

2. Figure 4G shows plaque forming units (i.e. the number of infectious particles): It is unclear what PFUe (instead of PFU) means.

3. According to material and methods viral genome copies (and not the infectious particles) were measured. How can the authors deduce plaque forming units from genome copies?

4. MHV strains are known to differ widely in cell and organ tropism. Does the virus used for the infection experiment infect the intestine?

5. The increased infection of liver and brain and the increased number of infectious MHV-2 particles in faeces of animals lacking trypsin-degrading bacteria are supposedly the consequence of increased viral spread in the intestine (which is where trypsin degradation occurs). However, direct evidence for such a scenario is lacking. Thus, is there more infectious virus in the intestine of animals lacking trypsin-degrading bacteria and, ideally, is increased S protein cleavage observed? Are both WT and mutant mice fully susceptible to infection if virus is not applied orally (but for instance IV) or if an MHV variant is used that does not target the intestine?

**Author Rebuttals to Initial Comments:**

We are deeply grateful to the editor and reviewers for their invaluable comments, which were thorough, thoughtful, and constructive. Based on these comments, we have performed additional experiments and made the necessary revisions to the manuscript. All new changes to the text since the last review are marked in blue. We hope that the new data and experiments have sufficiently addressed the reviewer’s comments and further increased the novelty value of this work. For each specific point, the referee’s comment is in bold-italics and our response is in plain type.

*Referee #1* *(Remarks to the Author):*

*Li and coworkers report that trypsin is regulated via degradation by members of Paraprevotella species. They isolated Paraprevotella strains from the microbiome of healthy donors as potent trypsin-degrading commensals. Paraprevotella clara/xylaniphila recruits trypsin to the bacterial surface via T9SS-dependent polysaccharide-anchoring proteins and promote trypsin autolysis. Colonization with these strains inhibits lethal infection with murine hepatitis virus, a coronavirus dependent on trypsin and trypsin-like proteases for entry into host cells.*

*Investigating microbial degradation of digestive enzymes is an interesting direction that has not been well studied. The mouse experiments are well-designed, controlled, and the results are clear. The combined use of gnotobiotics, microbial genetics, and biochemistry provide strong support for their conclusions that goes well beyond the current state of the field. The genes that they identified are really poorly annotated, so their structures and activities are likely completely novel, which adds to the impact this paper will have on the field.*

We thank the reviewer for highlighting the novelty and importance of our study, and we fully agree that it will be extremely informative to carry out further structural/activity analyses for the genes we have identified. We are pleased to present additional evidence that 00502, the most essential component for trypsin degradation, is indeed special. By means of native PAGE electrophoresis we show that 00502 has both a monomer and an oligomer form in its native state (new **Extended data Fig. 9e**), and that trypsin could complex with both forms, resulting in new bands containing both proteins that could be detected by Western blot (by both 00502 and trypsin antibodies) (new **Extended data Fig. 9f**), and by in-gel M/S analysis (new **Supplementary Table 3**). We propose that oligomerization of 00502 possibly brings multiple trypsin molecules in close proximity to one another on the surface of bacteria (or when coupled with microbeads), thereby facilitating trypsin autolysis. We have adjusted our model accordingly to incorporate these new insights (new **Extended data Fig. 10a**).

In addition, by leveraging the recently developed AI tool AlphaFold2, we were able to predict the structure of 00502 with high confident scores. We found that 00502s from different trypsin-degrading species have a similar architecture, with multiple Ig-like domains forming a clustering zone which potentially serves as the binding site for trypsin (new **Extended data Fig. 10b-g**). We understand that the predictions need to be validated by conventional approaches such as crystallography or cryoEM, nevertheless we hope that the reviewer finds these preliminary structural insights sensible and of interest.

*Minor point:*

*- Most blots (e.g. Fig1f, 2f/j, 3a/c/e/g/j, 4c) were only visible if using Adobe Acrobat (but not apple preview)- final figures edits should try to remediate this issue.*

We thank the reviewer for pointing out this potential problem. It appears to be related to the individual computers/software used for viewing these blot figures. We have regenerated the figures to resolve the problem. In addition, we will work with the editors to make sure that this issue is resolved before publication.

-----------------------------------------------------------------------------------------------------------------------------------------

*Referee #2* *(Remarks to the Author):*

*This manuscript by Li et al. explores the role of trypsin and trypsin-degrading Paraprevotella strains in regulation of enteric viral infection by murine hepatitis virus. The authors convincingly demonstrate enhanced levels of trypsin protease in the cecal and fecal contents of germ-free mice, that is not associated with increased production in pancreas or levels in the small intestine, supporting a microbiota-dependent degradation/suppression of trypsin levels in the distal intestine. Healthy human microbial communities were screened and manipulated with antibiotics to identify a community enriched in trypsin-degrading bacteria, from which 35 isolates were cultured. The authors narrow to 14, then 9, then 3-member communities, finally identifying a single Paraprevotella clara isolate sufficient to degrade trypsin alone, and finding that other Paraprevotella strains, but not Prevotella strains, have a similar trypsin-degradaing capacity. Inhibition of glycan-mediated binding to trypsin by administration of tunicamycin or 2-F-Fuc prevents trypsin degradation, leading to implication of the T9SS as being necessary for trypsin degradation. Further screening of proteins increased in the supernatant after tunicamycin treatment reveals two additional required factors for degradation, 502 and 509. 502 attached to microbeads is shown to be sufficient to facilitate recruitment and likely autodegradation by trypsin though 509 is not. Colonization of GF mice with communities -/+ WT Paraprevotella or 502/509 mutants demonstrates 502 is required for in vivo degradation of trypsin, and IgA degradation as well as enhanced MHV infection are explored as relevant in vivo effects for trypsin regulation.*

*Overall, this is a well-written, carefully-executed, and really beautiful set of studies which convincingly demonstrate a great deal of the mechanism involved in a single microbe’s regulation of intestinal trypsin levels. The authors do a nice job of pointing to some important functional effects of this trypsin regulation by some limited studies looking at IgA levels and MHV infection, though these are less thoroughly explored. Some clarifications/expansions of the presented work would be important for completeness.*

We thank the reviewer for acknowledging the quality of our study and for helping point out the parts that could be more thoroughly explored. We have conducted additional experiments to address these important points, and we hope that the reviewer will find these new data not only reinforce the conclusions we have drawn in the previous manuscript, but also offer novel insights into this commensal-mediated trypsin degradation.

*1. In Figure 2, trypsin degradation by multiple Paraprevotella strains is demonstrated. It would be helpful to confirm at least a subset of downstream mechanisms/assays observed with Paraprevotella clara in Paraprevotella xylaniphila strains (e.g. accumulation of fluorescent trypsin on bacterial surface) to clarify whether mechanisms are conserved.*

We thank the reviewer for the constructive comment, and we fully agree that it is important to confirm whether the observed mechanisms are conserved between the trypsin-degrading strains. As per the reviewer’s suggestion we performed confocal microscope experiment and confirmed that fluorescent trypsin indeed accumulated on the surface of all the *Paraprevotella* strains in our hands (new **Extended data Fig. 15**). In addition, as we have identified new bacterial species (*P. rara*, *P. rodentium* and *P. muris*) that demonstrate trypsin degradation capacity (new **Fig. 5a, b**), we conducted similar confocal experiments and confirmed the accumulation of trypsin on the surface of these new species (new **Extended data Fig. 15**).

In addition, by leveraging the recently developed AI tool AlphaFold2, we were able to predict the structure of 00502 with high confident scores. We found that 00502s from different trypsin-degrading species have a similar architecture, with multiple Ig-like domains forming a clustering zone which potentially serves as the binding site for trypsin (new **Extended data Fig. 10b-g**). We understand that the predictions need to be validated by conventional approaches such as crystallography or cryoEM, nevertheless we hope that the reviewer finds these preliminary structural insights sensible and of interest.

Collectively, these new data all support our hypothesis that the mechanisms underlying trypsin degradation are conserved among trypsin-degrading *Paraprevotella and Prevotella* species.

*2. Please provide in vitro growth data for the various bacterial mutants with genes implicated in trypsin degradation (PorU, WecA, insertional and targeted mutants for 502 and 509) – how do these defects affect bacterial fitness and was this ruled out as a potential confounder for loss of trypsin degradation capacity?*

We thank the reviewer for pointing out the possibility that growth defects could be a potential confounding factor. In the revised manuscript we have provided new data showing that the mutants deficient in trypsin degradation (insertional mutants for *PorU*, *WecA*, *00502* and *00509*, as well as *Δ00502* and *Δ00509*) all displayed normal growth rate *in vitro* (new **Extended data Fig. 7c**). In addition, quantification of faecal *P. clara* DNA showed that *Δ00502* and *Δ00509*, when administered to GF mice along with the 2-mix, had normal *in vivo* growth [new **Extended data Fig. 11a (left panel), c**], excluding the possibility that the loss of trypsin degradation capacity was simply due to lack of bacterial fitness.

*3. While absolute abundance of P. clara administered to GF mice in the 3-member community is demonstrated to be unaffected by presence or absence of 502/509, it would be helpful to also show the community composition in these mice by relative abundance as well as overall bacterial load – does the community of the 3- or 34-mix change +/- trypsin degradation? This seems especially important to fully interpret the IgA and MHV infection data.*

We thank the reviewer for raising this important issue that deletion of *00502*/*00509* may affect the community composition, making the IgA/MHV data potentially less straightforward to interpret. As per the reviewer’s suggestion we analyzed the overall bacterial load by qPCR using a universal bacterial 16S rRNA primer pair. We found no difference between the 2-mix+WT/*Δ00502*/*Δ00509* groups, or between the 34-mix+WT/*Δ00502* groups (new **Extended data Fig. 11b**).

To characterize the community composition, for the 2-mix+*WT*/ *Δ00502*/ *Δ00509* experiment we used qPCR to quantify the DNA from each member and calculated relative abundance of the three species in the faeces. We found that deletion of *00502* or *00509* had little impact on the relative abundance of the individual members (new **Extended data Fig 11c**). It is worth noting that *Δ00502* and *Δ00509* colonized the mice as effectively as the *WT* strain under this less competitive condition (new **Extended data Fig 11a, left panel**). For the 34-mix+*WT*/*Δ00502* experiment, since it was less practical to design primers to quantify all the members, we examined their relative abundance by 16S rRNA sequencing. We found there was no major difference in the microbial composition between the 34-mix+*WT* and 34-mix+ *Δ00502* groups (new **Extended data Fig 11d**). Interestingly, *P. clara* turned out to be the only one out of the 35 members that demonstrated a significant difference (*WT* strain was more abundant than *Δ00502*) in the 16S rRNA sequencing analysis. We quantified faecal *P. clara* DNA by qPCR and found that consistent with our 16S analysis, *WT* *P. clara* was significantly more abundant than the *Δ00502* strain in this competitive setting (new **Extended data Fig 11a, right panel**).

Our data suggest that changes in trypsin levels do not markedly affect the overall bacterial load or community composition *in vivo*, at least in our experimental settings. However, the ability to degrade trypsin may confer *P. clara* a competitive advantage in the context of a more complex microbiota. We hope that the reviewer finds these new data informative.

*4. The IgA data, especially in comparison to the rest of the data presented, seems somewhat premature to draw such a strong conclusion, as IgA levels in the intestine are sensitive to both differential inductive and degradative influences and there could be multiple factors at play here. Can the authors support their assertion by demonstrating similar results using more simplified in vitro cultures?*

We absolutely agree with the reviewer that it is vital to differentiate the inductive and degradative influences on IgA levels. To support our assertion, we carefully designed an *ex vivo* experiment to exclude the possibility of differential IgA induction as the confounding factor (new **Extended data Fig. 12a**): faeces from the 2-mix+*WT* *P. clara* colonized mice were filtered (to remove the bacteria), diluted and incubated with faeces from GF mice (also filtered and with the same dilution). Note that the initial 2-mix+*WT* faeces contained high IgA and low trypsin whilst GF faeces contained low IgA and high trypsin (new **Extended data Fig. 12a**). After incubation for 24 hours the IgA heavy chain, but not the *kappa* light chain, was considerably reduced (new **Extended data Fig. 12a**). The whole reaction took place with no mice or bacteria involved, at the same time reasonably simulated the interactions between the faecal proteins (trypsin and IgA) at the physiological condition. We could affirm that the heavy chain is indeed sensitive to trypsin degradation. In line with this assertion, when the mixture was pretreated with TLCK, a trypsin-specific inhibitor, the degradation of IgA heavy chain was completely blocked (new **Extended data Fig. 12a**).

The sensitivity of IgA heavy chain (but not the *kappa* light chain) to trypsin degradation was further confirmed by *ex vivo* incubation of the 2-mix+*WT* faeces with recombinant mouse trypsin (rmPRSS2): IgA heavy chain was degraded by rmPRSS2, in a similar way as by faecal trypsin from GF mice (new **Extended data Fig. 12a**).

We can now comfortably draw the conclusion that the heavy chain of IgA is sensitive to trypsin degradation, and that the low IgA heavy chain levels we observed in the 2-mix+ *Δ00502* colonized mice were likely due to excessive degradation by trypsin, rather than differential IgA induction. Our *in vivo* and *ex vivo* finding that the *kappa* light chain was much less affected by differential trypsin levels also supports this statement (**Fig. 4b,** new **Extended data Fig. 12a**). We hope that the reviewer will agree that our earlier conclusion has now been greatly substantiated by our new *ex vivo* findings.

*5. While MHV infection serves as a nice read-out of trypsin activity in the GF system and demonstrates some potential in vivo implications, it is a little unclear how relevant this mechanism is in the normal MHV-susceptible mouse host. In comparable assays, do SPF mice fall into the “protected” or “susceptible” bin? Conversely, with a growing number of studies analyzing SARS-CoV-2 infection (including enteric) and correlation of severity/symptoms to microbiota composition, are there any published/available data that might be leveraged to determine whether Paraprevotella carriage is actually protective in humans?*

To examine the effect of *P. clara*-mediated trypsin degradation in the context of a complex microbiota, we prepared 34-mix+*WT* and 34-mix+ *Δ00502* gnotobiotic mice, and infected them with MHV-2 by intragastric gavage. GF+34-mix+*WT* mice were more resistant to MHV-2 infection than GF+34mix+ *Δ00502* mice (new **Fig. 4h)**. Our new data suggest that *Paraprevotella*-mediated trypsin degradation contributes to the resistance to MHV-2 infection via the intestinal route in a more physiologically relevant setting.

We agree with reviewer that it would be informative to assess the susceptibility of SPF mice to MHV infection, considering that the SPF mice kept in our animal facility (RIKEN SPF) also have low trypsin levels in the lower intestine (**Fig. 1g**). The low trypsin levels of RIKEN SPF mice are possibly associated with the presence of a different trypsin-degrading species, *P. rodentium*, in their intestinal microbiome (new **Fig. 5b**, new **Extended data Fig. 14d**). However, it is extremely difficult to design an SPF experiment with proper controls. We attempted to compare the sensitivity to MHV-2 of RIKEN SPF with that of GF+34-mix+*WT* and GF+34-mix+ *Δ00502* mice. RIKEN SPF mice showed intermediate resistance to MHV-2 (see right **Figure panel for the reviewer only**). Since the microbiota compositions were completely different and many factors other than trypsin could contribute to the difference between SPF and GF+34-mix+*WT* mice, we opted to leave the results of SPF mice out from the manuscript.

As per the reviewer’s suggestion, we also examined publicly available metagenome sequencing datasets of COVID-19 patients, although currently available datasets on COVID-19 are somewhat limited. Consistent with our notion, one of the published studies, Tao et al.^1^ showed that *Paraprevotella* spp. were negatively associated with persistent intestinal infection of SARS-Cov2 and gastrointestinal symptoms. We also analyzed the COVID-19 cohort published in Yeoh et al.^2^, which contains one of the largest metagenome datasets (~100 patients). We determined prevalence values of *P. clara*, *P. xylaniphila*, and seven 00502 encoders and observed a trend towards lower prevalence of 00502 encoders with more severe disease (see right **Figure panel for the reviewer only**).

In addition, we newly recruited and analyzed 146 COVID-19 subjects from Keio University Hospital in Tokyo (new **Supplementary Table 4**) to provide independent data (which are more comfortable for us than datasets published elsewhere). We conducted metagenome sequencing of faecal samples from the individuals who recovered from COVID-19 (faeces collected after the discharge from the hospital). Among the 146 volunteers, the diarrhoea scores were available for 141 cases from the health care record. 62 (44%) out of the 141 subjects experienced diarrhoea during hospitalization. We examined association of the carriage of *00502* homologue genes in the gut microbiome with the disease severity and diarrhoea frequency by stratifying cases by the presence or absence of *00502* homologues. Among the *00502* homologue-negative individuals, there was a significantly higher frequency of patients experiencing severe diarrhoea (more than 1 day with more than 2 diarrhoeal episodes per day) (*p* < 0.05, Fisher's test, one-sided) (new **Figure 5c**). In addition, we found that the absence of *00502* homologues in the gut microbiome was significantly associated with a higher rate of oxygen inhalation (*p* < 0.05, Fisher's test, one-sided) (new **Figure 5d**). These results are consistent with our hypothesis that trypsin-degrading commensal colonization may provide protective benefits against SARS-CoV2 infection. Needless to say, we realise that there are a number of limitations in our metagenome analysis. In particular, because of the small number of subjects, the data were unadjusted for known confounders such as age, sex, and comorbidities. The causal relationship between trypsin degradation and the protection against SARS-CoV2 infection needs to be further validated by larger cohorts and additional animal models. Therefore, we also discussed these limitations in the Discussion of the manuscript.

*Minor:*

*1. In the title, “murine” coronavirus should be specified. Also, not sure the title is really capturing the main mechanistic thrust of the paper, which is a great study independent of any relationship to the omnipresent topic of coronavirus infection.*

We thank the reviewer’s comment. We have changed our title to ‘Identification of commensals and molecular components mediating trypsin degradation in the large intestine’ to capture the major findings in this paper.

*2. Extended Figure 9 “f” should be “d” in the figure.*

We thank the reviewer for pointing this out – we have corrected the mistake.

*3. Are there any Paraprevotella strains that have been isolated/sequenced that lack 502/509 and/or other components identified required for trypsin degradation?*

All the *Paraprevotella* strains that we have tested in this study can facilitate trypsin degradation (**Fig. 2j**) and have the same 00502-00509 gene cluster in their genomes (**Extended data Fig. 8a**).

In the revised manuscript we also present new data related to three 00502-carrying *Prevotella* isolates (*P. rara*, *P. rodentium* and *P. muris*), which we obtained and tested after our first submission. *P. rara* (or MSP 0081) was identified in our original mining of a de novo-assembled gut microbiome gene catalogue from 6 geographically diverse cohorts (new **Fig. 5a,** new **Extended data Fig. 14a**). *P. rodentium* and *P. muris* were identified by mining 00502 homologues in NCBI’s non-redundant (NR) database using Blastp (protein-protein BLAST) (new **Fig. 5a**). All the three species have been confirmed to possess trypsin-degrading capacity (new **Fig. 5b**). Notably, *P. rara* and *P. rodentium* carry both 00502 and 00509 homologues in their genomes, whilst *P. muris* carries a 00502 homologue but lacks 00509 (new **Fig. 5a**). These data are consistent with our previous conclusion that 00502 is absolutely essential for trypsin degradation (all 00502-carriers we have tested so far can facilitate trypsin degradation), whilst 00509 plays a supportive and dispensable role.

On a related note, in our revised manuscript we have assigned MSP 0081 and MSP 0224 to their proper names, *Prevotella rara* and *Prevotellamassilia timonensis* (new **Fig. 5a**, new **Extended data 14 a-c**), after confirming the identity between our assembled metagenome sequences and the sequencing data uploaded by the researchers who originally identified them^3,4^. We apologize for any confusion this might cause.

-----------------------------------------------------------------------------------------------------------------------------------------

*Referee #3* *(Remarks to the Author):*

*I enjoyed this manuscript. The work described in Figures 1-3 was impressive; the in vivo antibiotic treatment, strain isolation, bioinformatics, and n-mix experiments in germ-free mice to identify P. clara as the trypsin degrader were very convincing. I was impressed by the biochemical and molecular genetic work the authors did to narrow down to 00502 as a gene involved in trypsin degradation. This portion of the story is excellent. But I have two related concerns, one minor and one major.*

We thank the reviewer for the positive comments about our work. The concerns raised by the reviewer are pertinent and we have undertaken the following revisions/experiments to address these concerns.

*1. Minor concern: I am not sure why trypsin was chosen as the focus of this study from among the other proteins shown in Figure 1a. I presume it has something to do with the fact that trypsin is well known, but I worry that it was driven more by convenience than by a conviction that trypsin – among all the other luminal proteins – is doing something important.*

Trypsin activity has been associated with disturbance of gut homeostasis^5-7^. This is supported by our own observation that fecal samples from both humans with inflammatory bowel disease (IBD) and IL-10-deficient mice with colitis displayed higher trypsin activities (new **Extended data Fig. 1b, c**). Therefore, out of the 45 proteins listed in **Fig. 1a**, trypsin naturally drew our attention. Whether or not trypsin is doing something important was exactly what motivated this study: to precisely answer this question we had to identify the species & molecules responsible for trypsin degradation and generate the defective mutants.

*2. My major concern is the lack of a convincing biological function for trypsin degradation by P. clara. I get that 2-mix+WT has a survival advantage over 2-mix+00502 in MHV infection, but I wonder whether trypsin degradation by the microbiome has a material effect on viral infection in real life. Most fecal communities have some trypsin degrading organisms, and I’m not convinced that differences in degree of trypsin degradation in humans impact the course of viral infection.*

We fully understand the reviewer’s concern. To address the concern, we conducted several new experiments:

1. The experiment with 2-mix+*WT* vs 2-mix+*Δ00502* mice may represent a non-physiological condition. Therefore, we examined the effect of *P. clara*-mediated trypsin degradation in the context of a complex microbiota. We prepared gnotobiotic mice colonized with 34-mix+*WT* and 34-mix+ *Δ00502*, and orally infected them with MHV-2. GF+34-mix+*WT* mice were significantly more resistant to MHV-2 infection than GF+34-mix+ *Δ00502* mice. Our data suggest that *Paraprevotella*-mediated trypsin degradation contributes to the resistance to MHV infection in a more physiologically relevant setting. We have included these data in new **Fig. 4h**.

2. We newly recruited 146 patients with COVID-19 at Keio University Hospital in Tokyo (new **Supplementary Table 4**) and collected their faecal samples after the discharge from the hospital. Among the 146 subjects, the diarrhoea scores during hospitalization were available for 141 cases. Among the 141, 62 (44%) experienced diarrhoea as one of the COVID-19 symptoms. We conducted metagenome sequencing of the faecal samples and examined the association of the carriage of 00502 homologue genes with the disease severity and diarrhoea frequency by stratifying cases by the presence or absence of 00502 homologues. Among the 00502 homologue-negative individuals, there was a significantly higher frequency of patients experiencing severe diarrhoea (more than 1 day with more than 2 diarrhoeal episodes per day) (*p* < 0.05, Fisher's test, one-sided) (new **Figure 5c)**. In addition, we found that the absence of 00502 homologues was associated significantly with a higher rate of oxygen inhalation (*p* < 0.05, Fisher's test, one-sided) (new **Figure 5d**). These results are consistent with our hypothesis that trypsin-degrading commensal colonization may provide protective benefits against SARS-CoV2 infection. However, there are a number of limitations in our metagenome analysis. In particular, because of the small number of subjects, the data were unadjusted for known confounders such as age, sex, and comorbidities. The causal relationship between trypsin degradation and the protection against SARS-CoV2 infection needs to be validated by larger cohorts and additional animal models. Therefore, we included these new data together with discussions of the limitations in the Discussion of the manuscript.

3. We also examined the biological relevance of microbiome-mediated trypsin degradation in non-viral-infection contexts:

i) Our data clearly indicate that a low trypsin environment protects IgA (the heavy chain in particular) from degradation (**Figure 4b**). Given the importance of IgA in gut homeostasis and mucosal defense, this protection is unlikely to be trivial. We confirmed the importance of trypsin degradation and IgA protection by using a vaccination and infection model of enteropathogenic bacteria (new **Extended data Fig. 12b-f**): *WT* or *Δ00502* *P. clara* colonized GF mice were first orally vaccinated with inactivated *Citrobacter rodentium*, and then orally infected with live *C. rodentium* (new **Extended data Fig. 12b**). Following *C. rodentium* vaccination and infection, *WT* *P. clara* colonized mice showed less body weight loss (new **Extended data Fig. 12c**) and fewer *C. rodentium* invaded into the cecum (new **Extended data Fig. 12d**) than *Δ00502* *P. clara* colonized mice. Examination of caecal IgA showed that following vaccination and infection, *WT* *P. clara* colonized mice had higher levels of total and *C. rodentium*-specific IgA in the caecal contents (new **Extended data Fig. 12e**), and the caecal contents from these mice induced more pronounced bacterial agglutination when incubated with *in vitro* cultured live *C. rodentium* (new **Extended data Fig. 12f**). These data suggest that trypsin degradation and IgA protection are beneficial for the host, enabling a more effective response against previously encountered enteropathogens.

ii) We examined the relevance (benefit) of trypsin degradation for the ‘trypsin degraders’. Our new results indicate that degradation of trypsin confers *P. clara* an advantage under competitive conditions (see our response to **comment 3** below). The exact mechanism underlying this competitive advantage remains elusive, though we speculate that trypsin recruitment and autolysis on bacterial surface enable the ‘trypsin degraders’ to more effectively acquire and assimilate amino acids from the degraded trypsin peptides. This makes perfect biological sense: after passing through the small intestine trypsin is of little use and potentially damaging (due to its proteolytic effect) for the host, meantime when degraded it can serve as a good nutrient source for the commensals.

Collectively our data indicate that this microbiome-mediated trypsin degradation is beneficial for both the bacteria and for the host: it enables the trypsin-degrading species to compete more effectively with other members in the intestine, and protects the host from intestinal viral infections as well as the important defense molecules (at least IgA) from excessive degradation. We hope the reviewer will agree with us that the commensal-mediated trypsin degradation in the large intestine is relevant and potentially represents a symbiotic relationship that has been less appreciated in the microbiota field.

*3. Given the conservation of the 00502 locus in Paraprevotella, it is more likely that trypsin degradation plays an important role in creating a metabolic or physical niche for P. clara. I wonder whether wild-type would be more fit than 00502 in the context of a complex community? I would be more convinced by that experiment or something like it.*

We thank the reviewer for raising this important possibility that carriage of *00502* and degradation of trypsin may give *Paraprevotella* a competitive advantage, and we apologize for having not addressed this point in the previous manuscript. We now provide two lines of evidence that *WT P. clara* is, as the reviewer expected, more fit than the *Δ00502* strain under competitive conditions.

1. We conducted 16S rRNA sequencing analysis of the faeces from mice colonized with 34-mix+*WT* or 34-mix+ *Δ00502* (new **Extended data Fig. 11d**). We found that whilst the composition of the 34-mix showed little difference between the 2 groups, the *WT* strain was significantly more abundant than the *Δ00502* strain (new **Extended data Fig. 11d**). Indeed, *P. clara* turned out to be the only one out of the 35 members that demonstrated a significant difference (*WT* strain was more abundant than *Δ00502*). This was validated by our qPCR analysis of faecal *P. clara* DNA (new **Extended data Fig. 11a, right panel**).

2. We administered both *WT* and *Δ00502* stains to the same GF mice (together with the 2-mix, namely 4-mix experiment), and carefully designed qPCR primers to specifically quantify the faecal DNA from either strain (new **Extended data Fig. 11e**). Our quantification showed that the *WT* strain showed modest competitive advantage as early as on Day 1, and that by Day 14 the *WT* strain nearly completely outcompeted the *Δ00502* strain (new **Extended data Fig. 11f, g**).

We hope that these data will convince the reviewer that the ability to degrade trypsin confers the *WT* strain an advantage under competitive conditions.

-----------------------------------------------------------------------------------------------------------------------------------------

*Referee #4* *(Remarks to the Author):*

*Li and colleagues investigated the regulation of intestinal trypsin in mouse models. In brief, they report that Paraprevotella strains can induce trypsin autolysis and reveal the underlying molecular mechanism. Further, the presence of the respective bacteria is correlated with relative resistance to infection with mouse hepatitis virus 2 (MHV-2), a coronavirus that can use trypsin for the activation of its spike protein. Finally, the respective Paraprevotella strains and their relevant gene products were detected in the human microbiome.*

*This is an interesting and elegant study. However, regarding the MHV-2 infection experiment some questions remain open:*

*1. Is the S protein of the MHV-2 studied cleaved by murine PSS2?*

We have chosen MHV-2 strain as it is the only MHV that has uncleaved S-protein^8^. Other MHV strains express already cleaved (fusion-ready) S-proteins and can induce cell-cell fusion without any proteolytic activation. In contrast, MHV-2 needs to undergo proteolytic cleavage of S-protein to fuse with cells^8, 9^, much like SARS-CoV and SARS-CoV2^10-12^. Trypsin is known to be sufficient to induce proteolytic cleavage/activation of the S-proteins of both MHV-2 and SARS-CoV^13^. We have cited these important references and explained this unique property of MHV-2 more clearly in the revised manuscript.

*2. Figure 4G shows plaque forming units (i.e. the number of infectious particles): It is unclear what PFUe (instead of PFU) means.*

*3. According to material and methods viral genome copies (and not the infectious particles) were measured. How can the authors deduce plaque forming units from genome copies?*

We apologize for the unclear labelling of the viral load. We did not determine the viral titer, as MHV is highly contagious in laboratory mice, RIKEN institute where we conducted mouse infection experiments basically did not allow us to conduct a plaque forming assay. We thus quantified virus RNA levels in tissues and cells by RT-qPCR.

As per the comment and taking this opportunity, we re-evaluated the accuracy of our virus RNA quantification method. In particular, we evaluated the effect of different templates on generating standard curves for the copy number calculation of MHV-2. We compared RNA extracted from PFU-predetermined MHV-2 and a copy-number-predetermined plasmid inserted with cDNA of viral ORF1a. We confirmed that serial dilutions of these templates produced almost equivalent standard curves in terms of slope, goodness of fit, and efficiency (see right **Figure panel for the reviewer only).** As one PFU may not represent one copy of viral RNA, we have chosen the plasmid-based method to generate standard curves. Therefore, in the revised manuscript, we re-preformed RT-qPCR for all the samples and calculated the copy numbers of MHV-2 RNA based on standard curves generated in each run using the plasmid. We now show these new data of “MHV-2 RNA copies/ whole tissue” in new **Fig 4e** and “MHV-2 RNA copies/well” in new **Extended data** **Fig 13c** in the revised manuscript.

*4. MHV strains are known to differ widely in cell and organ tropism. Does the virus used for the infection experiment infect the intestine?*

In the MHV-2 infection experiments, we introduced MHV-2 into the stomach/duodenum of mice via intragastric gavage using a long (4 cm) catheter to make sure enteric infection by MHV-2. Therefore, in our experimental setting, virus transmission to the liver and brain occurred most likely from the intestine. We apologize that we did not clearly describe the procedure of MHV-2 infection in our previous submission. Therefore, we have made necessary amendments in the main text and the method section.

In addition, we examined whether MHV-2 was able to infect mouse intestinal epithelial cells. We first confirmed that CEACAM1, the principal receptor for MHV-2, was expressed at high levels in epithelial organoid cells derived from murine intestine (particularly from the large intestine) (new **Extended Data Fig 13b**). Moreover, MHV-2 infected murine colonic epithelial organoid cells, which was further enhanced by the presence of trypsin (new **Extended Data Fig 13c**). In contrast, MHV-2 failed to infect dog-derived MDCK cells which are widely used for the propagation of human influenza viruses (new **Extended Data Fig 13c**). MDCK cells are dog-derived and express dog CEACAM1 showing very low homology to rodent CEACAM1.

In addition, we examined MHV-2 infection in a mouse intestinal cell line CMT93. The DBT cell line (which is routinely used for MHV-2 propagation) and the MDCK cell line were used as the positive and negative control respectively. Effective propagation of MHV-2 in CMT93 cells was clearly demonstrated by plague assay (see right **Figure panel A, for the reviewer only**) and immunofluorescence staining (see right **Figure panel B, for the reviewer only,** MHV-2 was stained green**)**

These new data are consistent with the previous reports and indicate that the virus strain used for the infection experiments in our study is capable of infecting the mouse intestine.

*5. The increased infection of liver and brain and the increased number of infectious MHV-2 particles in faeces of animals lacking trypsin-degrading bacteria are supposedly the consequence of increased viral spread in the intestine (which is where trypsin degradation occurs). However, direct evidence for such a scenario is lacking. Thus, is there more infectious virus in the intestine of animals lacking trypsin-degrading bacteria and, ideally, is increased S protein cleavage observed? Are both WT and mutant mice fully susceptible to infection if virus is not applied orally (but for instance IV) or if an MHV variant is used that does not target the intestine?*

Because of its contagious nature, the logistics of faecal samples from MHV-infected mice was quite a challenge. Moreover, as faecal suspension contains components toxic to mammalian cells^14^, it was extremely difficult to determine whether there are more infectious virus particles in the faeces from animals lacking trypsin-degrading bacteria (we gave best efforts possible for more than 6 months but failed).

However, we successfully show that both GF+2mix+*WT* *P. clara* and GF+2mix+*Δ00502* *P. clara* mice were fully susceptible to MHV-2 infection when the virus was applied via the intraperitoneal route: i.e., there was no difference in survival between these two groups. Therefore, these results support our model in which trypsin-degrading commensals prevent intestinal infection and consequent systemic spread of MHV-2. We have included these new data in new **Fig 4i**.

References

1	Tao, W. *et al.* Re-detectable positive SARS-CoV-2 RNA tests in patients who recovered from COVID-19 with intestinal infection. *Protein & cell* **12**, 230-235 (2021).

2	Yeoh, Y. K. *et al.* Gut microbiota composition reflects disease severity and dysfunctional immune responses in patients with COVID-19. *Gut* **70**, 698-706 (2021).

3	Efimov, B. A. *et al.* Prevotella rara sp. nov., isolated from human faeces. *International journal of systematic and evolutionary microbiology* **68**, 3818-3825 (2018).

4	Ndongo, S., Lagier, J.-C., Fournier, P.-E., Raoult, D. & Khelaifia, S. “Prevotellamassilia timonensis,” a new bacterial species isolated from the human gut. *New microbes and new infections* **13**, 102 (2016).

5	Hansen, K. K. *et al.* A major role for proteolytic activity and proteinase-activated receptor-2 in the pathogenesis of infectious colitis. *Proceedings of the National Academy of Sciences* **102**, 8363-8368 (2005).

6	Midtvedt, T. *et al.* Increase of faecal tryptic activity relates to changes in the intestinal microbiome: analysis of Crohn's disease with a multidisciplinary platform. *PLoS One* **8**, e66074 (2013).

7	Jablaoui, A. *et al.* Fecal serine protease profiling in inflammatory bowel diseases. *Frontiers in cellular and infection microbiology* **10**, 21 (2020).

8	Qiu, Z. *et al.* Endosomal proteolysis by cathepsins is necessary for murine coronavirus mouse hepatitis virus type 2 spike-mediated entry. *Journal of virology* **80**, 5768-5776 (2006).

9	Matsuyama, S. & Taguchi, F. Two-step conformational changes in a coronavirus envelope glycoprotein mediated by receptor binding and proteolysis. *Journal of virology* **83**, 11133-11141 (2009).

10	Ou, X. *et al.* Characterization of spike glycoprotein of SARS-CoV-2 on virus entry and its immune cross-reactivity with SARS-CoV. *Nature communications* **11**, 1-12 (2020).

11	Jaimes, J. A., Millet, J. K. & Whittaker, G. R. Proteolytic cleavage of the SARS-CoV-2 spike protein and the role of the novel S1/S2 site. *IScience* **23**, 101212 (2020).

12	Xia, S. *et al.* The role of furin cleavage site in SARS-CoV-2 spike protein-mediated membrane fusion in the presence or absence of trypsin. *Signal transduction and targeted therapy* **5**, 1-3 (2020).

13	Kawase, M., Kataoka, M., Shirato, K. & Matsuyama, S. Biochemical analysis of coronavirus spike glycoprotein conformational intermediates during membrane fusion. *Journal of virology* **93**, e00785-00719 (2019).

14	Gonzalez-Hernandez, M. B., Cunha, J. B. & Wobus, C. E. Plaque assay for murine norovirus. *JoVE (Journal of Visualized Experiments)*, e4297 (2012).

**Reviewer Reports on the First Revision:**

Referees' comments:

Referee #1 (Remarks to the Author):

The authors should be commended by their efforts.

The competitive colonization experiment (Extended data 11f-g) is really interesting and could be the start of a new project.

The human data is also exciting and opens up many new questions and directions.

-----------------------------------------------------------------------------------------------------------------------------------------

Referee #2 (Remarks to the Author):

The authors have effectively responded to my prior concerns and the manuscript is substantially strengthened.

-----------------------------------------------------------------------------------------------------------------------------------------

Referee #3 (Remarks to the Author):

The authors did a very nice job responding to my comments and those of the other referees, and the new data are convincing. I would therefore support accepting the manuscript in its current form.

-----------------------------------------------------------------------------------------------------------------------------------------

Referee #4 (Remarks to the Author):

This is an interesting study and the new experiments have further improved it. However, two minor and a major point remain open.

Major

The major concept is that trypsin degradation reduces infection of the intestine and limits viral spread to other organs, liver and brain. The new data show that the MHV-2 strain used indeed infects intestinal organoids. However, data showing that MHV-2 spread in the intestine is reduced if trypsin degradation is operative are still lacking (qRT-PCR data) and should be provided.

Minor

My previous question “Is the S protein of the MHV-2 studied cleaved by murine PSS2?” was not sufficiently precise. My apologies. It is my understanding that MHV-2 is efficiently activated by human trypsin in cell culture conditions. My question is whether there is also direct evidence that murine trypsin (which might harbor some amino acid exchanges relative to human trypsin) activates MHV-2 spike.

“Colonization with WT P. clara protected mice from viral spread into the liver and brain”. This reviewer disagrees since viral RNA was detected in these organs. One could state “Colonization with WT P. clara reduced viral spread into the liver and brain”.

**Author Rebuttals to First Revision:**

*Referee #4 (Remarks to the Author):*

*This is an interesting study and the new experiments have further improved it. However, two minor and a major point remain open.*

*Major*

*The major concept is that trypsin degradation reduces infection of the intestine and limits viral spread to other organs, liver and brain. The new data show that the MHV-2 strain used indeed infects intestinal organoids. However, data showing that MHV-2 spread in the intestine is reduced if trypsin degradation is operative are still lacking (qRT-PCR data) and should be provided.*

We apologise for not being able to provide the RT-qPCR data of MHV-2 in the intestine. Due to the highly contagious nature of MHV, we were only allowed to execute all our *in vivo* experiments in our stainless-steel isolators, specially designed for our MHV-2 experiments (see the right image). It is extremely challenging to operate inside these rather compact isolators with a restricted size of window for the operators to see through. The collected tissues had to be preserved in the DNA/RNA shield buffer for RNA isolation (which also inactivates infectious agents including MHV-2), before they could be transferred out of the isolators. Technically, we found it extremely difficult to collect intestinal epithelial cells (where MHV-2 is most likely to infect and replicate primarily) within these isolators.

As a proxy of intestinal epithelial propagation of MHV-2, we quantified the copy number of MHV-2 in faeces. We examined the faecal MHV-2 levels in GF+2-mix+WT or *D00502* *P. clara* mice and GF+34-mix+WT or *D00502* *P. clara* mice over the course of the experiments. In both experimental settings, WT *P. clara* colonized mice showed significantly lower faecal MHV-2 levels, suggesting that MHV-2 infection and expansion in the intestine are reduced if trypsin degradation is operative. We included these new data in new **Fig 4d** and **4g** in the revised manuscript.

It is noteworthy that the MHV-2 levels in the faeces were highest on day 1 and decreased thereafter (see **the right Figure**). In contrast, in the liver and brain, MHV-2 was not detected on days 1 and 2 (data not shown), but was detected at elevated levels on days 4 and 5. Therefore, MHV-2 levels in the liver and brain do not merely reflect viral loads in the intestine. MHV-2 may infect yet-unidentified intestinal cells at an early stage of infection (which is likely inhibited by trypsin-degrading microbes) without severely damaging the intestinal epithelium. The virus then enters the systemic circulation, infects and expands in the liver and brain, and ultimately causes death in mice. It is likely that initial viral amounts that enter the systemic circulation from the intestine have a significant influence on the subsequent course of the disease. We modified our claim in the manuscript to reflect these new data, and additional investigation is certainly necessary to understand the sequence of events resulting from the MHV-2 infection and how trypsin-degrading bacteria affect it. We hope the reviewer will agree with us that any additional experiments to confirm the details of the disease mechanism of MHV-2 are beyond the scope of this study and would take at least another manuscript worth of experiments to accomplish.

*Minor*

*My previous question “Is the S protein of the MHV-2 studied cleaved by murine PSS2?” was not sufficiently precise. My apologies. It is my understanding that MHV-2 is efficiently activated by human trypsin in cell culture conditions. My question is whether there is also direct evidence that murine trypsin (which might harbor some amino acid exchanges relative to human trypsin) activates MHV-2 spike.*

We thank the reviewer for the clarification of the question.

Trypsin is known to be highly conserved between different species. Trypsins from different sources share similar sequences and structure with the strictly conserved catalytic triad His57, Asp 102 and Ser195 (see also below figure), and all trypsins specifically cleave the peptide bond on the carboxyl side of lysine or arginine residues^1,2^.

Because of its conserved substrate specificity (targeting specific amino acids rather than proteins), few studies distinguish the source of trypsin. Trypsin extracted from bovine pancreas, which is commercially available in relatively large quantities (mg range), is often used. Most of the recombinant human or mouse trypsin are only available at the µg range which is not sufficient for cell culture experiments. The bovine trypsin we used (not human trypsin) is treated with L-1-tosylamido-2-phenylethyl chloromethyl ketone (TPCK) to inhibit contaminating chymotrypsin activity without affecting trypsin activity (We apologize for not being clear about the characteristics of trypsin we used in the experiment; we have included these information in the Materials&Methods). As a matter of fact, bovine trypsin was used in earlier studies demonstrating that trypsin cleaves the spike protein of MHV-2 and facilitates membrane fusion^3,4^. Bovine trypsin was also used in the reports showing that trypsin facilitates the entry of SARS-CoV^5,6^. Considering the fact that MHV-2 is a murine coronavirus, we feel it is reasonable to deduce that murine trypsin activates MHV-2 under natural conditions. We hope the reviewer will agree that the current level of mechanistic insight is appropriate and that the details of MHV-2 biology are beyond the scope of this study.

*“Colonization with WT P. clara protected mice from viral spread into the liver and brain”. This reviewer disagrees since viral RNA was detected in these organs. One could state “Colonization with WT P. clara reduced viral spread into the liver and brain”.*

We apologise for the overstatement and have modified the sentence as the reviewer suggested.

References

1	Rypniewski, W. R., Perrakis, A., Vorgias, C. E. & Wilson, K. S. Evolutionary divergence and conservation of trypsin. *Protein Engineering, Design and Selection* **7**, 57-64 (1994).

2	Lemieux, H. & Blier, P. Trypsin activity measurement in fish and mammals: comparison of four different methods. *Journal of Aquatic Food Product Technology* **16**, 13-26 (2007).

3	Qiu, Z. *et al.* Endosomal proteolysis by cathepsins is necessary for murine coronavirus mouse hepatitis virus type 2 spike-mediated entry. *Journal of virology* **80**, 5768-5776 (2006).

4	Matsuyama, S. & Taguchi, F. Two-step conformational changes in a coronavirus envelope glycoprotein mediated by receptor binding and proteolysis. *Journal of virology* **83**, 11133-11141 (2009).

5	Simmons, G. *et al.* Inhibitors of cathepsin L prevent severe acute respiratory syndrome coronavirus entry. *Proceedings of the National Academy of Sciences* **102**, 11876-11881 (2005).

6	Matsuyama, S., Ujike, M., Morikawa, S., Tashiro, M. & Taguchi, F. Protease-mediated enhancement of severe acute respiratory syndrome coronavirus infection. *Proceedings of the National Academy of Sciences* **102**, 12543-12547 (2005).

**Reviewer Reports on the Second Revision:**

Referees' comments:

Referee #4 (Remarks to the Author):

The authors have adequately addressed the points raised by this reviewer.
